# Testing the efficacy and safety of BIO101, for the prevention of respiratory deterioration, in patients with COVID-19 pneumonia (COVA study): a structured summary of a study protocol for a randomised controlled trial

**DOI:** 10.1186/s13063-020-04998-5

**Published:** 2021-01-11

**Authors:** W. Dioh, M. Chabane, C. Tourette, A. Azbekyan, C. Morelot-Panzini, L. A. Hajjar, M. Lins, G. B. Nair, T. Whitehouse, J. Mariani, M. Latil, S. Camelo, R. Lafont, P. J. Dilda, S. Veillet, S. Agus

**Affiliations:** 1 Biophytis SA, Sorbonne Université – BC9, 4 place Jussieu, 75005 Paris, France; 2 Biophytis, Inc, 210 Broadway, Suite #201, Cambridge, MA 02139 USA; 3 Service de Pneumologie, Médecine Intensive et Réanimation - R3S (SPMIR-R3S), Hôpital Pitié-Salpêtrière – APHP, Paris, France; 4 Universidade de São Paulo Instituto do Coração, São Paulo, SP Brasil; 5 General Hospital Sint-Maarten, Mechelen, Belgium; 6 Oakland University William Beaumont School of Medicine, Royal Oak, MI USA; 7 Queen Elizabeth Hospital Birmingham, Birmingham, B15 2GW UK; 8 Sorbonne Université, CNRS - Institut de Biologie Paris Seine (B2A), 75005 Paris, France; 9 Sorbonne Université, CNRS - Institut de Biologie Paris Seine (BIOSIPE), 75005 Paris, France

**Keywords:** COVID-19, SARS-CoV2, randomised controlled trial, Renin Angiotensin System, Mas receptor, angiotensin converting enzyme-2, 20-hydroxyecdysone, BIO101, Inflammation, Acute respiratory Distress Syndrome

## Abstract

**Objectives:**

As of December, 1^st^, 2020, coronavirus disease 2019 (COVID-19) caused by SARS-CoV-2, resulted in more than 1 472 917 deaths worldwide and death toll is still increasing exponentially. Many COVID-19 infected people are asymptomatic or experience moderate symptoms and recover without medical intervention. However, older people and those with comorbid hypertension, diabetes, obesity, or heart disease are at higher risk of mortality. Because current therapeutic options for COVID-19 patients are limited specifically for this elderly population at risk, Biophytis is developing BIO101 (20-hydroxyecdysone, a Mas receptor activator) as a new treatment option for managing patients with SARS-CoV-2 infection at the severe stage.

The angiotensin converting enzyme 2 (ACE2) serves as a receptor for SARS-CoV-2. Interaction between ACE2 and SARS-CoV2 spike protein seems to alter the function of ACE2, a key player in the renin-angiotensin system (RAS). The clinical picture of COVID-19 includes acute respiratory distress syndrome (ARDS), cardiomyopathy, multiorgan dysfunction and shock, all of which might result from an imbalance of the RAS. We propose that RAS balance could be restored in COVID-19 patients through MasR activation downstream of ACE2 activity, with 20-hydroxyecdysone (BIO101) a non-peptidic Mas receptor (MasR) activator. Indeed, MasR activation by 20-hydroxyecdysone harbours anti-inflammatory, anti-thrombotic, and anti-fibrotic properties. BIO101, a 97% pharmaceutical grade 20-hydroxyecdysone could then offer a new therapeutic option by improving the respiratory function and ultimately promoting survival in COVID-19 patients that develop severe forms of this devastating disease. Therefore, the objective of this COVA study is to evaluate the safety and efficacy of BIO101, whose active principle is 20-hydroxyecdysone, in COVID-19 patients with severe pneumonia.

**Trial design:**

Randomized, double-blind, placebo-controlled, multi-centre, group sequential and adaptive which will be conducted in 2 parts.

Part 1:

Ascertain the safety and tolerability of BIO101 and obtain preliminary indication of the activity of BIO101, in preventing respiratory deterioration in the target population

Part 2:

Re-assessment of the sample size needed for the confirmatory part 2 and confirmation of the effect of BIO101 observed in part 1 in the target population.

The study is designed as group sequential to allow an efficient run-through, from obtaining an early indication of activity to a final confirmation. And adaptive – to allow accumulation of early data and adapt sample size in part 2 in order to inform the final design of the confirmatory part of the trial.

**Participants:**

Inclusion criteria

1. Age: 45 and above
2. A confirmed diagnosis of COVID-19 infection, within the last 14 days, prior to randomization, as determined by PCR or other approved commercial or public health assay, in a specimen as specified by the test used.
3. Hospitalized, in observation or planned to be hospitalized due to COVID-19 infection symptoms with anticipated hospitalization duration ≥3 days
4. With evidence of pneumonia based on all of the following:
  - Clinical findings on a physical examination
  - Respiratory symptoms developed within the past 7 days
5. With evidence of respiratory decompensation that started not more than 4 days before start of study medication and present at screening, meeting one of the following criteria, as assessed by healthcare staff:
  - Tachypnea: ≥25 breaths per minute
  - Arterial oxygen saturation ≤92%
  - A special note should be made if there is suspicion of COVID-19-related myocarditis or pericarditis, as the presence of these is a stratification criterion
6. Without a significant deterioration in liver function tests:
  - ALT and AST ≤ 5x upper limit of normal (ULN)
  - Gamma-glutamyl transferase (GGT) ≤ 5x ULN
  - Total bilirubin ≤ 5×ULN
7. Willing to participate and able to sign an informed consent form (ICF). Or, when relevant, a legally authorized representative (LAR) might sign the ICF on behalf of the study participant
8. Female participants should be: at least 5 years post-menopausal (i.e., persistent amenorrhea 5 years in the absence of an alternative medical cause) or surgically sterile; OR
  - Have a negative urine pregnancy test at screening
  - Be willing to use a contraceptive method as outlined in inclusion criterion 9 from screening to 30 days after last dose.
9. Male participants who are sexually active with a female partner must agree to the use of an effective method of birth control throughout the study and until 3 months after the last administration of the investigational product.

*(Note: medically acceptable methods of contraception that may be used by the participant and/or partner include combined oral contraceptive, contraceptive vaginal ring, contraceptive injection, intrauterine device, etonogestrel implant, each supplemented with a condom, as well as sterilization and vasectomy).*

10. Female participants who are lactating must agree not to breastfeed during the study and up to 14 days after the intervention.
11. Male participants must agree not to donate sperm for the purpose of reproduction throughout the study and until 3 months after the last administration of the investigational product.
12. For France only: Being affiliated with a European Social Security.

Exclusion criteria

1. Not needing or not willing to remain in a healthcare facility during the study
2. Moribund condition (death likely in days) or not expected to survive for >7 days – due to other and non-COVID-19 related conditions
3. Participant on invasive mechanical ventilation via an endotracheal tube, or extracorporeal membrane oxygenation (ECMO), or high-flow Oxygen (delivery of oxygen at a flow of ≥16 L/min.).
4. Participant is not able to take medications by mouth (as capsules or as a powder, mixed in water).
5. Disallowed concomitant medication: Consumption of any herbal products containing 20-hydroxyecdysone and derived from *Leuzea carthamoides*; *Cyanotis vaga* or *Cyanotis arachnoidea* is not allowed (e.g. performance enhancing agents).
6. Any known hypersensitivity to any of the ingredients, or excipients of the study medication, BIO101.
7. Renal disease requiring dialysis, or known renal insufficiency (eGFR≤30 mL/min/1.73 m2, based on Cockcroft & Gault formula).
8. In France only:
  - Non-affiliation to compulsory French social security scheme (beneficiary or right-holder).
  - Being under tutelage or legal guardianship.

Participants will be recruited from approximately 30 clinical centres in Belgium, France, the UK, USA and Brazil. Maximum patients’ participation in the study will last 28 days. Follow-up of participants discharged from hospital will be performed through post-intervention phone calls at 14 (± 2) and 60 (± 4) days.

**Intervention and comparator:**

Two treatment arms will be tested in this study: interventional arm 350 mg b.i.d. of BIO101 (AP 20-hydroxyecdysone) and placebo comparator arm 350 mg b.i.d of placebo. Administration of daily dose is the same throughout the whole treatment period. Participants will receive the study medication while hospitalized for up to 28 days or until a clinical endpoint is reached (i.e., ‘negative’ or ‘positive’ event). Participants who are officially discharged from hospital care will no longer receive study medication.

**Main outcomes:**

Primary study endpoint:

The proportion of participants with ‘negative’ events up to 28 days.

‘Negative’ events are defined as respiratory deterioration and all-cause mortality. For the purpose of this study, respiratory deterioration will be defined as any of the following:

- Requiring mechanical ventilation (including cases that will not be intubated due to resource restrictions and triage).
- Requiring extracorporeal membrane oxygenation (ECMO).
- Requiring high-flow oxygen defined as delivery of oxygen at a flow of ≥16 L/min.

Only if the primary endpoint is significant at the primary final analysis the following

Key secondary endpoints will be tested in that order:

- Proportion of participants with events of respiratory failure at Day 28
- Proportion of participants with ‘positive’ events at Day 28.
- Proportion of participants with events of all-cause mortality at Day 28

A ‘positive’ event is defined as the official discharge from hospital care by the department due to improvement in participant condition.

Secondary and exploratory endpoints:

In addition, a variety of functional measures and biomarkers (including the SpO2 / FiO2 ratio, viral load and markers related to inflammation, muscles, tissue and the RAS / MAS pathways) will also be collected.

**Randomization:**

Randomization is performed using an IBM clinical development IWRS system during the baseline visit. Block-permuted randomization will be used to assign eligible participants in a 1:1 ratio.

- In part 1, randomization will be stratified by RAS pathway modulator use (yes/no) and co-morbidities (none vs. 1 and above).
- In Part 2, randomization will be stratified by centre, gender, RAS pathway modulator use (yes/no), co-morbidities (none vs. 1 and above), receiving Continuous Positive Airway Pressure/Bi-level Positive Airway Pressure (CPAP/BiPAP) at study entry (Yes/No) and suspicion of COVID-19 related myocarditis or pericarditis (present or not).

**Blinding (masking):**

Participants, caregivers, and the study team assessing the outcomes are blinded to group assignment. All therapeutic units (TU), BIO101 b.i.d. or placebo b.i.d., cannot be distinguished in compliance with the double-blind process. An independent data-monitoring committee (DMC) will conduct 2 interim analyses. A first one based on the data from part 1 and a second from the data from parts 1 and 2. The first will inform about BIO101 safety, to allow the start of recruitment into part 2 followed by an analysis of the efficacy data, to obtain an indication of activity. The second interim analysis will inform about the sample size that will be required for part 2, in order to achieve adequate statistical power.

Numbers to be randomised (sample size)

Number of participants randomized: up to 465, in total

- Part 1: 50 (to obtain the proof of concept in COVID-19 patients).
- Part 2: 310, potentially increased by 50% (up to 465, based on interim analysis 2) (to confirm the effects of BIO101 observed in part 1).

**Trial Status:**

The current protocol Version is V 10.0, dated on 24.09.2020. The recruitment that started on September 1^st^ 2020 is ongoing and is anticipated to finish for the whole study by March2021.

**Trial registration:**

The trial was registered before trial start in trial registries: EudraCT, No. 2020-001498-63, registered May 18, 2020; and Clinicaltrials.gov, identifier NCT04472728, registered July 15, 2020.

**Full protocol:**

The full protocol is attached as an additional file, accessible from the Trials website (Additional file 1). In the interest in expediting dissemination of this material, the familiar formatting has been eliminated; this Letter serves as a summary of the key elements of the full protocol.

**Supplementary Information:**

The online version contains supplementary material available at 10.1186/s13063-020-04998-5.

## Supplementary Information

**Additional file 1.** Full Protocol

## Data Availability

The datasets generated and/or analysed during the current study are not publicly available due to the fact that they are the private properties of BIOPHYTIS SA.

