# Supplementary material for: Testing the efficacy and safety of BIO101, for the prevention of respiratory deterioration, in patients with COVID-19 pneumonia (COVA study): a structured summary of a study protocol for a randomised controlled trial
Source: Trials. 2021 Jan 11;22:42. doi: 10.1186/s13063-020-04998-5 (PMC7797700; doi:10.1186/s13063-020-04998-5)
Supplement: Supplementary file 1 — Additional file 1. Full Protocol [file 13063_2020_4998_MOESM1_ESM.pdf]

**Protocol title:**	Adaptive design phase 2 to 3, randomized, double-blind, multicenter, to evaluate the safety, efficacy, pharmacokinetics and pharmacodynamics of BIO101 in the prevention of the respiratory deterioration in hospitalized patients with COVID-19 pneumonia (severe stage)
**Abbreviated Title:**	Testing the Efficacy and Safety of BIO101, for the Prevention of Respiratory Deterioration, in Patients with **COV**ID-19 Pneumoni**a** (COVA study)
**Protocol Number**	BIO101-CL05
**Clinicaltrials.gov Identifier**	NCT04472728
**EudraCT Number**	2020-001498-63
**Phase**	2-3
**Coordinating Investigator**	**Prof. Capucine Morelot-Panzini**<br>Hôpital Pitié-Salpêtrière<br>47-83 boulevard de l'Hôpital<br>75013 Paris
**Sponsor**	**BIOPHYTIS S.A.**<br>14 Avenue de l'Opéra<br>75001 Paris France<br>Tel. +331 44 27 23 00
**Sponsor Signatory**	**Dr. Waly Dioh, PhD**<br>Chief Operations Officer<br>**Dr. Sam Agus, MD**<br>Chief Medical Officer
**Sponsor Medical Expert**	**Dr. Sam Agus, MD**<br>Chief Medical Officer
**Protocol Version**	10.0

The confidential information in this document is provided to you as an investigator, potential investigator or consultant for review by you, your staff and applicable Independent Ethics Committee or Review Board. It is understood that the information will not be disclosed to others without written authorization from Biophytis S.A.

Summary of Version Changes:

Date of current version: September 24, 2020

Amendment 9

Sections	Summary of Revisions	Rationale
Signature page	PI signature page was re-inserted.	PI signature page was found to be missing from previous amendment version.

Amendment 8

September 21, 2020

Sections	Summary of Revisions	Rationale
Section 1.1. Synopsis<br>Section 3.3.5.1 Overview of all study end points<br>Section 8.4.5 Analysis of Primary and Key Secondary Endpoints and Sequential Testing	A change in the order of the key-secondary end-points	To reflect that since in most cases, mortality will be preceded by intubation and therefore, it is likely that the number of cases that will have mortality registered as their primary end-points will be very few.
Section 2.1 Background	Update on the list of current treatments in investigation for COVID-19	An update, to reflect the current situation
Section 6.2.3 Storage and Stability	Shelf-life was extended to 36 months.	Based on an update of the IMPD ed. 10.
Section 6.3.2 Dissemination of Results<br>Section 7.2.9 Safety Stopping Rules for Entire Study	Changed wording on dissemination of results.	To clarify that dissemination of results will be done only by the DMC, if they deem it is needed for the interest of public health.
Section 8.2.1 Major protocol deviations	Use of RAS pathway modulators was removed as a major protocol deviation as they are listed as allowed medications.	Clarification and consistency.

The confidential information in this document is provided to you as an investigator, potential investigator or consultant for review by you, your staff and applicable Independent Ethics Committee or Review Board. It is understood that the information will not be disclosed to others without written authorization from Biophytis S.A.

Sections	Summary of Revisions	Rationale
Section 8.4.5 Analysis of Primary and Key Secondary Endpoints and Sequential Testing	It was specified that reporting of results in terms of the risk difference and associated CI and p-value.<br>the sequence of the testing of the key secondary endpoints was fixed.<br>the key secondary endpoint of negative events at Day 14 was removed (since not applicable anymore), and the key secondary endpoint of proportion of subjects with an event of all-cause mortality was added.	In accordance with to the recommendation of the FDA.

Amendment 7

August 25, 2020

Sections	Summary of Revisions	Rationale
Section 1.1 Synopsis<br>Section 5.1 Inclusion Criteria	Rewording of inclusion criterion 7 to allow for a legally authorized representative to sign the ICF (USA only)	In response to request from the central IRB dated July 28th 2020.
Section 1.1 Synopsis<br>Section 3.3.5.3.1 Endpoints for the Interim Analysis	Secondary endpoints were rearranged hierarchically	In response to request from ANVISA dated August 6th 2020.

The confidential information in this document is provided to you as an investigator, potential investigator or consultant for review by you, your staff and applicable Independent Ethics Committee or Review Board. It is understood that the information will not be disclosed to others without written authorization from Biophytis S.A.

Sections	Summary of Revisions	Rationale
Section 1.1 Synopsis<br>Section 5 Study Population<br>Section 4.1.1.1 Age and Severity	Adjustment of inclusion criteria for age.	The age group of 45-55 seems to be a significant proportion of the patients with COVID-19 and while this group has slightly better outcome than those who belong to the more elderly, it is still significantly higher than that of the younger groups.<br>Refer to Section 4.1.1.1 for more details for the adjustment of age.
Section 1.1 Synopsis<br>Section 5 Study Population<br>Section 6.5.1 Disallowed Medications	Adjustment of inclusion criteria for use of RAS pathway modulators.	To further reduce the limits on the study population. RAS modulators is a stratification factor.
Section 1.1 Synopsis<br>Section 5 Study Population	Removal of requirement for patients to be on oxygen supplementation, when screened to the study	Oxygen supplementation is not always used even when patients have a drop in peripheral saturation.
Section 1.1 Synopsis<br>Section 5 Study Population	Adding a requirement for lactating mothers not to breastfeed during the study	A needed clarification
Section 2.3 Scientific Rationale<br>Section 6.2.3 Storage and Stability<br>Section 13 References	Updated section 2.3 to better reflect the scientific rationale.<br>Shelf-life was reduced to 24 months.	Based on an update of the IMPD ed. 10.
Section 4 Study Design	Adapted wording to reflect that identification of part 1 and part 2 participants is only for the purpose of communication about the study with involved stakeholder, not for the statistical analysis.	In response to request from ANVISA dated August 6th 2020.

The confidential information in this document is provided to you as an investigator, potential investigator or consultant for review by you, your staff and applicable Independent Ethics Committee or Review Board. It is understood that the information will not be disclosed to others without written authorization from Biophytis S.A.

Sections	Summary of Revisions	Rationale
Section 7.2.4.2.3 Assessment Of Expectedness	Wording on the assessment of expectedness was adapted.	In response to feedback from the FAMHP dated August 17th 2020.
Section 1.1 Synopsis<br>Section 5.1 Inclusion Criteria<br>Section 5.2 Exclusion Criteria<br>Section 7.2 Safety Assessments<br>Section 8.3 Statistics<br>Section 9.1.4 Future Use of Stored Specimens and Data	Updates and corrections were made	Clarification and consistency throughout synopsis and body of the protocol

Amendment 6

July 17, 2020

Sections	Summary of Revisions	Rationale
Section 7.2.8.1.5 Renal Failure Stopping Criteria	Adding renal failure stopping criteria	In response to request from FDA dated July 13th 2020.
Section 7.2.2 Safety Labs	Adding fibrinogen to local lab assessments.	To align liver function test stopping criteria with lab assessments.

Amendment 5

July 9, 2020

Sections	Summary of Revisions	Rationale
Section 1.1 Synopsis<br>Section 3.3.1 Proportion and Time to Events<br>Section 12 Abbreviations and Definitions	Provision of a clearer definition to high-flow oxygen.	In response to request for clarification from study investigators

The confidential information in this document is provided to you as an investigator, potential investigator or consultant for review by you, your staff and applicable Independent Ethics Committee or Review Board. It is understood that the information will not be disclosed to others without written authorization from Biophytis S.A.

Sections	Summary of Revisions	Rationale
Section 1.2 Study Scheme<br>Section 3.3.1 Proportion and Time to Events<br>Section 4.3.4 Post-intervention Period<br>Section 5.3 Withdrawal of Participants During the Study	Adding 60-days after end of on-intervention period follow-up phone call.	In response to request from FDA on June 30th 2020 and FAMHP on July 2nd 2020.
Section 8.3 Sample Size Justification<br>Section 8.4 Description of Statistical Methods	Clarification on futility and efficacy boundaries and clarification on possible additional subgroup and sensitivity analyses.	In response to the concern that was expressed by FDA, that the futility analysis done during the part 2 interim analysis has a high-risk to identify futility, even if the is a benefit from the study medication.

Amendment 4

June 24, 2020

Sections	Summary of Revisions	Rationale
Section 1.1 Synopsis<br>Section 1.2 Study Scheme<br>Section 4 Study Design<br>Section 5 Study Population<br>Section 6.3 Measures to Reduce Bias<br>Section 6.5 Concomitant Medications<br>Section 7.3 Additional Assessments<br>Section 8.4 Description of Statistical Methods<br>Section 13 References	Adjustment of inclusion criteria for age and use of RAS pathway modulators and corresponding adjustment of stratification.	According to the discussion with the Agence Nationale de Sécurité du Médicament et des Produits de Santé (ANSM) on June 5th, 2020. Refer to Section 4.1.1.1 for more details for the adjustment of age.

The confidential information in this document is provided to you as an investigator, potential investigator or consultant for review by you, your staff and applicable Independent Ethics Committee or Review Board. It is understood that the information will not be disclosed to others without written authorization from Biophytis S.A.

Sections	Summary of Revisions	Rationale
Section 1.1 Synopsis<br>Section 1.3 Schedule of Activities<br>Section 3.3.1 Proportion and Time to Events<br>Section 3.3.5.2.2 Endpoints for the Interim Analysis for Indication of Activity, at the End of Part 1<br>Section 4 Study Design<br>Section 5 Study Population<br>Section 6.1 Dosing and Administration	Adaptation of the Part 2 design and removal of the possible extension description	According to the US Food and Drug Administration (FDA) comments received on June 19th, 2020.
Section 4 Study Design	Clarifications regarding interim analyses: their population, timing and purpose	According to the discussion with the Agence Nationale de Sécurité du Médicament et des Produits de Santé (ANSM) on June 5th, 2020.
Section 1.1 Synopsis<br>Section 1.2 Study Scheme<br>Section 3.3 Endpoints<br>Section 7.2 Safety Assessments	Addition of safety laboratory test to monitor testicular biomarkers	According to the discussion with the Agence Nationale de Sécurité du Médicament et des Produits de Santé (ANSM) on June 5th, 2020.
Section 3.3 Endpoints<br>Section 4 Study Design	Clarification on the randomization ratio: 1:1 randomization is planned for both Part 1 and Part 2. Only if the results of the interim analysis at the end of Part 1 warrant it will a 2:1 randomization scheme for Part 2 be considered and possibly implemented by amendment.	According to the discussion with the Agence Nationale de Sécurité du Médicament et des Produits de Santé (ANSM) on June 5th, 2020.

The confidential information in this document is provided to you as an investigator, potential investigator or consultant for review by you, your staff and applicable Independent Ethics Committee or Review Board. It is understood that the information will not be disclosed to others without written authorization from Biophytis S.A.

Sections	Summary of Revisions	Rationale
Section 1.1 Synopsis<br>Section 3.3 Endpoints<br>Section 7.1 Assessment of Efficacy	Clarification of the difference between the primary and key secondary endpoints for respiratory events	According to the discussion with the Agence Nationale de Sécurité du Médicament et des Produits de Santé (ANSM) on June 5th, 2020.
Section 4 Study Design<br>Section 6.3 Measures to Reduce Bias<br>Section 8.4 Description of Statistical Methods<br>Section 13 References	Addition of rationale for including participant receiving CPAP/BiPAP at screening<br>Addition of receiving CPAP/BiPAP at study entry (Yes/No) as stratification factor	According tothe discussion with the Agence Nationale de Sécurité du Médicament et des Produits de Santé (ANSM) on June 5th, 2020.
Section 6.5 Concomitant Medications	Clarification of concomitant medication section with separation of allowed and disallowed medication	According to the discussion with the Agence Nationale de Sécurité du Médicament et des Produits de Santé (ANSM) on June 5th, 2020.
Section 7.2.9 Safety Stopping Rules for the Entire Study	Increase of DMC meeting frequency	According to the discussion with the Agence Nationale de Sécurité du Médicament et des Produits de Santé (ANSM) on June 5th, 2020.
Section 2.4 Benefit/Risk Assessment<br>Section 4.1 Rationale for Study Design	Inclusion of rationale for changes	Provide rationale for decisions taken during amendment writing<br>According to the Federal agency for medicines and health products (FAMHP) comments received on June 12th, 2020

The confidential information in this document is provided to you as an investigator, potential investigator or consultant for review by you, your staff and applicable Independent Ethics Committee or Review Board. It is understood that the information will not be disclosed to others without written authorization from Biophytis S.A.

**Sections**	**Summary of Revisions**	**Rationale**
Section 1.1 Synopsis<br>Section 3.3 Endpoints<br>Section 4.3.3.3 Population PK Study<br>Section 5.1 Inclusion Criteria<br>Section 5.2 Exclusion Criteria<br>Section 7.1 Assessment of Efficacy<br>Section 7.2 Safety Assessments<br>Section 8.3 Sample Size Justification	Updates and corrections were made	Clarification and consistency throughout synopsis and body of the protocol

Amendment 3

Date: 28 May 2020

**Sections**	**Summary of Revisions**	**Rationale**
Section 1.1 Synopsis<br>Section 1.2 Study Scheme<br>Section 4 Study Design<br>Section 8.3 Sample Size Justification<br>Section 8.4 Description of Statistical Methods	Adjustment of randomization for Part 2 from 2:1 to 1:1	According to the US Food and Drug Administration (FDA) comments received on May 12th, 2020.
Section 1.1 Synopsis<br>Section 1.2 Study Scheme<br>Section 3.3 Endpoints<br>Section 4 Study Design<br>Section 8.4 Description of Statistical Methods	Clarifications regarding interim analyses and their purpose, as well as corresponding clarifications and additions to endpoints	According to the US Food and Drug Administration (FDA) comments received on May 12th, 2020.

The confidential information in this document is provided to you as an investigator, potential investigator or consultant for review by you, your staff and applicable Independent Ethics Committee or Review Board. It is understood that the information will not be disclosed to others without written authorization from Biophytis S.A.

Sections	Summary of Revisions	Rationale
Section 1.1 Synopsis<br>Section 1.3 Schedule of Activities<br>Section 2.4.4 Risks associated with the COVID-19 epidemic<br>Section 3.3 Endpoints<br>Section 4.3.4 Post-intervention Period<br>Section 7.1 Assessment of Efficacy	Addition of efficacy information collection to 14-day post-intervention phone call (including corresponding endpoint); participants reaching endpoint will not be withdrawn from study	According to the US Food and Drug Administration (FDA) comments received on May 12th, 2020.
Section 8.1 Study Cohorts<br>Section 8.4 Description of Statistical Methods	Adjustments to planned statistical analyses	According to the US Food and Drug Administration (FDA) comments received on May 12th, 2020.
Section 4.3.3.4 Endpoint and Early Withdrawal<br>Section 4.4 Definition of End-of-Study Intervention	Clarification of the distinction between intervention discontinuation and study withdrawal	According to the US Food and Drug Administration (FDA) comments received on May 12th, 2020.
Section 1.1 Synopsis<br>Section 6.3.2 Dissemination of Results	Corrections and clarifications	According to the US Food and Drug Administration (FDA) comments received on May 12th, 2020.
Section 1.1 Synopsis<br>Section 5.1 Inclusion Criteria	Addition of restrictions regarding sperm donation	According to the Medicines and Healthcare products Regulatory Agency (MHRA) comments received on May 22nd, 2020.
Section 7.2.9 Safety Stopping Rules for the Entire Study	Clarification of study hold or stop by DMC decision	According to the Medicines and Healthcare products Regulatory Agency (MHRA) comments received on May 22nd, 2020.

The confidential information in this document is provided to you as an investigator, potential investigator or consultant for review by you, your staff and applicable Independent Ethics Committee or Review Board. It is understood that the information will not be disclosed to others without written authorization from Biophytis S.A.

Sections	Summary of Revisions	Rationale
Section 1.1 Synopsis<br>Section 1.3 Schedule of Activities<br>Section 3.3 Endpoints<br>Section 4.3.1 Timing of Assessments<br>Section 4.3.3.3 Population PK Study<br>Section 5 Study Population<br>Section 5.2 Exclusion Criteria<br>Section 6.3 Measures to Reduce Bias<br>Section 6.5 Concomitant Medications<br>Section 7.1 Assessment of Efficacy<br>Section 7.2 Safety Assessments<br>Section 8.2 Protocol Deviations<br>Section 9.1.5 Key Roles and Study Governance	Updates and corrections were made	Clarification and consistency throughout synopsis and body of the protocol

Amendment 2

Date: May 11, 2020

Sections	Summary of Revisions	Rationale
Section 1.1 Synopsis<br>Section 5.1 Inclusion Criteria	Clarification of the acceptable methods of contraception	According to the Federal agency for medicines and health products (FAMHP) comments received on May 7th, 2020.
Section 1.1 Synopsis<br>Section 5.1 Inclusion Criteria<br>Section 5.2 Exclusion Criteria	Updates and corrections to inclusion and exclusion criteria	Corrections for consistency of selection criteria in synopsis and report body

The confidential information in this document is provided to you as an investigator, potential investigator or consultant for review by you, your staff and applicable Independent Ethics Committee or Review Board. It is understood that the information will not be disclosed to others without written authorization from Biophytis S.A.

Sections	Summary of Revisions	Rationale
		According to the Federal agency for medicines and health products (FAMHP) comments received on May 7th, 2020.
Section 1.1 Synopsis<br>Section 3.3 Endpoints	Specific mention of safety endpoints included	According to the Federal agency for medicines and health products (FAMHP) comments received on May 7th, 2020.
Section 1.1 Synopsis<br>Section 1.3 Schedule of Activities<br>Section 3.3 Endpoints<br>Section 7.1.4 Pulse Oximetry and Fraction of Inspired Oxygen	Added RAS/MAS biomarkers and respiratory biomarkers (SpO_2/FiO_2) to endpoints	According to the Federal agency for medicines and health products (FAMHP) comments received on May 7th, 2020.
Section 2.4.3 The risk profile of BIO101<br>Section 6.3.1 Record of Randomization and Unblinding<br>Section 7.2 Safety Assessments	Correction and clarification	According to the Federal agency for medicines and health products (FAMHP) comments received on May 7th, 2020
Section 4.3.2 Pre-intervention Period<br>Section 9.1.1 General Informed Consent Process	Provision for the use of historic standard of care data collected on the same day as screening	Avoid the retaking of blood samples for the study if they were already taken per standard of care at the site

The confidential information in this document is provided to you as an investigator, potential investigator or consultant for review by you, your staff and applicable Independent Ethics Committee or Review Board. It is understood that the information will not be disclosed to others without written authorization from Biophytis S.A.

Sections	Summary of Revisions	Rationale
Section 1.1 Synopsis<br>Section 1.3 Schedule of Activities<br>Section 3.3 Endpoints<br>Section 4.3 Study Assessments<br>Section 4.4 Definition of End-of-study Participation<br>Section 5 Study Population<br>Section 6.3 Measures to Reduce Bias<br>Section 6.5 Concomitant Medications<br>Section 7.1 Assessment of Efficacy<br>Section 7.2 Safety Assessments<br>Section 7.3 Additional Assessments<br>Section 8.1 Study Cohorts<br>Section 8.4 Description of Statistical Methods<br>Section 9.1 Regulatory, Ethical, and Study Oversight Considerations	Updates and corrections were made	Clarification and consistency throughout synopsis and body of the protocol
Section 1.1 Synopsis<br>Section 4.3 Study Assessments<br>Section 4.4 Definition of End-of-study Participation<br>Section 6.2 Supply Procedures<br>Section 6.3 Measures to Reduce Bias<br>Section 7.1 Assessment of Efficacy	Correction of minor errors in language	Clarification

The confidential information in this document is provided to you as an investigator, potential investigator or consultant for review by you, your staff and applicable Independent Ethics Committee or Review Board. It is understood that the information will not be disclosed to others without written authorization from Biophytis S.A.

Sections	Summary of Revisions	Rationale
Section 7.2 Safety Assessments<br>Section 8.4 Description of Statistical Methods		

Amendment 1

Date: April 21, 2020

Sections	Summary of Revisions	Rationale
Section 1.1 Synopsis<br>Section 3.3 Endpoints	Updated endpoints for both part 1 and part 2	According to the French Ethic Committee (CPP-Ouest V) comments received on April, 7th, 2020 and the Agence Nationale de Sécurité du Médicament et des Produits de Santé (ANSM) comments received on April 17th, 2020.
Section 1.1 Synopsis<br>Section 1.3 Schedule of Activities<br>Section 3.3 Endpoints<br>Section 4.3 Study Assessments<br>Section 4.4 Definition of End-of-study Participation<br>Section 7.1 Assessment of Efficacy<br>Section 7.2 Safety Assessments	Adapted Schedule of Activities	Clarification
Section 1.1 Synopsis<br>Section 5.1 Inclusion Criteria<br>Section 5.2 Exclusion Criteria<br>Section 6.5 Concomitant Medications	Criteria were added and corrections were made	According to the Agence Nationale de Sécurité du Médicament et des Produits de Santé (ANSM) comments received on April 17th, 2020<br>Clarification

The confidential information in this document is provided to you as an investigator, potential investigator or consultant for review by you, your staff and applicable Independent Ethics Committee or Review Board. It is understood that the information will not be disclosed to others without written authorization from Biophytis S.A.

Sections	Summary of Revisions	Rationale
Section 1.1 Synopsis<br>Section 6.3 Measures to Reduce Bias	Modification of stratification factors for part 1, correction of stratification factors for part 2	According to the Agence Nationale de Sécurité du Médicament et des Produits de Santé (ANSM) comments received on April 17th, 2020<br>Clarification
Section 1.1 Synopsis<br>Section 1.2 Study Scheme<br>Section 4 Study Design<br>Section 8.3 Sample Size Justification<br>Section 8.4 Description of Statistical Methods	Reassessment of sample size calculation	Clarification
Section 6.2.2 Formulation, Appearance, Packaging, and Labeling<br>Section 6.2.4 Preparation	Clarification on packaging and labeling of IMP	Clarification
Section 7.2.9 Safety Stopping Rules for Entire Study	Stopping rules for the entire study were clarified.	According to the Agence Nationale de Sécurité du Médicament et des Produits de Santé (ANSM) comments received on April 17th, 2020
Section 1.1 Synopsis<br>Section 2.2 Bio101<br>Section 3 Objectives and Endpoints<br>Section 4.4 Definition of End-of-study Participation<br>Section 5 Study Population<br>Section 6.2 Supply Procedures<br>Section 7.2 Safety Assessments	Addition of clarifications and correction of errors	Clarification

The confidential information in this document is provided to you as an investigator, potential investigator or consultant for review by you, your staff and applicable Independent Ethics Committee or Review Board. It is understood that the information will not be disclosed to others without written authorization from Biophytis S.A.

Sections	Summary of Revisions	Rationale
Section 7.3 Additional Assessments<br>Section 9.1 Regulatory, Ethical, and Study Oversight Considerations		

The confidential information in this document is provided to you as an investigator, potential investigator or consultant for review by you, your staff and applicable Independent Ethics Committee or Review Board. It is understood that the information will not be disclosed to others without written authorization from Biophytis S.A.

Sponsor Signature Page

**Protocol Title**	Adaptive design phase 2 to 3, randomized, double- blind, multicenter, to evaluate the safety, efficacy, pharmacokinetics and pharmacodynamics of BIO101 in the prevention of the respiratory deterioration in hospitalized patients with COVID-19 pneumonia (severe stage)
**Protocol Number**	BIO101-CL05
**Sponsor**	Biophytis S.A.

Waly Dioh, PhD, Chief Operations Officer	Date (day/month/year)
Sam Agus, MD, Chief Medical Officer	Date (day/month/year)

e-signature can be found at the end document.

The confidential information in this document is provided to you as an investigator, potential investigator or consultant for review by you, your staff and applicable Independent Ethics Committee or Review Board. It is understood that the information will not be disclosed to others without written authorization from Biophytis S.A.

Investigator Signature Page

**Protocol Title**	Adaptive design phase 2 to 3, randomized, double- blind, multicenter, to evaluate the safety, efficacy, pharmacokinetics and pharmacodynamics of BIO101 in the prevention of the respiratory deterioration in hospitalized patients with COVID-19 pneumonia (severe stage)
**Protocol Number**	BIO101-CL05
**Sponsor**	Biophytis S.A.

Investigator:

Date (day/month/year)

The confidential information in this document is provided to you as an investigator, potential investigator or consultant for review by you, your staff and applicable Independent Ethics Committee or Review Board. It is understood that the information will not be disclosed to others without written authorization from Biophytis S.A.

Contents

Summary of Version Changes: 2
Amendment 9 2
Amendment 8 2
Amendment 7 3
Amendment 6 5
Amendment 5 5
Amendment 4 6
Amendment 3 9
Amendment 2 11
Amendment 1 14
Sponsor Signature Page 17
Investigator Signature Page 18
Contents 19
1 General Information 24
1.1 Synopsis 24
1.2 Study Scheme 30
1.3 Schedule of Activities 31
2 Introduction 34
2.1 Background 34
2.2 Bio101 35
2.2.1 Non-clinical Efficacy 35
2.2.2 Non-clinical Safety 35
2.2.3 Clinical Studies 36
2.3 Scientific Rationale 39
2.4 Benefit / Risk Assessment 41
2.4.1 The Unmet Need 41
2.4.2 The Potential Role of BIO101 in the Treatment of COVID-19 42
2.4.3 The Risk Profile of BIO101 42
2.4.4 Risks Associated with the COVID 19 Epidemic 42

The confidential information in this document is provided to you as an investigator, potential investigator or consultant for review by you, your staff and applicable Independent Ethics Committee or Review Board. It is understood that the information will not be disclosed to others without written authorization from Biophytis S.A.

2.4.5 Conclusion 43
3 Objectives and Endpoints 43
3.1 Part 1 43
3.2 Part 2 43
3.3 Endpoints 43
3.3.1 Proportion and Time to Events 43
3.3.2 Assessment of Participant General and Respiratory Status 44
3.3.3 Biomarkers 44
3.3.4 Pharmacokinetics of BIO101 and its Metabolites 46
3.3.5 Assignment of Study Endpoints 46
4 Study Design 50
4.1 Rationale for Study Design 51
4.1.1 Justification of Selection of Study Population 52
4.1.2 Justification for selection of endpoints 52
4.1.3 Justification of randomization scheme 53
4.2 Justification for Dose 53
4.3 Study Assessments 53
4.3.1 Timing of Assessments 54
4.3.2 Pre-intervention Period 54
4.3.3 On-intervention Period 55
4.3.4 Post-intervention Period 56
4.4 Definition of End-of-study Intervention 56
5 Study Population 56
5.1 Inclusion Criteria 57
5.2 Exclusion Criteria 58
5.3 Withdrawal of Participants During the Study 58
6 Study Medication 59
6.1 Dosing and Administration 59
6.1.1 Administration of First Dose 59
6.2 Supply Procedures 60

The confidential information in this document is provided to you as an investigator, potential investigator or consultant for review by you, your staff and applicable Independent Ethics Committee or Review Board. It is understood that the information will not be disclosed to others without written authorization from Biophytis S.A.

6.2.1 Accountability ... 60
6.2.2 Formulation, Appearance, Packaging, and Labeling ... 60
6.2.3 Storage and Stability ... 61
6.2.4 Preparation ... 61
6.3 Measures to Reduce Bias ... 61
6.3.1 Record of Randomization and Unblinding ... 61
6.3.2 Dissemination of Results ... 62
6.3.3 Potential for Operational Bias ... 62
6.4 Compliance ... 62
6.5 Concomitant Medications ... 63
6.5.1 Disallowed Medications ... 63
6.5.2 Allowed Medications ... 63
6.6 Rescue Medications ... 63
7 Study Assessments ... 63
7.1 Assessment of Efficacy ... 63
7.1.1 Record of Events ... 63
7.1.2 Status Scales ... 64
7.1.3 Biomarkers ... 64
7.1.4 Pulse Oximetry and Fraction of Inspired Oxygen ... 64
7.1.5 Timing of Efficacy Assessments ... 65
7.2 Safety Assessments ... 65
7.2.1 Physical Examination ... 65
7.2.2 Safety Labs ... 65
7.2.3 Electrocardiogram (ECG) ... 67
7.2.4 Safety Reporting ... 67
7.2.5 Unanticipated Events Related to Study Procedures, but not to Study Medication ... 70
7.2.6 Follow-up on Adverse Events ... 71
7.2.7 Adverse Events Reporting ... 71
7.2.8 Safety Stopping Rules ... 72
7.2.9 Safety Stopping Rules for Entire Study ... 74

The confidential information in this document is provided to you as an investigator, potential investigator or consultant for review by you, your staff and applicable Independent Ethics Committee or Review Board. It is understood that the information will not be disclosed to others without written authorization from Biophytis S.A.

7.3 Additional Assessments 75
7.3.1 Medical History 75
7.3.2 Concomitant Medications 75
7.3.3 Anthropometry 75
7.3.4 Pregnancy Testing 75
7.3.5 Genetic Assessments 75
7.3.6 Population PK 75
8 Statistics 75
8.1 Study Cohorts 75
8.1.1 Intent to Treat (ITT) 75
8.1.2 Safety Population (SafP) 75
8.1.3 Per-Protocol (PP) 76
8.2 Protocol Deviations 76
8.2.1 Major Protocol Deviations 76
8.3 Sample Size Justification 76
8.4 Description of Statistical Methods 77
8.4.1 General Analytical Considerations 77
8.4.2 Method of Assigning Participants to Treatment Groups 78
8.4.3 Analysis of Demographic and Other Baseline Characteristics 78
8.4.4 General Analytical Considerations for Efficacy and Safety Analyses 78
8.4.5 Analysis of Primary and Key Secondary Endpoints and Sequential Testing 79
8.4.6 Handling of Missing Data 80
8.4.7 Sample Size Re-assessment at Interim Analysis 80
8.4.8 Safety Data 81
9 Supporting Documentation and Operational Procedures 82
9.1 Regulatory, Ethical, and Study Oversight Considerations 82
9.1.1 General Informed Consent Process 82
9.1.2 Study Discontinuation and Site Closure 83
9.1.3 Confidentiality and Privacy 84
9.1.4 Future Use of Stored Specimens and Data 85

The confidential information in this document is provided to you as an investigator, potential investigator or consultant for review by you, your staff and applicable Independent Ethics Committee or Review Board. It is understood that the information will not be disclosed to others without written authorization from Biophytis S.A.

9.1.5 Key Roles and Study Governance ... 85
9.1.6 Safety oversight ... 86
9.1.7 Clinical Monitoring ... 86
9.1.8 Data Quality Assurance and Record Keeping ... 86
9.1.9 Source Documents ... 87
9.1.10 Study Records Retention ... 88
9.1.11 Protocol Deviations ... 88
9.1.12 Publication and Data Sharing Policy ... 89
9.1.13 Conflict of Interest Policy ... 89
10 List of Tables ... 90
11 List of Figures ... 90
12 Abbreviations and Definitions ... 91
13 References ... 95

1 General Information

1.1 Synopsis

<table>
<tr><td>Title</td><td>Adaptive design phase 2 to 3, randomized, double- blind, multicenter, to evaluate the safety, efficacy, pharmacokinetics and pharmacodynamics of BIO101 in the prevention of the respiratory deterioration in hospitalized patients with COVID-19 pneumonia (severe stage)</td></tr>
<tr><td>Identifier</td><td>BIO101_CL05</td></tr>
<tr><td>Sponsor</td><td>Biophytis SA</td></tr>
<tr><td>Method</td><td>Randomized, double-blind, placebo-controlled, group sequential and adaptive</td></tr>
<tr><td>Description and sample size</td><td>This will be a double-blind, placebo-controlled, group sequential and adaptive study, which will be conducted in 2 parts:
<table>
<tr><th>Part</th><th>Goal</th><th>Number of participants</th></tr>
<tr><td>1</td><td>Obtain indication of activity of BIO101, about the effect of BIO101 in preventing further respiratory deterioration</td><td>50
1:1 randomization</td></tr>
<tr><td rowspan="2">2</td><td>Re-assessment of the sample size for part 2</td><td>155 (an addition of 105 participants)
1:1 randomization</td></tr>
<tr><td>Confirmation of the effect of BIO101 in preventing further respiratory deterioration and obtaining a conditional approval</td><td>310, potentially increased by 50% (up to 465, based on interim analysis 2)
1:1 randomization</td></tr>
</table>
All participants will undergo the same assessments, during a period of 28 days, which will allow pooling of the data from the 2 parts, for the primary analysis of the full study data. Participants will be identified according to the part during which they are recruited.

During the data collection in part 1, an unblinded interim analyses will be conducted by an independent Data Monitoring Committee (DMC), to assess safety signals and begin the recruitment to part 2. In addition, an analysis of the study</td></tr>
</table>

The confidential information in this document is provided to you as an investigator, potential investigator or consultant for review by you, your staff and applicable Independent Ethics Committee or Review Board. It is understood that the information will not be disclosed to others without written authorization from Biophytis S.A.

	end-points will be conducted, to potentially obtain and indication for the activity of BIO101, in the study population.<br><br>During part 2, an additional unblinded interim analysis will take place, to re-assess the sample size for part 2.
**Target population**	Age 45 and older<br><br>Confirmed infection with SARS-CoV-2<br><br>With signs of pneumonia at severe grade (i.e. with signs of pneumonia at severe grade or mild ARDS) but not invasively ventilated
**Inclusion criteria**	1. Age: 45 and older.<br>2. A confirmed diagnosis of COVID-19 infection, within the last 14 days, prior to randomization, as determined by PCR or other approved commercial or public health assay, in a specimen as specified by the test used**.**<br>3. Hospitalized, in observation or planned to be hospitalized due to COVID-19 infection symptoms with anticipated hospitalization duration >=3 days<br>4. With evidence of pneumonia based on **all** of the following:<br>a. Clinical findings on a physical examination<br>b. Respiratory symptoms developed within the past 7 days<br>5. With evidence of respiratory decompensation that started not more than 4 days before start of study medication and present at screening, meeting **one** of the following criteria, as assessed by healthcare staff:<br>a. Tachypnea: ≥25 breaths per minute<br>b. Arterial oxygen saturation ≤92%<br>c. A special note should be made if there is suspicion of COVID-19-related myocarditis or pericarditis, as the presence of these is a stratification criterion<br>6. Without a significant deterioration in liver function tests:<br>a. ALT and AST ≤ 5x upper limit of normal (ULN)<br>b. Gamma-glutamyl transferase (GGT) ≤ 5x ULN<br>c. Total bilirubin ≤ 5×ULN<br>7. Willing to participate and able to sign an informed consent form (ICF). Or, when relevant, a legally authorized representative (LAR) might sign the ICF on behalf of the study participant<br>8. Female participants should be:<br>at least 5 years post-menopausal (i.e., persistent amenorrhea 5 years in the absence of an alternative medical cause) or surgically sterile;<br>OR<br>a. Have a negative urine pregnancy test at screening<br>b. Be willing to use a contraceptive method as outlined in inclusion criterion 9 from screening to 30 days after last dose.

The confidential information in this document is provided to you as an investigator, potential investigator or consultant for review by you, your staff and applicable Independent Ethics Committee or Review Board. It is understood that the information will not be disclosed to others without written authorization from Biophytis S.A.

	9. Male participants who are sexually active with a female partner must agree to the use of an effective method of birth control throughout the study and until 3 months after the last administration of investigational product;<br>Note: medically acceptable methods of contraception that may be used by the participant and/or partner include combined oral contraceptive, contraceptive vaginal ring, contraceptive injection, intrauterine device, etonogestrel implant, each supplemented with a condom, as well as sterilization and vasectomy.<br>10. Female participant who are lactating must agree not to breastfeed during the study and up to 14 days after the intervention.<br>11. Male participants must agree not to donate sperm for the purpose of reproduction throughout the study and until 3 months after the last administration of investigational product;<br>12. For France only: Being affiliated with a European Social Security.
**Exclusion criteria**	1. Not needing or not willing to remain in a healthcare facility during the entire study medication (i.e. while receiving study medication)<br>2. Moribund condition (death likely in days) or not expected to survive for >7 days – due to other and non-COVID-19 related conditions<br>3. Participant on invasive mechanical ventilation via an endotracheal tube, or extracorporeal membrane oxygenation (ECMO), or high-flow Oxygen*<br>4. Participant not able to take medications by mouth (as capsules or as a powder, mixed in water).<br>5. Disallowed concomitant medication:<br>a. Consumption of any herbal products containing 20-hydroxyecdysone and derived from *Leuzea carthamoides*; *Cyanotis vaga* or *Cyanotis arachnoidea* is not allowed (e.g. performance enhancing agents)<br>6. Any known hypersensitivity to any of the ingredients, or excipients of the study medication, BIO101<br>7. Renal disease requiring dialysis, or known renal insufficiency (eGFR≤30 mL/min/1.73 m^2, based on Cockcroft & Gault formula)<br>8. In France:<br>o Non-affiliation to compulsory French social security scheme (beneficiary or right-holder)<br>o Being under tutelage or legal guardianship<br><br>* High-flow oxygen is defined as delivery of oxygen at a flow of ≥16 L/min.
**Treatment arms and presentation**	1. BIO101, 350mg b.i.d.<br>2. Placebo<br><br>Presentation: capsule for oral administration.
**Duration**	28 days

The confidential information in this document is provided to you as an investigator, potential investigator or consultant for review by you, your staff and applicable Independent Ethics Committee or Review Board. It is understood that the information will not be disclosed to others without written authorization from Biophytis S.A.

**Endpoints**	**For end of part-1 interim analysis:**<br><br>***For safety analysis intended to facilitate the decision to begin part 2, time frame – up to 28 days:***<br><br>*Safety and tolerability to BIO101:*<br>• SUSARs, SAEs, AESIs, AEs<br>• Vital signs<br>• Safety labs (including testicular biomarkers)<br>• ECGs<br><br>***For interim analysis intended to obtain indication of activity of BIO101, time frame – up to 28 days:***<br><br>*Primary:*<br>• Proportion of participants with 'negative' events, of either of the following:<br>o All-cause mortality<br>o Respiratory failure, defined as any of the following:<br>▪ Requiring mechanical ventilation (including cases that will not be intubated due to resource restrictions and triage)<br>▪ Requiring ECMO<br>▪ Requiring high-flow oxygen<br><br>*Secondary:*<br>• SpO_2/FiO_2<br>• Inflammatory markers including:<br>o IL 6<br>o TNFα<br>o D-dimer<br>• RAS / MAS biomarkers:<br>o Angiotensin 2<br>o Angiotensin-converting enzyme (ACE) levels<br><br>**For part-2 sample size interim analysis:**<br><br>*For sample size re-assessment for part 2, time frame – up to 28 days:*<br>• Proportion of participants with 'negative' events, of either of the following:<br>o All-cause mortality<br>o Respiratory failure, defined as any of the following:<br>▪ Requiring mechanical ventilation (including cases that will not be intubated due to resource restrictions and triage)<br>▪ Requiring ECMO<br>▪ Requiring high-flow oxygen

The confidential information in this document is provided to you as an investigator, potential investigator or consultant for review by you, your staff and applicable Independent Ethics Committee or Review Board. It is understood that the information will not be disclosed to others without written authorization from Biophytis S.A.

	**For the final analysis:**<br>*Primary, time frame – up to 28 days:*<br>• Proportion of participants with 'negative' events, of either of the following:<br>o All-cause mortality<br>o Respiratory failure, defined as any of the following:<br>▪ Requiring mechanical ventilation (including cases that will not be intubated due to resource restrictions and triage)<br>▪ Requiring ECMO<br>▪ Requiring high-flow oxygen<br>*Key secondary*:<br>• Proportion of participants with events of respiratory failure, defined as any of the following:<br>o Requiring mechanical ventilation (including cases that will not be intubated due to resource restrictions and triage)<br>o Requiring ECMO<br>o Requiring high-flow oxygen<br>• Proportion of participants with 'positive' events:<br>o official discharge from hospital care by the department due to improvement in participant condition (self-discharge by participant is not considered a positive event)<br>• Proportion of participants with events of all-cause mortality<br>*Additional secondary endpoints*:<br>• SpO_2/FiO_2<br>• Oxygen saturation in arterial blood, measured by pulse-oximetry (SpO_2)<br>• Time to events, of either of the following:<br>o All-cause mortality<br>o Respiratory failure, defined as any of the following:<br>▪ Requiring mechanical ventilation (including cases that will not be intubated due to resource restrictions and triage)<br>▪ Requiring ECMO<br>▪ Requiring high-flow oxygen<br>• Time to event: official discharge from hospital care due to improvement<br>• The National Early Warning Score 2 (NewS2)<br>• Proportion of participants with CPAP/BiPAP events, defined as requiring CPAP/BiPAP in participants entering the study on low flow oxygen)<br>• For participants who experienced a 'positive' event: proportion of participants with sustained positive outcome (to assess durability of effect after those participants discontinued study medication)<br>• Population-PK study (pop-PK)<br>*Exploratory measures:*<br>• Viral load in nasal/nasopharyngeal swabs

The confidential information in this document is provided to you as an investigator, potential investigator or consultant for review by you, your staff and applicable Independent Ethics Committee or Review Board. It is understood that the information will not be disclosed to others without written authorization from Biophytis S.A.

	• Arterial blood gas analysis (ABG)<br>• PaO_2/FiO_2<br>• RAS / MAS biomarkers:<br>o Angiotensin 1-7 (Ang 1-7)<br>o Angiotensin 1-5 (Ang 1-5)<br>o Angiotensin 2<br>o Renin<br>o Aldosterone<br>o Angiotensin-converting enzyme (ACE) levels<br>• Inflammatory markers including:<br>o IL-2, 6 and 10<br>o TNFα<br>o Troponin (I and T)<br>o LDH<br>o D-dimer<br>o CRP plasma levels<br>o Ferritin<br>• Skeletal muscle proteins including CK-MM, CK-MB; Myoglobin, CK macro type I, CK macro type II<br>• PIIINP<br>• bone specific alkaline phosphatase<br>• Acute Physiology and Chronic Health Evaluation (APACHE) II score<br><br>*Safety and tolerability to BIO101*:<br><br>• SUSARs, SAEs, AESIs, AEs<br>• Vital signs<br>• Safety labs<br>• ECGs

The confidential information in this document is provided to you as an investigator, potential investigator or consultant for review by you, your staff and applicable Independent Ethics Committee or Review Board. It is understood that the information will not be disclosed to others without written authorization from Biophytis S.A.

1.2 Study Scheme

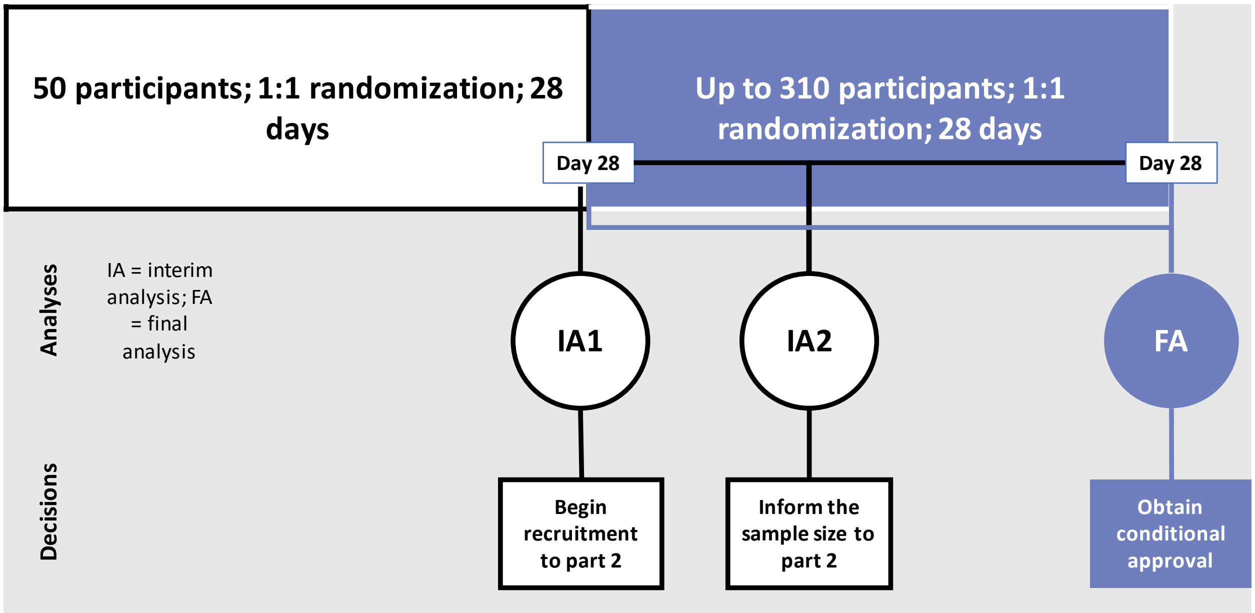

Figure 1: COVA Study Scheme

1.3 Schedule of Activities

Visit	Screening	Baseline[i]	A1	A2	A3	End of treatment[ii]	Follow-up 1	Follow-up 2
**Study period**	**Pre-intervention**		**On-intervention**				**Post-intervention**	
**Study day (one day only, in each given window)[iii]**	**-1 to 0**	**0**	**2 to 4**	**6 to 8**	**12 to 16**	**At reaching study endpoint or Day 28**	**14 (± 2) days after the last on-intervention visit**	**60 (± 4) days after the last on-intervention visit**
**Location**	**Site**	**Site**	**Site**	**Site**	**Site**	**Site**	**Phone**	**Phone**
Informed consent[iv]	+							
Medical history	+							
Concomitant medications	+	+	+	+	+	+	+	+
Full physical examination	+							
Short physical examination[v]		+	+	+	+	+		
Oxygen saturation (Pulse oximetry)	+	+	+	+	+	+		
Record FiO_2 and oxygen delivery method[vi]	+	+	+	+	+	+	+[vii]	+[viii]
Vital signs	+	+	+	+	+	+		

---

[i] All the baseline activities are done before the study medication administration (except for the post-administration of the blood draws of the pop-PK study)

[ii] Refers to reaching a 'negative' or a 'positive' endpoint (regardless on study day)

[iii] When a range is given, all activities for the range have to be done on the same day, within this range

[iv] Study activities cannot start before the participant has signed an informed consent form!

[v] Heart, lung, abdomen and a brief neuro exam only

[vi] The oxygen delivery method (e.g., simple facial mask; partial rebreather mask; nasal cannula; C-PAP machine; …) is to be recorded. If FiO_2 cannot be read directly from the device, the flow rate (in L/min) and % of oxygen the system delivers (e.g., 100%) should be recorded instead.

[vii] During the phone call, information will be collected on whether the participant requires or has required oxygen supplementation (including duration) since the last on-intervention visit.

[viii] During the phone call, information will be collected on whether the participant requires or has required oxygen supplementation (including duration) since the last on-intervention visit.

The confidential information in this document is provided to you as an investigator, potential investigator or consultant for review by you, your staff and applicable Independent Ethics Committee or Review Board. It is understood that the information will not be disclosed to others without written authorization from Biophytis S.A.

Visit	Screening	Baseline[i]	A1	A2	A3	End of treatment[ii]	Follow-up 1	Follow-up 2
**Study period**	**Pre-intervention**		**On-intervention**				**Post-intervention**	
**Study day (one day only, in each given window)[iii]**	**-1 to 0**	**0**	**2 to 4**	**6 to 8**	**12 to 16**	**At reaching study endpoint or Day 28**	**14 (± 2) days after the last on-intervention visit**	**60 (± 4) days after the last on-intervention visit**
**Location**	**Site**	**Site**	**Site**	**Site**	**Site**	**Site**	**Phone**	**Phone**
Anthropometry	+							
Electrocardiography		+		+		+		
Record of a study event ('negative' or 'positive')						+	+[ix]	+[x]
NewS2		+	+	+	+	+		
APACHE II (Optional)		+	+		+	+		
ABG (Optional)		+	+		+	+		
Biomarkers (RAS/MAS)		+				+		
Biomarkers (Muscle)		+		+		+		
Biomarkers (Inflammatory)		+	+	+	+	+		
Biomarkers (PIIINP, myoglobin, bone specific alkaline phosphatase)		+	+	+	+	+		
Viral load (nasal/nasopharyngeal sample)		+		+		+		
ACE-2 genotype (optional consent)		+						
Adverse events	+	+	+	+	+	+	+	+
Biochemistry / hematology	+		+	+	+	+		

[ix] During the phone call, information will be collected on whether the participant has experienced any positive or negative events since the last on-intervention visit.
[x] During the phone call, information will be collected on whether the participant has experienced any positive or negative events since the last on-intervention visit.

The confidential information in this document is provided to you as an investigator, potential investigator or consultant for review by you, your staff and applicable Independent Ethics Committee or Review Board. It is understood that the information will not be disclosed to others without written authorization from Biophytis S.A.

**Visit**	**Screening**	**Baseline**[i]	**A1**	**A2**	**A3**	**End of treatment**[ii]	**Follow-up 1**	**Follow-up 2**
**Study period**	**Pre-intervention**		**On-intervention**				**Post-intervention**	
**Study day (one day only, in each given window)**[iii]	**-1 to 0**	**0**	**2 to 4**	**6 to 8**	**12 to 16**	**At reaching study endpoint or Day 28**	**14 (± 2) days after the last on-intervention visit**	**60 (± 4) days after the last on-intervention visit**
**Location**	**Site**	**Site**	**Site**	**Site**	**Site**	**Site**	**Phone**	**Phone**
Urinalysis		+	+	+	+	+		
Coagulation tests		+		+		+		
Testicular biomarkers[xi]		+	+	+	+	+		
Urine pregnancy test (β-HCG)	+							
Randomization		+						
Study medication administration		+	+	+	+	+		
Pop-PK blood draws[xii]		+		+				
Participant location[xiii]							+	+

[xi] for male participants only (FSH, LH, Testosterone and Inhibin-B)

[xii] 3 blood draws, predose and 4 (±10%) and 24 hours (±10%) postdose, before the morning dose of subsequent day – see more information in section 3.3.5 and 4.3.3.3

[xiii] During the phone call, information will be collected regarding the location of the participant (and duration of stay) since the last on-intervention visit (home, intensive care unit, regular hospital ward).

The confidential information in this document is provided to you as an investigator, potential investigator or consultant for review by you, your staff and applicable Independent Ethics Committee or Review Board. It is understood that the information will not be disclosed to others without written authorization from Biophytis S.A.

2 Introduction

2.1 Background

Coronavirus Disease known as COVID-19, and caused by the SARS coronavirus 2 (SARS-CoV-2) is a pandemic, with approximately daily 50,000 newly identified cases and nearly 3,000 fatalities.

While there are a few cases of asymptomatic carriers, COVID-19 is usually symptomatic. This virus has an incubation period of 5-12 days and an average reproductive rate of 3.28[1]. The mortality rate is approximately 2.3%, with about 5% in critical conditions (i.e. require ventilatory support and critical care), 14% considered as severe (i.e. with respiratory decompensation) and the rest as moderate (with evidence of pneumonia) or mild (only upper respiratory or mild respiratory signs)[2]. The median time from onset of symptoms to mortality is 18.8 days and to recovery is 22.6 days[3]. The median time from onset to respiratory failure is 12 days[4]. While the key effort to combat the spread of COVID-19 is to develop a vaccine, it is not expected that one will be available in the immediate future. The current estimation is that it will take 18 months for the first vaccine to be available for use in the general population.

Besides vaccination studies, other paradigms are also being tested. The following approaches are currently under investigation:

Table 1: Summary of medications that are being tested for COVID-19

**Mechanism of Action**	**Examples**
Antiviral medications	RNA-dependent-RNA polymerase inhibitors: e.g. some anti-HIV medications, hydroxychloroquine<br>Protease inhibitors: e.g. some anti-HIV and anti-HCV medications<br>Ribosome assembly inhibitors: e.g. azithromycin<br>Antibodies against the SARA-CoV-2 SPIKE protein<br>Convalescent plasma
Immunomodulation	Mesenchymal stem-cells (MSC) and MAS-derived exosomes<br>Anti-IL6-MAbs<br>TLR-2/6/7 modulators<br>CD24-IGF1 Fc domain recombinant construct<br>Synthetic VIP (also improves pulmonary clearance via bronchodilation and increased mucus secretion)<br>Dexamethasone
RAS modulators	Recombinant ACE2<br>Angiotensin receptor blockers (e.g. losartan)

The confidential information in this document is provided to you as an investigator, potential investigator or consultant for review by you, your staff and applicable Independent Ethics Committee or Review Board. It is understood that the information will not be disclosed to others without written authorization from Biophytis S.A.

Mechanism of Action	Examples
	Angiotensin 1-7
Others	e.g. herbs that are used in traditional Chinese medicine

2.2 Bio101

The active ingredient for BIO101 is 20-hydroxyecdysone or 20E, purified to 97% from the plant of the Cyanotis species (including *Cyanotis vaga Lour. (Shultes)* or *Cyanotis arachnoidea C.B. Clarke)*.

This investigational product is formulated as 175 mg oral capsules, contained in elderly friendly weekly and monthly boxes.

2.2.1 Non-clinical Efficacy

No studies were performed *in vitro* or *in vivo* in models that relate to the COVID-19 infection. Non-clinical data with study medication in other models are described in section 2.3.

2.2.2 Non-clinical Safety

Toxicology studies with BIO101 were performed with a 4 and 26 weeks of oral administration in rodents and 4, 26 and 39 weeks in canines. In these studies, safety pharmacology parameters were investigated, including cardiovascular functions in dogs, and central nervous and respiratory systems in rats. BIO101 showed no mortality and no treatment-related changes in body weight, no effect on arterial blood pressure, heart rate, body temperature, or electrocardiogram (ECG) parameters in conscious male beagle dogs. Single oral administration of BIO101 had no effect on central nervous system activities or respiratory functions at 100, 300, and 1,000 mg/kg in rats.

In the 4-week study in rats, a NOAEL (No Observed Adverse Effect Level) for BIO101 of 1,000 mg/kg/day was established, based on the absence of histopathological and clinical adverse effects at the tested doses of 100, 300 and 1,000 mg/kg/day.

In the 4-week study in beagle dogs, the NOAEL was established at 1,500 mg/kg/day based on the absence of obvious signs of toxicity. The doses of 150, 500 and 1,500 mg/kg were tested.

In the 26-week toxicology study in rats with oral doses of 100, 300 and 1,000 mg/kg, no treatment-related deaths were observed and no negative clinical signs could be linked to the test item. This study was completed in January 2017. In females only, higher cholesterol and triglycerides levels than in controls were observed starting at 1,000 mg/kg. At the histopathological level in males, tubular degeneration/atrophy were observed at the highest dose (1,000 mg/kg/day). In females only, hepatocellular hypertrophy, was observed in the liver at the highest dose only. Decrease in corpora lutea development and increase in follicular cysts in the ovaries were observed at the doses of 300 and 1,000 mg/kg/day. Increase in keratinized epithelium and squamous cell hyperplasia in the vagina were observed in 300 and 1,000 mg/kg/day. These last histopathological findings in females were also present in the placebo group and therefore they were considered not significant. Overall, the NOAEL in rats was established at 300 mg/kg/day in males and 1000 mg/kg/day in females.

The confidential information in this document is provided to you as an investigator, potential investigator or consultant for review by you, your staff and applicable Independent Ethics Committee or Review Board. It is understood that the information will not be disclosed to others without written authorization from Biophytis S.A.

The 26-week toxicology study in beagle dogs was completed in March 2017. At 150, 500 and 1,500 mg/kg, no product related mortality and no marked clinical signs were observed in male and female beagle dogs. Body weight and food consumption were unaffected. The bioanalytical results showed that BIO101 induced a higher direct bilirubin values at week 27 when administered at 500 mg/kg/day, and a higher direct bilirubin and alkaline phosphatase activity at week 27 when administered at 1,500 mg/kg/day without associated histopathological changes. The histopathology examination also revealed a mild or minimal testicular germinal epithelial degeneration in half of males given BIO101 at 1,500 mg/kg/day. However, the two males with mild or minimal testicular degeneration also had the highest AUC_{last} (20,844 and 12,462 ng/ml*h) compared to AUC_{last} (7,623 and 5,192 ng/ml*h) for the two other males from the same group. There was no such change in males given BIO101 at 150 mg/kg/day and 500 mg/kg/day. In conclusion, based on the testicular germinal epithelial degeneration in half of males given BIO101 at 1,500 mg/kg, the NOAEL of BIO101 administered orally for 26 weeks in the male and in the female beagle dogs was 500 mg/kg in males and 1500 mg/kg in females.

On this basis, the 700 mg dose was set as the maximum daily dose (with a safety ratio of 5 considering the administered doses or a margin of 7.5 considering plasma exposure) for a long-term (26-week) administration in human participants in phase 2 studies.

In the chronic 39-week toxicity study in dogs, a slight increase in pulmonary inflammatory changes, in particular in females, was seen at 500 and 1000 mg/kg. The pulmonary changes, likely related to regurgitation, were fully reversible after a 4-week treatment-free period and were considered non-adverse. Although changes in testicular germinal epithelial degeneration were seen at the 1,500 mg/kg dose in males, they were not seen at 1,000 mg/kg in dogs in the 39-week study and the NOAEL for both males and females was 1,000 mg/kg.

Based on the non-clinical data, it was recommended not to include participants with evidence of an active biliary disease in studies with a lengthy exposure, and to put in place stopping rules based on liver function tests. This will be further discussed in section 2.4.

2.2.3 Clinical Studies

2.2.3.1 Phase 1 Study

The safety and pharmacokinetics of BIO101 were evaluated in the SARA-PK trial, a phase 1, double-blind, placebo-controlled, randomized clinical trial consisted of both a single oral administration in both young and elderly healthy volunteers and a 14-day multiple oral administration in elderly healthy volunteers only. The overarching objective of SARA-PK was to establish the range of oral doses suitable to be administered and evaluated in phase 2 clinical trials. This two- part clinical study started on August 2016 and was completed on December 2016[27].

In the Single Administration Dose (SAD) part, 2 cohorts of young healthy volunteers (18-55 years) in fasting state were dosed at 100, 350, 700 and 1,400 mg/day and one cohort of elderly healthy volunteers (65-85 years) was tested at 1,400 mg/day. Food effect was evaluated at 700 mg by comparing safety and pharmacokinetics in fed and fasted administration in the same cohort of young healthy volunteers (18-55 years). Age effect was evaluated at 1,400 mg/day by comparing safety and pharmacokinetics in the young healthy volunteer group to the elderly healthy volunteer group (65-85 years).

No serious adverse events (SAEs) were observed up to 1,400 mg/day in young or elderly cohorts. Adverse events (AEs) were mild and were reported at the doses of 350 mg and 1,400 mg in the young study

The confidential information in this document is provided to you as an investigator, potential investigator or consultant for review by you, your staff and applicable Independent Ethics Committee or Review Board. It is understood that the information will not be disclosed to others without written authorization from Biophytis S.A.

participants and at 1,400 mg for the elderly study participants. Most frequently reported AEs were gastrointestinal disorders, nervous system disorders (headache) and musculoskeletal and connective tissue disorders. No meaningful treatment-emergent laboratory, ECG or vital sign abnormalities were reported.

After a single administration in fasting conditions, BIO101 was rapidly absorbed with a median time to C_{max} between 2 and 3.5 hours. BIO101 plasma concentrations increased less than dose proportionally between 100 and 700 mg, and dose proportionally between 700 and 1,400 mg. After a single oral dose of 100, 350, 700 or 1,400 mg was administered in the fasting state, C_{max} of 141, 317, 399 and 710 ng/mL were observed respectively. The AUC was 797, 1,946, 2,658 and 4,283 ng*h/mL respectively. The mean half-life was short; between 2.4 and 4.9 hours.

No age effect was observed on the pharmacokinetic profile of BIO101; the comparison of young healthy volunteers and elderly healthy volunteers at 1,400 mg/day in SAD did not show a meaningful difference in the C_{max} (710 vs 552 ng/mL) or in the AUC (4,283 vs 3,630 ng*h/mL).

In addition, no food effect was observed on the pharmacokinetic profile of BIO101; the comparison of healthy young volunteers dosed at 700 mg in fasted or fed conditions did not show a meaningful difference in the C_{max} (399 ng/mL vs to 505 ng/mL) or in the AUC (2658 vs 3294 ng*h/mL). It was therefore decided to use the fed condition in the Multiple Ascending Doses (MAD) part.

Overall, BIO101 was well tolerated in doses ranging from 100 to 1,400 mg in a single oral administration. C_{max} observed at 350 mg and 700 mg/day (399 and 505 ng/mL respectively) corresponds to the pharmacologically active concentrations of BIO101 for myostatin inhibition and myotubes hypertrophy in rodents and human myocytes (140 and 480 ng/mL, respectively). Both doses of 350 mg and 700 mg were therefore retained to be tested in the 14-day MAD part under fed condition.

Based on the relatively short half-life observed under single administration conditions, it was decided to compare, sequentially, 350 mg once a day and 350 mg b.i.d. Subsequently, based on the good safety profile observed, a higher dose was also tested at 450 mg b.i.d.

In the MAD part, three cohorts of elderly healthy volunteers (65-85 years) were administered 350 mg q.d., 350 mg b.i.d. and 450 mg b.i.d. during a 14-day period. No SAEs were observed. Reported AEs were either mild or moderate (the latest limited to 450 mg b.i.d.). The highest number of study participants with treatment related AEs was reported in the cohort of 450 mg b.i.d., in 5 study participants. Treatment related AEs were reported in at most 1 participant each in the 350 mg q.d. and the 350 mg b.i.d. cohorts. None of the observed treatment-emergent laboratory, ECG or vital sign abnormalities were considered clinically significant and none were reported as AEs. Overall, 350 q.d. and 350 b.i.d. were equally safe.

Administration of 350 mg q.d. generated a C_{max} of 346 ng/mL on day 1 and 388 ng/mL on day 14. From day 2 to day 12, the mean pre-dose concentrations ranged from 7.33 ng/mL to 7.73 ng/mL. At 350 mg b.i.d., C_{max} corresponded to 453 ng/mL on day 1, and 506 ng/mL on day 14. From day 2 to day 12, the mean pre-dose concentrations ranged from 105 to 126 ng/mL. After repeated daily administrations of 350 mg for 14 days (i.e., on Day 14), BIO101 C_{max} and $AUC_{0-\tau}$ were higher (increase of about 30% for C_{max} and 16% for $AUC_{0-\tau}$), on average, in study participants administered 350 b.i.d. (506 ng/mL; 2768 ng*h/mL) than those administered q.d. (388 ng/mL; 2389 ng*h/mL). The b.i.d. administration was therefore selected as it allowed continuous pre-dose plasma concentrations close to the pharmacologically active dose of 140 ng/mL during the whole 14 days of the MAD period.

The confidential information in this document is provided to you as an investigator, potential investigator or consultant for review by you, your staff and applicable Independent Ethics Committee or Review Board. It is understood that the information will not be disclosed to others without written authorization from Biophytis S.A.

Administration of 450 mg b.i.d. generated a C_{max} of 524 ng/mL on day 1 and 560 ng/mL on day 14. From day 2 to day 12, the pre-dose concentrations ranged from 109 ng/mL to 151 ng/mL. $AUC_{0-\tau}$ was 2,429 ng*h/mL on day 1 and 3,203 ng*h/mL on day 14.

No accumulation of BIO101 was observed after q.d. administration of 350 mg BIO101 for 14 days (mean Rac= 1.14) whereas a slight accumulation was observed after b.i.d. administrations of BIO101 at 350 mg and 450 mg for 14 days (mean Rac= 1.31 in both panels).

Median T_{max} was the same (i.e., 3 h) in all dose groups and after the first and last doses. Mean BIO101 half-life was short with values approx. 2.8 and 4.4 h in all cohorts. At 350 mg and 450 mg b.i.d., after the day 14 morning administration, mean C_{max} and $AUC_{0-\tau}$ increased by about 1.11-fold and 1.16-fold for a 1.29-fold dose increase (from 350 mg to 450 mg), so less than dose proportionally. Based on the above results, no clinically meaningful accumulation is expected over the 26-week administration.

Based on the pharmacokinetic parameters, both 350 mg and 450 mg did show interesting profiles. On this basis, the b.i.d. administration was confirmed to be used for the next development phase of BIO101.

Pharmacodynamic effects of BIO101 were analyzed as individual changes compared to baseline. On average, BIO101 led to a reduction of biomarkers related to the renin-angiotensin system (e.g. aldosterone and renin levels) in study participants administered with 450 mg b.i.d. of BIO101, whereas no decrease was observed in study participants administered with placebo or administered 350 mg q.d. or b.i.d. A reduction of myoglobin and CK-MB (biomarkers related to muscle breakdown/damage) serum levels (vs baseline) was observed in particular and more markedly in study participants administered 350 or 450 mg b.i.d. However, a less pronounced reduction over time was also observed for the placebo group.

A trend to a slight increase in PIIINP (biomarker of anabolic response) plasma levels, vs baseline, was observed after 14 days of q.d. or b.i.d. administrations of 350 mg or 450 mg BIO101 compared to placebo and mainly at the highest dose of BIO101. Overall, BIO101 pharmacodynamic trends were mainly observed in b.i.d. dosing and especially at 450 mg, as compared to q.d. administration.

In conclusion, based on the pharmacokinetics and safety parameters of the MAD part, the administration of 350 mg b.i.d. was selected as the highest dose to be tested in the phase 2 study. This dose was well tolerated as it generated few numbers of AEs with mild severity. The dose of 350 mg b.i.d. generated a C_{max} of 346 ng/mL and circulating ranges (105-126 ng/mL) of BIO101 at pre-doses that corresponded to estimated pharmacological active doses (140 and 480 ng/mL) obtained in rodents and human myocytes assays. The dose of 175 mg b.i.d. was selected as a second dose for the phase 2 study. This dose being lower than 350 mg b.i.d., is anticipated to be safe and well tolerated. The 175 mg b.i.d. was not evaluated during the MAD part. However, based on simulations, this dose would lead to a C_{max} of 170-200 ng/mL on the first day and to a pre-dose ranges of 17-34 ng/mL for the following days. At C_{max}, the dose of 175 mg b.i.d. would lead to doses close to the IC50 myostatin gene expression dose.

2.2.3.2 Phase 2

BIO101 is currently under investigation for the treatment of age-related sarcopenia, in the study named: *Safety and efficacy of BIO-101 175 mg b.i.d. and 350 mg b.i.d. 26-week oral administration to patients suffering from age-related SARcopenia, including sarcopenic obesity, Aged ≥ 65 years and at risk of mobility disability. A double-blind, placebo- controlled, randomized INTerventional clinical trial (SARA-INT)*; clinicaltrials.goc identifier: NCT03452488.

The confidential information in this document is provided to you as an investigator, potential investigator or consultant for review by you, your staff and applicable Independent Ethics Committee or Review Board. It is understood that the information will not be disclosed to others without written authorization from Biophytis S.A.

To date, the SARA-INT study has recruited 231 participants into 3 treatment arms (BIO101 350mg B.I.D.; BIO101 175mg B.I.D.; placebo). The study duration is 6 months and the primary end point is the 400-meter walk test. 53 participants have completed the study medication to date.

During the ongoing study, the safety data is routinely evaluated by an independent Data Safety Monitoring Board (DMC). The DMC has met, so far, 5 times and concluded that the benefit/risk ratio of BIO101 in the study population remains favorable. The only limitation that was imposed was a temporary suspension of site visits during the COVID-19 outbreak and to replace on-site visits with safety phone calls. The DMC also recommended to continue supplying the SARA-INT study medication by direct delivery to the participants' homes.

2.3 Scientific Rationale

The novel infectious disease, COVID-19, caused by SARS-CoV-2, was first detected in Wuhan, China, in December 2019[5] and has spread rapidly, reaching pandemic proportions.

As for other coronaviruses such as SARS-CoV-1, responsible for the SARS outbreak in 2003, the angiotensin converting enzyme-2 (ACE2) expressed in pulmonary epithelial and endothelial cells is the receptor recognized by the spike protein of SARS-CoV-2[6]. ACE2, part of the renin angiotensin system (RAS), converts angiotensin II (Ang II) to angiotensin 1-7 (Ang-1-7). Ang-1-7 mediates anti-inflammatory, anti-oxidative and vasodilatory effects through binding with the Mas receptor (MasR)[7]. Conversely, ACE converts angiotensin I (Ang I) to angiotensin II (Ang II). Binding of Ang II to its receptor (Ang II receptor type 1 (AT1)) induces vasoconstrictive, pro-inflammatory and pro-oxidative effects[8]. The ACE/Ang II/AT1 and ACE2/Ang-1-7/MasR axes are known as the "harmful" and "protective" arms of the RAS, respectively.

Based on the knowledge accumulated with SARS-CoV-1, it is believed that the interaction of SARS-CoV-2 with ACE2 down regulates ACE2 activity, which results in a lower production of the vasodilator Ang-1–7 and in an excessive production of Ang II by ACE, leading to a general dysregulation of RAS. The imbalance between the "protective" and the "harmful" arms of RAS appears to play a central role in the acute lung injury (ALI)/acute respiratory distress syndrome (ARDS) associated with COVID-19[9]. Indeed, inhibition of the "protective" ACE2/Ang-1-7/MasR arm of RAS in favor of the "harmful" ACE/Ang II/AT1 axis signaling seems to be the cause of pulmonary vasoconstriction and inflammatory / oxidative organ damage, ultimately progressing towards ALI/ARDS in SARS-CoV-2 infected patients[10]. This theory is supported by a recent study by Liu and colleagues, showing that serum Ang II levels in COVID-19 patients were significantly higher than in non-infected individuals, and more importantly, were linearly associated with viral load and lung injury[11,12].

A number of studies have demonstrated the potential of ACE2/Ang-1-7/Mas axis stimulation in producing beneficial effects on lung tissue and respiratory function. This is notably the case in the context of pulmonary emphysema[13], lung fibrosis[14], pulmonary hypertension[15], lung inflammation[16] and cigarette smoking[17].

→Since direct inhibition of the harmful RAS axis by ACE inhibitors is known to induce adverse respiratory effects[18] and the use of AT1 receptor blockers (ARBs) such as Losartan is known to induce ACE2 expression[19] (the entrance door of the virus), we propose that a direct activation of the protective arm of the RAS axis, downstream of ACE2, through MasR activation is promising and may be a potent treatment option to restore RAS balance and subsequently protect patients infected by SARS-CoV-2 from ALI/ARDS.

The confidential information in this document is provided to you as an investigator, potential investigator or consultant for review by you, your staff and applicable Independent Ethics Committee or Review Board. It is understood that the information will not be disclosed to others without written authorization from Biophytis S.A.

Biophytis is developing BIO101, an investigational new drug intended for sarcopenia[20] and Duchenne muscular dystrophy (DMD)[21] for which the company has received IND from the Food and Drug Administration (FDA) in the US. BIO101 is an oral preparation of immediate-release 20E at ≥ 97% purity. It is extracted from the plants Cyanotis spp. 20E is a polyhydroxylated sterol found in plants and pharmacologically active in mammals[22]. BIO101 activates MasR on the protective arm of RAS. The engagement of MasR by BIO101 is responsible for a number of preclinical beneficial activities in normal and pathological contexts.

Of particular interest, 20E has shown anti-inflammatory effects in vivo in a mice model of acute lung injury (ALI)[23]. Indeed, 20E treatment reduced inflammatory cytokines (TNF-α, IL-2, IL-6, IL-8) levels and increased expression of anti-inflammatory (IL-4, IL-10) cytokines 42[24]. Modulation of inflammation by 20E was associated with a decrease of the lung damage as shown by histological examination of animal lungs. Similarly, two other studies in rat models of ALI, demonstrated that 20E treatment protected the animals by increasing the serum level of an anti-inflammatory cytokine (IL-10) and increased IL-10 mRNA expression in the lung tissue[25]. Li and collaborators demonstrated that 20E inhibited the TLR4 pathway, leading to a reduction of the expression of TNF-α and the promotion of surfactant protein A release by pneumocytes that finally improve respiratory functions[26].COVID19 positive patients often have symptoms of fever, dry cough, breathing difficulties (dyspnea), headache, and pneumonia that can worsen to ARDS. Disease onset may result in progressive respiratory failure owing to alveolar damage and death. The ARDS is characterized by the rapid onset of severe hypoxic respiratory failure and alterations in pulmonary mechanics. Indeed, the main physiological abnormalities of ARDS are hypoxemia, a reduced capacity to eliminate CO_2 and reduced lung volumes and compliance[27].

We have previously shown that oral BIO101 treatment ameliorated the decline in respiratory function in an animal model of Duchenne muscular dystrophy challenged with a bronchoconstrictor agent (methacholine). By investigating mice respiratory functions as well as lung mechanical properties (using non-invasive whole body plethysmography and FlexiVent analysis respectively), we demonstrated that this beneficial effect on respiratory function was not only associated with breathing parameters (inspiratory and expiratory time and frequency) as demonstrated by enhanced pause (Penh) measurements but also by an improvement of deep airway structure. Indeed, BIO101 treatment is characterized by an improvement of the resistance, compliance and elastance of animal lungs. These observations reflect a time-dependent protection from pulmonary function decline in the experimental model used[28].

The confidential information in this document is provided to you as an investigator, potential investigator or consultant for review by you, your staff and applicable Independent Ethics Committee or Review Board. It is understood that the information will not be disclosed to others without written authorization from Biophytis S.A.

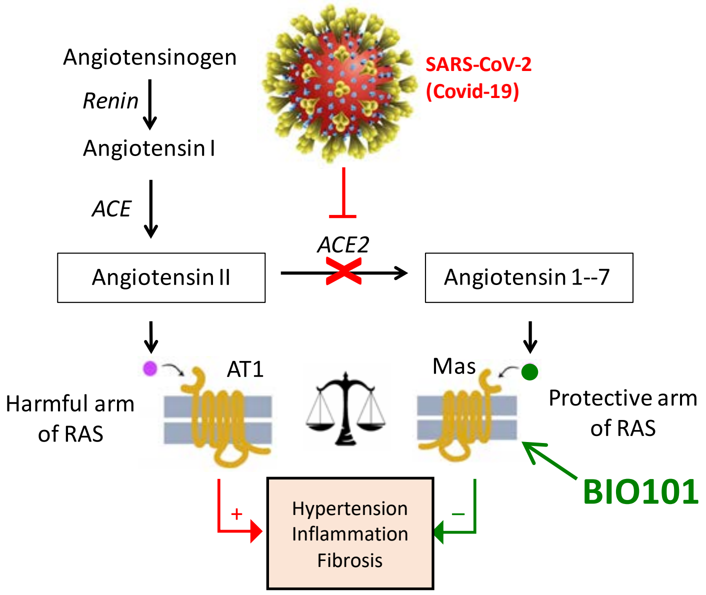

Figure 2: The Mechanism, by Which SARS-CoV-2 Causes Respiratory Deterioration in COVID-19 and the Potential Role of BIO101

Until now, the main supportive therapy in acute respiratory distress syndrome patients is mechanical ventilation, which represents a life-saving treatment for patients unable to sustain adequate ventilation. Unfortunately, mechanical ventilation may have side effects affecting both lungs and diaphragm. The mechanical ventilation can worsen the lung injury (ventilator-induced lung injury, VILI)[29] but can also impact diaphragm muscle by a phenomenon called Ventilator-induced diaphragmatic dysfunction (VIDD)[30]. This alteration is characterized by a muscle contractile dysfunction and a rapid muscular atrophy that are linked to RAS[31]. Indeed, angiotensin II can induce diaphragm muscle wasting and respiratory muscle dysfunction[32] whereas Angiotensin-(1–7) has been shown to exert a protective action in a rat model of VIDD[33].

Altogether, we strongly believed that BIO101 administration, by acting on the protective arm of RAS via its Mas receptor, could have a beneficial effect not only at the early stage of COVID19 infection but, based on its mechanism of action, could also have beneficial effects by preserving diaphragm strength and structure of intensive care unit patients and lead to improved ARDS outcome.

2.4 Benefit / Risk Assessment

2.4.1 The Unmet Need

COVID-19 and its complication pose a significant burden on patients, which currently does not have an approved treatment. Patients with a severe form of COVID-19 are at a high risk for development of respiratory failure, require mechanical ventilation and mortality. This is especially noted in the older age groups (60 and above). The evolution of COVID-19, from onset to critical state is fast. The median time from onset to ICU placement, in a case-serial from China, was 12 days[34].

The following are key issues that impact the health state of individuals with severe COVID-19:

- The risk of developing ARDS, in part, due to the interaction of the virus with ACE2
- The risk of developing severe sepsis and septic shock
- The risk of deterioration, due to muscle wasting, which is associated with ARDS

The confidential information in this document is provided to you as an investigator, potential investigator or consultant for review by you, your staff and applicable Independent Ethics Committee or Review Board. It is understood that the information will not be disclosed to others without written authorization from Biophytis S.A.

2.4.2 The Potential Role of BIO101 in the Treatment of COVID-19

While there is no specific COVID-19 pre-clinical data with BIO101, there is a reason to believe that it will offer a significant benefit for patients with severe COVID-19 symptoms. This is based on the following:

- BIO101 has been shown to improve muscle function in aging animals and in an animal model of DMD
- BIO101 has been shown to improve respiratory function in an animal model of DMD
- BIO101 has been shown to attenuate the damage, seen in a model of an acute lung injury

2.4.3 The Risk Profile of BIO101

To date, the only known adverse event to be watched-out for, with BIO101 is the potential for abuse, misuse and diversion by athletes and body builders. Participants in studies with BIO101 are advised not to allow access to this medication to anyone but themselves and only to take the medication at the indicated time points specified within the protocols.

Due to LFT elevation that were seen in the 26 weeks toxicity studies, it was recommended not to include participants with an active biliary disease or with LFT abnormalities in studies of BIO101. Because no liver abnormalities were seen in the 39 weeks toxicity study and that no signal of liver injury that could be attributed to BIO101 in the SARA-INT study, based on 53 completers, and due to the short period of treatment (28 days, compared to 6 months in the SARA-INT study), we will allow inclusion of participants with non-active biliary disease and will increase the upper limit of LFT elevation from x2 (as was practiced in the SARA-INT study) to x5. Stopping rules related to LFT will be employed as well and are discussed in the safety section of this protocol. Performance of abdominal imaging, at recruitment to the study is not possible as it involves an increased risk of exposure of radiology staff, so no liver imaging will be done.

In the phase 1 studies, the most common AE that was reported and in a higher proportion in the treated participants, was gastrointestinal symptoms (e.g. abdominal pain and diarrhea). However, most of these events were mild and did not require stopping the study medication.

Despite the fact that BIO101 does not reduce blood pressure in hypertensive animals and in the phase I studies, a special attention has been given to events of documented orthostatic hypotension in the phase 2 SARA-INT trial. These events are recorded as adverse events of special interest (see more information in section 7.2).

It should be noted, that in the 26-weeks dog study, mild degeneration of the testicular germinal cells was noted. The implication of this finding is not clear and is still under investigation. So far it was not seen in any of the other non-clinical toxicity studies. In this study, we will monitor male fertility by measurements of testosterone, luteinizing hormone (LH), follicle-stimulating hormone (FSH) and inhibin B, with a calculation of the FSH / Inhibin B ratio (Stewart et al., 2005; Esposito et al., 2018; Damyashkin, 2019)[35,36,37]. An additional discussion on this issue will be included in Section 4.1.1 on the target population.

2.4.4 Risks Associated with the COVID 19 Epidemic

The reproduction rate of SARS-CoV-2 is relatively high (around 3.26[38]). The World Health Organization (WHO) has issued a set of guidelines for Infection Prevention and Control (IPC). Individuals who have a mild presentation of COVID-19 are instructed to practice social isolation and to stay at home, while individuals with severe and critical presentation are hospitalized in designated departments where IPC is strictly controlled.

The confidential information in this document is provided to you as an investigator, potential investigator or consultant for review by you, your staff and applicable Independent Ethics Committee or Review Board. It is understood that the information will not be disclosed to others without written authorization from Biophytis S.A.

In this study, the target population is within the range of severe presentation and, therefore, all study participants will be hospitalized in a designated unit for the entirety of the study unless they are deemed as improved and can be discharged to a lower level facility or home. Such an occurrence is considered as a 'positive' event (see more information in section 3.3) and will mark the end-of-study intervention. Any further follow-up with participants who are not in a designated facility, will take place over the phone and there will be no further contact between the site staff and the participant unless otherwise indicated (e.g. in case of an SAE, which will require re-admittance to a designated facility).

2.4.5 Conclusion

BIO101 has been tested in a number of pre-clinical toxicology studies (up to 39 weeks of exposure). The phase 1 study showed a profile of a safe and well-tolerated medication. Repeated assessments by the DMC during the phase 2 SARA-INT study has concluded that the benefit / risk profile of BIO101 is favorable. The main concern remains the potential toxicity of BIO101 on the hepatobiliary system. This risk will be mitigated by implementation of stopping rules based on LFT. In addition, reports of orthostatic hypotension will be specifically assessed in case they warrant a further safety signal.

It is important to mention, that we are targeting a high-risk population with a risk for serious deterioration and mortality. Therefore, the overall benefit / risk ratio of BIO101 in this population, is favorable.

3 Objectives and Endpoints

The study has 2 parts and each part has its own goals and objectives.

3.1 Part 1

- Ascertain the safety and tolerability of BIO101, in order to begin recruitment to part 2
- Obtain preliminary indication of activity of BIO101, in preventing respiratory deterioration in the target population

3.2 Part 2

- Re-assess the sample size that is needed for the confirmatory part of the study
- Provide confirmation on the benefit of BIO101 in the target population
- Identify and assess potential biomarkers for further understanding of the effect of BIO101 in the target population

3.3 Endpoints

3.3.1 Proportion and Time to Events

The key events for this study are respiratory deterioration and all-cause mortality, as 'negative' events, and official discharge from hospital care by the department due to improvement in participant condition, as a 'positive' event. For the purpose of this study, respiratory deterioration will be defined as any of the following:

- Requiring mechanical ventilation (including cases that will not be intubated due to resource restrictions and triage)
- Requiring extracorporeal membrane oxygenation (ECMO)
- Requiring high-flow oxygen defined as delivery of oxygen at a flow of ≥16 L/min.

The confidential information in this document is provided to you as an investigator, potential investigator or consultant for review by you, your staff and applicable Independent Ethics Committee or Review Board. It is understood that the information will not be disclosed to others without written authorization from Biophytis S.A.

These will be recorded for each treatment arm and from those the proportion of events, per number of participants, per arm and the cumulative time to these events, will be extrapolated.

Participants will only stay on treatment until a clinical endpoint is reached (i.e., 'negative' or 'positive' event). Participants will continue to be followed in the study after treatment is discontinued through phone calls 14 (± 2) and 60 (± 4) days after the last on-intervention visit (see Section 4.3.4.1).

3.3.2 Assessment of Participant General and Respiratory Status

3.3.2.1 The National Early Warning Score 2 (NewS2)

NEWS2 is the latest version of the National Early Warning Score (NEWS), first produced in 2012 and updated in December 2017, which advocates a system to standardize the assessment and response to acute illness[39].

The NEWS is based on a simple aggregate scoring system in which a score is allocated to physiological measurements, already recorded in routine practice, when participants present to, or are being monitored in hospital.

3.3.2.2 The Acute Physiology and Chronic Health Evaluation (APACHE) II

APACHE II, is a severity of disease classification system. APACHE II uses a point score based upon initial values of 12 routine physiologic measurements, age, and previous health status to provide a general measure of severity of disease. An increasing score (range 0 to 71) was closely correlated with the subsequent risk of hospital death for 5815 intensive care admissions from 13 hospitals. This relationship was also found for many common diseases. When APACHE II scores are combined with an accurate description of disease, they can prognostically stratify acutely ill patients and assist investigators in comparing the success of new or differing forms of therapy. This scoring index can be used to evaluate the use of hospital resources and compare the efficacy of intensive care in different hospitals or over time[40].

In a study that investigated the capability of APACHE II to predict survival and neurological outcomes at 30 days after out-of-hospital cardiac arrest, it was shown that a difference of 4-5 points at admission was seen between the group of survivors and non-survivors and a similar difference was seen between the group of good and poor neurological outcome[41].

3.3.3 Biomarkers

The following biomarkers will be measured during the study:

- Arterial blood gas analysis (ABG)
- Viral load (in nasal/nasopharyngeal swab samples)
- RAS / MAS markers:
    - Angiotensin 1-7 (Ang 1-7)
    - Angiotensin 1-5 (Ang 1-5)
    - Angiotensin 2
    - Renin
    - Aldosterone
    - Angiotensin-converting enzyme (ACE) levels
- Inflammatory markers including:

The confidential information in this document is provided to you as an investigator, potential investigator or consultant for review by you, your staff and applicable Independent Ethics Committee or Review Board. It is understood that the information will not be disclosed to others without written authorization from Biophytis S.A.

  - IL-2, 6 and 10
  - TNFα
  - Troponin (I and T)
  - LDH
  - D-dimer
  - CRP plasma levels
  - Ferritin
- Skeletal muscle proteins including CK-MM, CK-MB, myoglobin, CK macro type I, CK macro type II
- PIIINP
- bone specific alkaline phosphatase

The confidential information in this document is provided to you as an investigator, potential investigator or consultant for review by you, your staff and applicable Independent Ethics Committee or Review Board. It is understood that the information will not be disclosed to others without written authorization from Biophytis S.A.

3.3.4 Pharmacokinetics of BIO101 and its Metabolites

The following parameters, where appropriate, will be determined for BIO101 and its metabolites, poststerone, 14-alpha-deoxy-poststerone (14d Post) and 14-deoxy-20-hydroxyecdysone (14d20E), from individual concentration-time profiles in plasma and urine, using non-compartmental analysis and actual sampling time:

Table 2: Pharmacokinetic (PK) measurements

**C_{max}**	maximum observed plasma concentration
**t_{max}**	time of occurrence of C_{max}
**C_T**	trough plasma concentration observed at the end of the dosing interval (i.e., 12 or 24 hours postdose)
**AUC_{0-t}**	area under the plasma concentration-time curve from time zero till the time corresponding with the last observed quantifiable concentration calculated by the linear up - logarithmic down trapezoidal rule
**AUC_T**	area under the plasma concentration-time curve over the dosing interval (i.e., 12 or 24 hours) calculated by the linear up - logarithmic down trapezoidal rule
**$AUC_{0-\infty}$**	area under the plasma concentration versus time curve from time zero to infinity, calculated from AUC_{0-t} + (Ct/λ_z), where Ct is the last observed quantifiable concentration and λ_z the first order terminal rate constant
**C_{avg}**	average plasma concentration calculated as AUC_T/T, with T being the dosing interval
**$t_{1/2,\ \lambda z}$**	the apparent terminal half-life, calculated from (ln 2)/λ_z
**R_{ac}**	accumulation ratio, calculated as AUC_T Day 14/$AUC_{0\ 24h}$ (or AUC_{0-12h}) Day 1
**Ae**	cumulative amount excreted in urine expressed in mass unit and %dose
**CLR**	the renal clearance, calculated as Ae/AUC, where Ae and AUC are calculated over the same interval

Dose normalized parameters (C_{max}/dose, C_{24h} or C_T/dose, AUC_{0-24h} or AUC_T/dose, Ae%) will also be assessed.

3.3.5 Assignment of Study Endpoints

This section provides an overview of all study endpoints which will be followed by overviews of endpoints to be included in each of the planned interim analyses and the final analysis.

The confidential information in this document is provided to you as an investigator, potential investigator or consultant for review by you, your staff and applicable Independent Ethics Committee or Review Board. It is understood that the information will not be disclosed to others without written authorization from Biophytis S.A.

3.3.5.1 Overview of all Study Endpoints

Primary Efficacy Endpoint

- Proportion of participants with 'negative' events, of either of the following:
  - All-cause mortality
  - Respiratory failure, defined as any of the following:
    - Requiring mechanical ventilation (including cases that will not be intubated due to resource restrictions and triage)
    - Requiring ECMO
    - Requiring high-flow oxygen

Key Secondary Efficacy Endpoints

- Proportion of participants with events of respiratory failure, defined as any of the following:
  - Requiring mechanical ventilation (including cases that will not be intubated due to resource restrictions and triage)
  - Requiring ECMO
  - Requiring high-flow oxygen
- Proportion of participants with 'positive' events:
  - official discharge from hospital care by the department due to improvement in participant condition (self-discharge by participant is not considered a positive event)
- Proportion of participants with events of all-cause mortality

Additional Secondary Endpoints:

- SpO_2/FiO_2
- Oxygen saturation in arterial blood, measured by pulse-oximetry (SpO_2)
- Time to events, of either of the following:
  - All-cause mortality
  - Respiratory failure, defined as any of the following:
    - Requiring mechanical ventilation (including cases that will not be intubated due to resource restrictions and triage)
    - Requiring ECMO
    - Requiring high-flow oxygen
- Time to event: official discharge from hospital care due to improvement
- The National Early Warning Score 2 (NewS2)
- Proportion of participants with CPAP/BiPAP events, defined as requiring CPAP/BiPAP in participants entering the study on low flow oxygen)

- For participants who experienced a 'positive' event: proportion of participants with sustained positive outcome
- Population-PK study (pop-PK)

Exploratory Measures:

- Viral load in nasal/nasopharyngeal swabs
- Arterial blood gas analysis (ABG)
- PaO_2/FiO_2

The confidential information in this document is provided to you as an investigator, potential investigator or consultant for review by you, your staff and applicable Independent Ethics Committee or Review Board. It is understood that the information will not be disclosed to others without written authorization from Biophytis S.A.

- RAS / MAS biomarkers:
  - Angiotensin 1-7 (Ang 1-7)
  - Angiotensin 1-5 (Ang 1-5)
  - Angiotensin 2
  - Renin
  - Aldosterone
  - Angiotensin-converting enzyme (ACE) levels
- Inflammatory markers including:
  - IL-2, 6 and 10
  - TNFα
  - Troponin (I and T)
  - LDH
  - D-dimer
  - CRP plasma levels
  - Ferritin
- Skeletal muscle proteins including CK-MM, CK-MB; Myoglobin, CK macro type I, CK macro type II
- PIIINP
- bone specific alkaline phosphatase
- Acute Physiology and Chronic Health Evaluation (APACHE) II score

Safety and tolerability to BIO101:

- SUSARs, SAEs, AESIs, AEs
- Vital signs
- Safety labs
- ECGs

3.3.5.2 Part 1

3.3.5.2.1 Endpoints for the Interim Safety Analysis, at the End of Part 1

This interim analysis is intended to facilitate the decision of go/no-go for Part 2 of the study. This decision will be made by the DMC based on safety and tolerability data:

- SUSARs, SAEs, AESIs, AEs
- Vital signs
- Safety labs (including testicular biomarkers)
- ECGs

3.3.5.2.2 Endpoints for the Interim Analysis for Indication of Activity, at the End of Part 1

This interim analysis is intended to provide an indication of activity.

The confidential information in this document is provided to you as an investigator, potential investigator or consultant for review by you, your staff and applicable Independent Ethics Committee or Review Board. It is understood that the information will not be disclosed to others without written authorization from Biophytis S.A.

Time frame – up to 28 days:

Primary:

- Proportion of participants with ‘negative’ events, of either of the following:
  - All-cause mortality
  - Respiratory failure, defined as any of the following:
    - Requiring mechanical ventilation (including cases that will not be intubated due to resource restrictions and triage)
    - Requiring ECMO
    - Requiring high-flow oxygen

Secondary:

- SpO_2/FiO_2
- Inflammatory markers including:
  - IL-6
  - TNFα
  - D-dimer
- RAS / MAS biomarkers:
  - Angiotensin 2
  - Angiotensin-converting enzyme (ACE) levels

3.3.5.3 Part 2

3.3.5.3.1 Endpoints for the Interim Analysis

This interim analysis is intended to support the reassessment of the sample size for Part 2.

Time frame – up to 28 days:

Primary:

- Proportion of participants with ‘negative’ events, of either of the following:
  - All-cause mortality
  - Respiratory failure, defined as any of the following:
    - Requiring mechanical ventilation (including cases that will not be intubated due to resource restrictions and triage)
    - Requiring ECMO
    - Requiring high-flow oxygen

3.3.5.3.2 Endpoints for the Final Analysis at the End of Part 2

All study endpoints described under Section 3.3.5.1 will be included in the final analysis with a time frame of up to 28 days.

The confidential information in this document is provided to you as an investigator, potential investigator or consultant for review by you, your staff and applicable Independent Ethics Committee or Review Board. It is understood that the information will not be disclosed to others without written authorization from Biophytis S.A.

4 Study Design

This is a double-blind, placebo-controlled, group sequential and adaptive study in 2 parts:

Table 3: The Operating Principals of the COVA Study

<table>
<tr><th>Part</th><th>Goal</th><th>Number of participants</th></tr>
<tr><td>1</td><td>Obtain indication of activity of BIO101, about the effect of BIO101 in preventing further respiratory deterioration</td><td>50
1:1 randomization
Planned information fraction: 16%</td></tr>
<tr><td rowspan="2">2</td><td>Re-assess the sample size for step 2</td><td>155 (an addition of 105 participants)
1:1 randomization
Planned information fraction: 50%</td></tr>
<tr><td>Confirmation of the effect of BIO101 in preventing further respiratory deterioration and obtaining a conditional approval</td><td>310, potentially increased by 50% (up to 465, based on interim analysis 2)
1:1 randomization
Planned information fraction: 100%</td></tr>
</table>

All the study participants, regardless of timing of recruitment, will be recruited for an intervention period of 28 days, with BIO101 at 350mg B.I.D. or matching placebo, taken orally as a capsule form. They will all undergo the same assessments. For communication purposes with investigators, authorities and relevant stakeholders, the participant will be identified according to the part during which they were recruited.

The data from all study participants will be pooled for analysis of the primary and secondary endpoints at the end of part 2. In addition, the data from part 1 participants will be analyzed in 2 sets of unblinded interim analyses, conducted by an independent Data Monitoring Committee (DMC). The layout of the interim and final analysis is as follows:

The confidential information in this document is provided to you as an investigator, potential investigator or consultant for review by you, your staff and applicable Independent Ethics Committee or Review Board. It is understood that the information will not be disclosed to others without written authorization from Biophytis S.A.

Table 4: COVA Study Decisions and Implementation

Part	Goal	Steps
1	Provide evidence, to support the need for a confirmatory investigation on the benefit of BIO101 in the target population	1. Interim 1, Safety analysis: based on safety and tolerability, to facilitate the decision of go / no-go to part 2.<br>2. Interim 1, 'indication of activity' analysis: based on the numeric improvement in the primary endpoint, and select secondary and exploratory endpoints: SpO_2/FiO_2, the inflammatory markers: IL6 and TNF-α and D-Dimers, and the ANG-2 and ACE2 levels), to provide an indication of activity.
2	Reassessment of the sample size for part 2	By utilizing the method of 'promising zone' analysis.
	Provide confirmatory evidence, for the benefit of BIO101in the target population	

Table 5: COVA Study Interim Analysis Information Summary

	Interim Analysis Part 1	Interim Analysis Part 2
Timing	End of Part 1 (up to 28 days)	When approximately 155 participants are evaluable for the primary endpoint (up to 28 days)
Purpose	Safety and efficacy (see Table 4)	Efficacy (see Table 4)
Population	Safety population (safety)<br>ITT population (efficacy)	ITT population

4.1 Rationale for Study Design

This study is designed to address an acute and serious situation, with a significant impact on public and individual health. The scientific rational for the study is solid, but there is a lack of pre-clinical data to help in designing this study as is usually done in classical clinical development. The urgency dictates a short and efficient planning and this is why the study is designed as group sequential and adaptive:

- Group sequential – to allow an efficient run-through, from obtaining an early indication of activity to a final confirmation
- Adaptive – to allow accumulation of early data and adapt sample size in part 2 in order to inform the final design of the confirmatory part of the trial

This design allows for achieving the following advantages:

The confidential information in this document is provided to you as an investigator, potential investigator or consultant for review by you, your staff and applicable Independent Ethics Committee or Review Board. It is understood that the information will not be disclosed to others without written authorization from Biophytis S.A.

- Operational excellence: by reducing the need for repeated study and site initiations through the development process
- Optimal utilization of data: allowing the use of data from part 1 participants for the final, part 2 analysis reduces the number of exposed participants to a clinical study environment and potential placebo treatment
- Stratification: while part 1 aims to recruit a limited sample size, it still allows stratification according to RAS pathway modulator use (Yes/No).

4.1.1 Justification of Selection of Study Population

4.1.1.1 Age and Severity

As discussed earlier in section 2.4 on the benefit / risk assessment of BIO101, COVID-19 is a disease, in which patients, especially when manifesting severe respiratory symptoms, can benefit from the treatment with BIO101.

When the study was originally designed, the decision was made to focus on patients with severe respiratory manifestations, aged 18 and above. However, the pandemic has changed in manner quite rapidly. A few treatment options have emerged and there is a better understanding of the risk factors. As for age, it seems that patients aged 45 and above, make up more than 50% of all patients, they also make around 60% of patients with severe manifestations, and more than 75% of patients with critical manifestations (CDC COVID-19 response team, March 2020; Verity et al., 2020; Zhang et al., 2020)[42,43,4445].

Indeed, age above 45 is an important risk factor for hospitalization, ICU placement, mechanical ventilation and mortality (Grasseli et al., 2020; Livingston and Bucher, 2020; Nikolich-Zugich et al., 2020; Zheng et al, 2020; Zhou et al., 2020)[46,47,48,49,50]. It is hypothesized, that these differences could be caused by age-related deterioration of the immune system (Meftahi et al., 2020)[51] or alteration in RAS and especially in relation to ACE2 (Bourgonje et al., 2020; Cheng et al., 2020; Mueller et al., 2020; Nicolich-Zugich et al., 2020)[52,53,54,55]. The latter possibility makes this age group very relevant to this study, due to the mechanism of action of BIO101, which is by activation of the protective arm of the RAS.

With all the above put together, it seems most appropriate, to target patients with severe respiratory manifestations of COVID-19 and who are 45 years old and above. This is also in line with a potential male fertility risk, albeit remote, that was seen, in the form of a mild testicular germinal cells in 2 dogs out of 4, in the 26-weeks toxicity study. While this finding was not repeated in the 39-week study in dogs, nor in the 26-weeks and the juvenile-tox studies in rats, it is still under investigation. Altogether, it makes this target population, aged 45 and above a more appropriate target, with a more positive benefit / risk ratio.

4.1.1.2 Use of CPAP/BiPAP

CPAP and BiPAP are not considered invasive respiration methods and individuals on these methods can continue to take oral medication. In France, CPAP is used, in some of the departments, as a measure, to prevent deterioration and avoid the need for invasive intubation. This is based on a recent two-period, case-control observational study showed that continuous positive airway pressure for therapeutic escalation in patients with SARS-CoV-2-related respiratory distress significantly reduced the number of patients intubated at a 7- and 14-days delay (Oranger et al., 2020)[56].

4.1.2 Justification for selection of endpoints

For this clinical study the following options were considered as end points:

The confidential information in this document is provided to you as an investigator, potential investigator or consultant for review by you, your staff and applicable Independent Ethics Committee or Review Board. It is understood that the information will not be disclosed to others without written authorization from Biophytis S.A.

- Clinical status endpoints:

  These include ordinal scales (such as the one used by Wang et al., 2020)[57] or endpoints that are based on clinical measurements (such as the SOFA, SAPS and APACHE). The disadvantage of these is that they require measurement in all participants at the same time point. Since BIO101 can only be given orally, requiring an intubation means the end of the treatment and that endpoint measurement will occur at any time point, during the 28 days of treatment.

  In addition, scales that are best on clinical measurement, require obtaining arterial blood gasses measurements, which are not a standard in a medical ward, where most of the study participants are expected to be recruited.

- Endpoints that are based on lab results

  These include viral load and markers of inflammation. While these are going to be used, as part of the investigation, during part 1, to obtain evidence of activity of BIO101, and as supporting endpoints for the final analysis, we do not feel that they properly reflect the benefit that is needed for the target participants. Therefore, they are not selected as primary or key secondary endpoints for this study.

- Endpoints that reflect outcomes

  We feel that these are most appropriate to reflect the benefit that the target participants need. Of these, the sum of negative events all-cause mortality and respiratory deterioration are deemed most important and serve as a primary endpoint for this study. Proportion is preferred to number of events, in case the treatment arms are not of the exact size.

4.1.3 Justification of randomization scheme

While sometimes, when testing a potentially beneficial medication, for severe conditions, a higher chance to be on treatment is desired, leading to the selection of a 2:1 randomization scheme, a randomization scheme of 1:1 is most optimal, from the perspective of sample size and is associated with the lowest placebo effect.

4.2 Justification for Dose

Based on the pharmacokinetics and safety parameters of the SARA-PK phase 1 MAD part, the administration of 350 mg b.i.d. was selected as the highest dose to be tested in the ongoing phase 2 (SARA-INT) study. This dose was well tolerated as it generated few numbers of AEs with mild severity. The dose of 350 mg b.i.d. generated a C_{max} of 346 ng/mL and circulating ranges (105-126 ng/mL) of BIO101 at a pre-dose that corresponds to estimated pharmacological active doses (140 and 480 ng/mL) obtained in rodents and human myocytes assays. For this study, the treatment dose of 350mg b.i.d. is selected, since in the acute setting, there is a shorter window for treatment effect.

4.3 Study Assessments

All study assessments will be conducted, in an in-hospital setting, either in a standard medical, acute care, intermediate or intensive care units. Study assessments take place in 3 defined periods: pre-intervention, on-intervention and post-intervention.

The confidential information in this document is provided to you as an investigator, potential investigator or consultant for review by you, your staff and applicable Independent Ethics Committee or Review Board. It is understood that the information will not be disclosed to others without written authorization from Biophytis S.A.

4.3.1 Timing of Assessments

Ideally, study assessments should occur during the morning hours (especially when to comes to blood draws). However, it is understood, that with a dynamic condition such as severe COVID-19, and especially in an in-hospital service, this may not always be feasible. However, the following should be followed:

- **Study assessment should not be performed before an informed consent was signed.**
- PK assessment should follow the sampling schedule with taking into account time of study medication administration (the dose that is taken during the blood draws for the pop-PK study and the next dose) – see more information on PK blood draw timing in section4.3.3.3. A deviation from the planned timing of the sample by ± 10% is allowed.
- The day 28 and endpoint visit assessments, should be done as close as possible to the last medication dose (within 24 hours from last dose, if possible).
- If the first dose was given in the pm hours, for the next assessments should still be done, if possible, in the morning hours.
- Blood draws for biochemistry, should be done when the participant has fasted for at least 8 hours (water and tea are allowed).

4.3.2 Pre-intervention Period

These assessments are done in order to decide if the participant is eligible for participation and to provide the information on the pre-treatment, or 'baseline' status, to which the end of intervention assessment will be compared.

This period begins with the participant signing the informed consent and end with the first dose of study medication. No study related activities can take place before the participant has signed an ICF.

For screening assessments, where the assessments requested for the study are performed during routine care at the site, the results of such routine assessments can be used for the study and those assessments do not need to be repeated. However, the full panel of study assessments should be performed on the same study day (i.e., routine assessments and any additional non-routine study assessments on the same day).

4.3.2.1 Screening

These assessments include the ascertainment of carrier state of the potential participant, the pulmonary involvement and its defined level of severity and a number of evaluations to make sure that the potential participant meets all the inclusion criteria and none of the exclusion criteria for this study.

4.3.2.2 Baseline

Once the screening activities are finished and it is determined that the participant is eligible for the study, the baseline assessments can begin. They include the pre-first-dose assessments of clinical status and blood draws for biomarkers and the randomization. The final baseline activity is the administration of the first dose of study medication. This is considered as day 0 of the study.

The baseline assessments are the one, to which the final assessments are compared, when it comes to define a change from baseline.

The confidential information in this document is provided to you as an investigator, potential investigator or consultant for review by you, your staff and applicable Independent Ethics Committee or Review Board. It is understood that the information will not be disclosed to others without written authorization from Biophytis S.A.

4.3.3 On-intervention Period

Starting from the first dose of study medication and ending with the participant either reaching the study defined events (see more information about the events, in sections 4.4 and 7.1) or reaching the 28th day of treatment.

4.3.3.1 First Week Assessments

The first week after baseline is expected to be the most dynamic period during the study. Participants will be assessed daily based on the predefined study endpoints.

The data from part 1 participants on visit A3, will be used for the first interim analysis, at which point the evidence for indication of activity of BIO101 will be determined.

4.3.3.2 Day 14 and 28 Endpoint Visit Assessments

The assessments on these days will be similar to the ones that the participants undergo during the first week.

During part 1, these assessments will only take place, if the participants have not reached a study defined event (see section 7.1.1).

4.3.3.3 Population PK Study

Blood samples (6cc each) will be taken at Baseline and at the A2 visit at the following time points:

Table 6: Pop-PK study - schedule of blood draws

**Time point**	**Relation to administration of study medication**
**T0**	Right before morning dose
**T4**	4 hours (±10%) after morning dose
**T24**	24 hours (±10%) before morning dose of the subsequent day

4.3.3.4 Endpoint and Early Withdrawal

If the participant reached the predefined study events (see sections 3.3.1 and 7.1.1), study intervention will finish and the participant will undergo an endpoint visit assessment.

Participants who wish to withdraw from the study, before reaching the study pre-defined events and before reaching day 28 of treatment, can do so. Also, participants may be withdrawn because of reaching stopping rules (see section 7.2.8). In these cases, an attempt should be made to perform this visit, on the day a decision is made to stop participation.

At this stage, there are no plans to continue to supply the study medication, to participants who recovered and have been discharged to home.

The confidential information in this document is provided to you as an investigator, potential investigator or consultant for review by you, your staff and applicable Independent Ethics Committee or Review Board. It is understood that the information will not be disclosed to others without written authorization from Biophytis S.A.

4.3.4 Post-intervention Period

This period starts after the participant finished the endpoint or day 28 visit assessment. Study medication is stopped and the purpose of this period is to finalize the post-intervention follow-up. If there is no active AE, this period ends 60 days after the endpoint. If an AE is still active, this period may continue until the event is resolved or considered as not expected to change anymore. For more information on AEs, see the section 7.2.

4.3.4.1 Post-intervention Phone Calls

All participants, whether completing 28 days of participation or ending the study prior to that (see more details about definition of end-of-study participation, below), will undergo two phone-call follow ups for assessment of safety and AEs as well as information regarding participant's status (see below), follow-up 1, 14 (± 2) days after the last day of receiving the study medication and follow-up 2, 60 (± 4) days after the last day of receiving the study medication.

During these follow-up calls, information will be collected regarding safety, AEs, and concomitant medication and also regarding the participant's location since the last on-intervention visit (home, intensive care, regular hospital ward, including duration), regarding requirement and duration for oxygen supplementation since the last on-intervention visit and regarding the occurrence of positive or negative events since the last on-intervention visit.

4.4 Definition of End-of-study Intervention

A participant is considered as reaching an end-of-study intervention in one of the following situations:

- Reaching a state of respiratory failure, defined as any of the following:
    - Requiring mechanical ventilation (including cases that will not be intubated due to resource restrictions and triage)
    - Requiring ECMO
    - Requiring high-flow oxygen
- All-cause mortality
- Official discharge from hospital care by the department due to improvement in participant condition
- Finished 28 days on study medication

Participants who finish study participation before day 28 will undergo end-of-study assessments, similar to those who are taking place on the day 28 assessments, if possible. These participants will also have the 14 (± 2) and 60 (± 4) days, post-intervention phone calls.

For study withdrawal, see section 5.3.

5 Study Population

Currently, the recommendation is that individuals with mild form of COVID-19 will be treated at home, with social isolation as much as possible. Patients with severe manifestations are to be treated in a hospital. This means that it will be very difficult to conduct a clinical trial in mild patients (albeit not impossible). There is an important need to provide a treatment for patients who are at risk for significant deterioration or mortality. For this reason, this study focuses on severe patients.

The confidential information in this document is provided to you as an investigator, potential investigator or consultant for review by you, your staff and applicable Independent Ethics Committee or Review Board. It is understood that the information will not be disclosed to others without written authorization from Biophytis S.A.

Since the administration of BIO101 is oral, it would be very difficult to treat patients who are already intubated, which is why they are excluded from the study. We also want to make sure that we are targeting patients who are still in the dynamic phase of the evolution of COVID-19, which is why we will include patients who developed symptoms in the past week and came into a severe state not more than 4 days before start of study medication.

While there are no approved treatments for COVID-19, there is a wide use of antiviral, antimalarial and antimicrobial agents and medications that target the RAS system. We would not want to prevent study participants from access to medications that could be, potentially, offer some benefit.

The study participants are expected to remain in the treatment facility throughout the study. Reaching the study endpoints, either positive, or negative, will also mean the end of the study medication. See more information on study events in sections 3.3.1 and 7.1.1)

5.1 Inclusion Criteria

1. Age: 45 and older.
2. A confirmed diagnosis of COVID-19 infection, within the last 14 days, prior to randomization, as determined by PCR or other approved commercial or public health assay, in a specimen as specified by the test used.
3. Hospitalized, in observation or planned to be hospitalized due to COVID-19 infection symptoms with anticipated hospitalization duration >=3 days
4. With evidence of pneumonia based on **all** of the following:
    a. Clinical findings on a physical examination
    b. Respiratory symptoms developed within the past 7 days
5. With respiratory decompensation that started not more than 4 days before start of study medication and present at screening, meeting **one** of the following criteria, as assessed by healthcare staff:
    a. Tachypnea: ≥25 breaths per minute
    b. Arterial oxygen saturation ≤92%
    c. A special note should be made if there is suspicion of COVID-19 related myocarditis or pericarditis, as the presence of these is a stratification criterion
6. Without a significant deterioration in liver function tests:
    a. ALT and AST ≤ 5x upper limit of normal (ULN)
    b. Gamma-glutamyl transferase (GGT) ≤ 5x ULN
    c. Total bilirubin ≤ 5×ULN
7. Willing to participate and able to sign an informed consent form (ICF). Or, when relevant, a legally authorized representative (LAR) might sign the ICF on behalf of the study participant
8. Female participants should be:

    at least 5 years post-menopausal (i.e., persistent amenorrhea 5 years in the absence of an alternative medical cause) or surgically sterile;

    OR

    a. Have a negative urine pregnancy test at screening
    b. Be willing to use a contraceptive method as outlined in inclusion criterion 9 from screening to 30 days after last dose.

The confidential information in this document is provided to you as an investigator, potential investigator or consultant for review by you, your staff and applicable Independent Ethics Committee or Review Board. It is understood that the information will not be disclosed to others without written authorization from Biophytis S.A.

9. Male participants who are sexually active with a female partner must agree to the use of an effective method of birth control throughout the study and until 3 months after the last administration of investigational product;
   Note: medically acceptable methods of contraception that may be used by the participant and/or partner include combined oral contraceptive, contraceptive vaginal ring, contraceptive injection, intrauterine device, etonogestrel implant, each supplemented with a condom, as well as sterilization and vasectomy.
10. Female participant who are lactating must agree not to breastfeed during the study and up to 14 days after the intervention.
11. Male participants must agree not to donate sperm for the purpose of reproduction throughout the study and until 3 months after the last administration of investigational product;
12. For France only: Being affiliated with a European Social Security.

5.2 Exclusion Criteria

1. Not needing or not willing to remain in a healthcare facility during the study
2. Moribund condition (death likely in days) or not expected to survive for >7 days – due to other and non-COVID-19 related conditions
3. Participant on invasive mechanical ventilation via an endotracheal tube or ECMO, or high-flow oxygen
4. Participant not able to take medications by mouth (as capsules or as a powder, mixed in water).
5. Disallowed concomitant medication:
   - Consumption of any herbal products containing 20-hydroxyecdysone and derived from *Leuzea carthamoides*; *Cyanotis vaga* or *Cyanotis arachnoidea* is not allowed (e.g. performance enhancing agents)
6. Any known hypersensitivity to any of the ingredients, or excipients of the study medication, BIO101. Refer to section 6.2.2 for a list of excipients.
7. Renal disease requiring dialysis, or known renal insufficiency (eGFR≤30 ml/min/1.73 m^2, based on Cockcroft & Gault formula)
8. In France:
   - Non-affiliation to compulsory French social security scheme (beneficiary or right-holder)
   - Being under tutelage or legal guardianship

5.3 Withdrawal of Participants During the Study

The target treatment duration, with the study medication, is 28 days or until reaching a study event as defined in sections 3.3.1 and 7.1.1. Participants may withdraw from the study earlier if one of the following occurs:

- The participant wishes to stop their participation at any time during the study
- When the investigator thinks the participation should be stopped, due to safety or compliance reasons
- When one or more safety stopping rules are met – for more information about stopping rules, see the section 7.2

Participants who finish study participation before day 28 will undergo end-of-study assessments, similar to those who are taking place on the day 28 assessments, if possible. These participants will also have the 14 (± 2) and 60 (± 4) days, post-intervention phone calls.

The confidential information in this document is provided to you as an investigator, potential investigator or consultant for review by you, your staff and applicable Independent Ethics Committee or Review Board. It is understood that the information will not be disclosed to others without written authorization from Biophytis S.A.

6 Study Medication

The investigational product BIO101 is prepared in accordance with Good Manufacturing Practice (GMP) as required by Good Clinical Practice (GCP).

The active ingredient in BIO101 is 20E. it is found and extracted by purification from the plants of the Cyanotis species, including *Cyanotis arachnoidea C.B. Clarke* or *Cyanotis vaga Lour. (Shultes)*.

Nutra Green (China) is the raw material supplier that provides extracted 20E at a 90% purity. This raw material is further processed by Patheon (Germany) to obtain the active pharmaceutical ingredient of 20E at 97% purity. This is next formulated by Amatsi Avogadro (France) to the final capsule presentation (active pharmaceutical ingredient with additional excipients). Amatsi also follows the stability of the product, develop the drug substance and the drug product monograph, and ensures quality release and transportation of the product to the clinical centers under the sponsor responsibility.

Placebo was prepared in accordance with GMP as required by the current GCP and manufactured by Amatsi Avogadro as well.

A Certificate of Analysis (CoA) is included in the CMC IMPD (Chemistry, Manufacturing and Controls Investigational Medicinal Product Dossier).

6.1 Dosing and Administration

2 treatment arms will be tested in this study: 350 mg b.i.d. of BIO101 and placebo b.i.d. A rationale for the doses selected of the study medication is provided in section 4.2. All therapeutic units (TU), given as 350 mg BIO101 b.i.d. or placebo b.i.d., are identical in compliance with the double-blind process. Administered daily dose is the same throughout the whole treatment period.

Participants will receive the study medication for up to 28 days or until a clinical endpoint is reached (i.e., ‘negative’ or ‘positive’ event). Participants who are officially discharged from hospital care will no longer receive study medication.

Study participants are instructed by the investigator or the investigation center staff to start the treatment at the end of the baseline assessments and will start treatment immediately following these assessments.

Each administration is made of two capsules, to be taken orally, swallowed with water or fruit juice or other common soft drinks. Capsules should be taken twice a day: once in the morning and once in the evening. Those can be taken with a meal such as breakfast or dinner, approximately 12 hours apart (± 2 hours). If the participant forgets or is not able to take the capsules at the appointed time, he/she should be instructed not to make up for the missed pills. If the participant has a problem with swallowing the capsules, they can open the capsules and mix their content with water. Please note, that this will not cause a clear solution. The participant must make sure to drink the whole amount of fluid, in which the capsule content was mixed with.

6.1.1 Administration of First Dose

Participant will receive the first dose once they have been deemed as eligible, finished the baseline assessment, underwent the randomization procedure and had the blood draw for T0, of the pop-PK study (more about the pop-PK study in sections 3.3.4 and 7.3.6). This can occur, in the morning (am) or afternoon and evening hours (pm).

The confidential information in this document is provided to you as an investigator, potential investigator or consultant for review by you, your staff and applicable Independent Ethics Committee or Review Board. It is understood that the information will not be disclosed to others without written authorization from Biophytis S.A.

6.2 Supply Procedures

6.2.1 Accountability

In the US, all Tus will be distributed to the clinical centers by PCI Pharma Services (6166 Nancy Ridge Dr., San Diego, CA 92121, USA). PCI will ensure all logistical steps including repository, physical management of TU packages and TU delivery to investigation centers in the US.

In Europe, all Tus will be distributed to the clinical centers by Amatsi (17, parc des Vautes, 34980 Saint-Gely-du-Fesc, France). Amatsi will ensure all logistical steps including repository, physical management of TU packages and TU delivery to EU investigation centers.

The order to deliver the initial stock and further reorders of individual investigation centers will be given by the contract research organization (CRO), according to the center's actual enrollment rate.

Return of expired Tus or unused Tus will be ordered by Biophytis.

Detailed information on TU handling will be provided in specific procedures in the trial master file (TMF).

6.2.2 Formulation, Appearance, Packaging, and Labeling

BIO101 is formulated as a 251 mg capsule that includes 175 mg of the active principle ingredient, 20E, and also the following compendial excipients: colloidal silica, microcrystalline cellulose and magnesium stearate. Placebo is manufactured as an identical size 1 opaque capsule containing colloidal silica and microcrystalline cellulose.

Capsules containing BIO101 and placebo are opaque and identical in appearance. Stability was tested following ICH regulations. In order to be fully compliant, the drug substance and the excipients are controlled according to the FDA and European pharmacopeia.

The study medication is packaged in units containing sufficient capsules for a 1-week treatment (7 days). The study medication will be further packaged and labeled according to local law and regulatory requirements. All study medication has to be stored as specified at delivery and in the original packaging. In case of a deviation in storage conditions, the clinical site cannot dispense the affected study medication and have to notify Biophytis right away. Utmost care will be taken to correctly dispense the study medication as assigned by the randomization system.

Each weekly box, contains 28 capsules, which is presented in 2 X 2 columns. Each column can contain capsules of 175 mg of BIO101 or placebo. The capsule combinations correspond to daily doses of 350 mg b.i.d. or placebo.

As participants receive their treatment only while on the study facility, it is the department staff who will administer the medication each morning and each evening, which will comprise of 2 capsules at a time, and will supervise the participant during the process of swallowing the study medication.

Detailed information on study medication packaging and dispensing will be provided to the sites in a pharmacy manual and in the procedures in the TMF.

The confidential information in this document is provided to you as an investigator, potential investigator or consultant for review by you, your staff and applicable Independent Ethics Committee or Review Board. It is understood that the information will not be disclosed to others without written authorization from Biophytis S.A.

6.2.3 Storage and Stability

Based on the available stability data of the Drug Product, Biophytis currently certifies a shelf life of at least 36 months at room temperature and protection from light and humidity. For more details please also see the IMPD.

6.2.4 Preparation

All treatment packages are delivered ready to be given directly to the study participants. No further preparation is needed by the study Staff.

Study participants should be given 4 capsules per day, every day, 2 in the morning and 2 in the evening.

If a dosing administration is skipped, study participants should start a new row the day after and should not take the skipped capsules.

Important note: Date of each next visit should be calculated back on Day 0 visit (baseline) in order not to exceed the total duration of 28 days of treatment (capped exposure).

6.3 Measures to Reduce Bias

In part 1, randomization will be stratified by RAS pathway modulator use (Yes/No), and co-morbidities (none, or 1 and above).

In part 2, randomization will be stratified by center, gender, RAS pathway modulator use (Yes/No), co-morbidities (none, or 1 and above), receiving CPAP/BiPAP at study entry (Yes/No) and suspicion of COVID-19 related myocarditis or pericarditis (present or not).

Randomization is performed during the baseline visit for each study participant fulfilling all the inclusion and non-inclusion criteria, according to the appropriate ratio (see also section 8.4.2).

6.3.1 Record of Randomization and Unblinding

Each treatment kit will show a pre-printed kit number. The kit number will be assigned after a participant is qualified and is randomized, either via paper randomization list available on site or via IWRS. Neither the Investigator, the staff nor the Sponsor will be aware of the treatment that corresponds to the kit number. The assigned treatment cannot be retrieved unless a specified unblinding procedure is engaged by the investigator. Unblinding can only occur when it is deemed necessary by the responsible physician of the investigation center in the context of a severe or serious adverse event.

The randomization list will be provided by the CRO to the manufacturer and to the eCRF identified responsible person. The randomization list will not be available to the Sponsor staff, study participants, investigators, monitors or employees of the clinical site involved in the management of the study before unblinding of the data (after database lock), except in case of emergency.

The CRO team, that is performing the data management and statistical activities will receive a copy of the randomization list after database lock.

A specific unblinding procedure will be made available for the investigator to perform emergency unblinding of a given participant for documented reasons. An unblinding procedure can only be engaged in an emergency situation where the Investigator considers it essential to know what treatment the participant was receiving. The monitor shall be notified promptly if unblinding is performed. The

The confidential information in this document is provided to you as an investigator, potential investigator or consultant for review by you, your staff and applicable Independent Ethics Committee or Review Board. It is understood that the information will not be disclosed to others without written authorization from Biophytis S.A.

investigator will document the date, time, and reason for the unblinding in the participant's medical records.

Before availability of the IWRS system, i.e., while paper randomization is in operation, the site will have access to sealed emergency unblinding envelopes. Once the IWRS is available, the monitor will check the completeness and status of the envelopes and thereafter the monitor can retrieve the envelopes and have them destroyed.

Once the IWRS is available, emergency unblinding can occur through that system.

It is the investigator's responsibility to ensure that there is a procedure in place to allow access to the remote system in case of emergency.

The investigator will inform the participant how to contact his/her backup in case of emergencies when he/she is unavailable.

Should the code be (intentionally or unintentionally) broken by the Investigator or by a clinical staff, whether the participant could remain in the study or not should be judged by the Investigator according to the participant's best interest. The event will be immediately reported to the Sponsor and to SGS and dealt with as a major protocol deviation.

The participant's clinical data will not be analyzed in the per protocol set.

6.3.2 Dissemination of Results

Apart from the DMC, no personnel will be unblinded for study data before database lock of study part 2. The first interim analysis will be used to determine if the treatment is safe. The DMC recommendation, i.e. to continue or not with the study, will be made public. With regards to data outputs (tables) only the DMC will be unblinded.

On the event that during the second step of the first interim analysis an indication of activity of BIO101 will be identified, the DMC may decide to disseminate, if they deem that it is in the interest of public health.

All dissemination of data outputs will be documented. With the names of the individuals, date results provided, and extent of the information shared, will be tracked using a specific form.

Refer to the DMC charter for more details.

6.3.3 Potential for Operational Bias

Since neither investigators nor other individuals directly involved in the conduct of the study will have access to any results the potential to introduce operational bias is considered negligible.

6.4 Compliance

A participant study medication log will be completed (to be kept in the source documents) and recorded into the eCRF. An algorithm will automatically calculate the adherence based on visit dates.

The confidential information in this document is provided to you as an investigator, potential investigator or consultant for review by you, your staff and applicable Independent Ethics Committee or Review Board. It is understood that the information will not be disclosed to others without written authorization from Biophytis S.A.

6.5 Concomitant Medications

6.5.1 Disallowed Medications

The use of any herbal products containing 20-hydroxyecdysone and derived from *Leuzea carthamoides*; *Cyanotis vaga* or *Cyanotis arachnoidea* (e.g. performance enhancing agents) is not allowed during the study.

Starting a disallowed medication will not lead to termination of participation or study medication intake, but participant will not be included in the per-protocol cohort. More on study cohorts can be found in section 8.

6.5.2 Allowed Medications

The use of RAS pathway modulators (e.g., ACEi, ARB, or renin or aldosterone inhibitors) will be allowed. Other treatments to COVID-19, including antimalarial, antiviral or antimicrobial agents are allowed before or during the study.

6.6 Rescue Medications

There are no rescue medications in this study.

7 Study Assessments

7.1 Assessment of Efficacy

Efficacy is assessed, in this study, by 3 methods: record of events, status scales and biomarkers.

7.1.1 Record of Events

7.1.1.1 Negative and positive events

In this study, events are divided as 'negative' and 'positive'.

The negative events are:

- All-cause mortality
- Respiratory failure, defined as any of the following:
    - Requiring mechanical ventilation (including cases that will not be intubated due to resource restrictions and triage)
    - Requiring ECMO
    - Requiring high-flow oxygen

The 'positive' event is:

- official discharge from hospital care by the department due to improvement in participant condition (self-discharge by participant is not considered a positive event).

For the primary endpoint, events are recorded only once – i.e. the first event that occurs, is the one that leads to the end-of-study intervention.

The confidential information in this document is provided to you as an investigator, potential investigator or consultant for review by you, your staff and applicable Independent Ethics Committee or Review Board. It is understood that the information will not be disclosed to others without written authorization from Biophytis S.A.

7.1.1.2 CPAP/BiPAP events

CPAP/BiPAP events are defined as:

- Requiring CPAP/BiPAP in participants entering the study on low flow oxygen

7.1.2 Status Scales

In this study, two status scales are collected: the News2 and the APACHE II. All scales have been validated as predictors of future outcome. The News2 will be collected through all the study assessment, until the participant reaches an outcome and finishes participation in the study. The APACHE II will be collected at time points as indicted in the Schedule of Activities, at the investigator's discretion.

7.1.3 Biomarkers

Biomarkers in this study include: viral load (nasal/nasopharyngeal swab), and the predefined blood biomarkers. Blood biomarkers include: markers related to the RAS / MAS pathways, inflammatory markers and muscle specific markers. Biomarkers are collected during the study assessments as long as the participant is in the study.

The following parameters will be assessed by the local labs:

- Inflammatory markers: LDH, D-dimer, CRP, ferritin

The following parameters will be assessed by the central lab:

**Biochemistry**	PIIINP, myoglobin, bone specific alkaline phosphatase, amylase
**Skeletal-muscle proteins**	CK-MM, CK-MB, CK macro type I, CK macro type II
**Biomarkers related to BIO101 mechanism of action (RAS/ MAS panel)**	Ang 1-7, Ang 1-5, Ang 2, renin, aldosterone, and ACE
**Inflammatory biomarkers**	Troponin (I and T), IL-2, IL-6, IL-10, and TNFα
**Viral load (nasal/nasopharyngeal swab)**	
**Genotyping (at baseline only, optional, subjected to a separate consent)**	ACE2

7.1.4 Pulse Oximetry and Fraction of Inspired Oxygen

Peripheral arterial oxygen saturation (SpO_2) estimates the amount of oxygen in the blood, expressing the percentage of oxygenated hemoglobin compared to the total amount of hemoglobin It will be measured by pulse oximetry, an indirect and non-invasive method.

In addition, the fraction of inspired oxygen (FiO_2) and the oxygen delivery system (e.g., simple facial mask; partial rebreather mask; nasal cannula; C-PAP machine; …) are to be recorded. If FiO_2 cannot be read

The confidential information in this document is provided to you as an investigator, potential investigator or consultant for review by you, your staff and applicable Independent Ethics Committee or Review Board. It is understood that the information will not be disclosed to others without written authorization from Biophytis S.A.

directly from the device, the volume (in liters) and % of oxygen the system delivers (e.g., 100%) should be recorded instead

7.1.5 Timing of Efficacy Assessments

Study assessment take place in the study facility, during the daily activities of the facility. As discussed in section 4.3, there are 3 study periods: pre-intervention, on-intervention and post-intervention. Efficacy assessment take place at the end of the pre-intervention period (i.e. baseline) and then, in specific intervals during the on-intervention period, and post-intervention by phone call. For status scales and biomarkers, the on-intervention assessment is compared to the pre-intervention assessment.

For more information on study assessment, see the sections 1.3 and 4.3.

7.2 Safety Assessments

Safety is assessed by 3 methods: physical examination (including vital signs), safety labs and safety reporting.

For all safety assessments, where the assessments requested for the study are performed during routine care at the site, the results of such routine assessments can be used for the study and those assessments do not need to be repeated. However, the full panel of study assessments should be performed on the same study day (i.e., routine assessments and any additional non-routine study assessments on the same day).

7.2.1 Physical Examination

The physical examination will be done according to standard practices. It is done as part of all study assessments, except for the baseline assessment (which takes place immediately after the screening assessments, which includes a physical examination, are done).

7.2.1.1 Vital Signs

In addition, the physical examination includes assessments of all the following vital signs:

- Heart rate and blood pressure – will be taken in the following method:
    - Participant will lie supine for 5 minutes. At the end of this period, pulse and blood pressure are measured
    - Then participant stand up, with pulse and blood pressure measured 1 and 3 minutes after standing up. This maneuver is done in order to detect the presence of orthostatic hypotension – which will be recorded as an AESI (see more information in section 7.2.4.4)
        - If the participant cannot stand-up, only supine measures should be taken
- Body temperature
- Respiratory rate

7.2.2 Safety Labs

These include blood draws under fasting state for hematology and biochemistry panels and urinalysis. Safety labs should be done when the participant has fasted for at least 8 hours (water and tea are allowed).

The following parameters will be assessed by the local labs:

The confidential information in this document is provided to you as an investigator, potential investigator or consultant for review by you, your staff and applicable Independent Ethics Committee or Review Board. It is understood that the information will not be disclosed to others without written authorization from Biophytis S.A.

Table 7: Parameters that are analyzed at the local lab

**Biochemistry**	sodium, potassium, chloride, bicarbonate, calcium, urea, uric acid creatinine, albumin, glucose, ammonia, aspartate aminotransferase (AST), alanine aminotransferase (ALT), alkaline phosphatase, gamma-glutamyl aminotransferase, bilirubin (total, indirect, direct), creatine phosphokinase CK, lactate dehydrogenase, total protein, lipase, total cholesterol, LDL, LDL-cholesterol measured (Reflex), HDL, triglycerides, fibrinogen, and eGFR;
**Hematology**	hemoglobin, hematocrit, red blood cell (RBC) count, white blood cell (WBC) count with differential, platelet count, mean corpuscular hemoglobin, mean corpuscular hemoglobin concentration, and mean corpuscular volume
**Coagulation**	activated partial thromboplastin time, prothrombin time, and international normalized ratio
**Arterial-blood gasses**	PaO_2, $PaCO_2$, pH, HCO_3, SaO_2
**Inflammatory biomarkers**	LDH, D-dimer, CRP, ferritin
**Testicular biomarkers (for male participants)**	testosterone, LH, FSH
**Pregnancy test (urine, at screening only, for female participants who are not 5 years into menopause)**	β-HCG

A midstream urine sample will be collected for urinalysis by dipstick for specific gravity abnormal, pH, glucose, protein, blood, ketones, bilirubin, urobilinogen, nitrite and leukocyte. If dipstick urinalysis is abnormal, microscopic examination for WBC, RBC, epithelial cell, crystals, bacteria and casts will be performed.

The following parameters will be assessed by the central lab:

Table 8: Parameters that are analyzed at the central lab

Chemistry	PIIINP, myoglobin, bone specific alkaline phosphatase, amylase
Skeletal muscle proteins	CK-MM, CK-BB, CK-MB, CK Macro type I and II

The confidential information in this document is provided to you as an investigator, potential investigator or consultant for review by you, your staff and applicable Independent Ethics Committee or Review Board. It is understood that the information will not be disclosed to others without written authorization from Biophytis S.A.

Biomarkers related to BIO101 mechanism of action (RAS/MAS panel)	Ang 1-7, Ang 1-5, Ang 2, renin, aldosterone, and ACE
Inflammatory markers	Troponin (I and T), IL-2, IL-6, IL-10, and TNFα
Testicular biomarkers for male participants	inhibin B

7.2.3 Electrocardiogram (ECG)

This is done, during all on-intervention assessments.

7.2.4 Safety Reporting

Safety events are solicited during study assessments and can come also as unsolicited reports out of the routine study assessments. Any such report will be recorded as an AE, SAE and AESI.

7.2.4.1 Adverse Events

An AE means any untoward medical occurrence associated with the use of a study medication in humans, whether or not considered study-medication-related.

7.2.4.2 Classification of Adverse Events

7.2.4.2.1 Intensity

Adverse events (including abnormal laboratory findings) severity will be rated according to the Common Terminology Criteria for Adverse Events (CTCAE) Version 5.0:

The confidential information in this document is provided to you as an investigator, potential investigator or consultant for review by you, your staff and applicable Independent Ethics Committee or Review Board. It is understood that the information will not be disclosed to others without written authorization from Biophytis S.A.

Table 9: Severity assessment, according to CTCAE

Grade	Severity	description
1	Mild	• Asymptomatic or mild symptoms<br>• Clinical or diagnostic observations only<br>• Intervention not indicated
2	Moderate	• Minimal, local or noninvasive intervention indicated<br>• Limiting age appropriate instrumental ADL (activities of daily living)
3	Severe	• Medically significant but not immediately life-threatening<br>• Hospitalization or prolongation of hospitalization indicated<br>• Disabling or limiting self-care ADL
4	Life-threatening	• Potential for immediate life-threatening consequences<br>• Urgent intervention indicated
5	Death	

Of note, the term “severe” does not necessarily equate to “serious”

7.2.4.2.2 Assessment of Causality and Relations to Study Medication

All AEs must have their relationship to the study medication assessed by the clinician who examines and evaluates the study participant based on the temporal relationship between the occurrence of the AE and the study medication. It is based on clinical judgment.

Causality is assessed according to the following parameters: pharmacological plausibility (i.e. that the suspected agent can cause the event, due to its mechanism of action), the participant health condition (i.e. that the health state and concomitant medical conditions the participant is known to have could cause the event) and by applying the methods of de-challenge (i.e. that the event improves after stopping the suspected agent) and re-challenge (i.e. that the event returns or worsens after re-introduction of the suspected agent).

The degree of certainty about any causality, when it comes to the study medication, will be assessed and reported according to the WHO standards[58]:

Table 10: Causality assessment, according to WHO

**Certain**	• An event or abnormal laboratory test results, with a plausible time relationship to drug intake<br>• Cannot be explained by disease or other drugs<br>• A positive de-challenge (i.e. improvement after stopping study medications

	• A positive re-challenge (i.e. return or worsening of event upon restarting study medication) – in the case it is recorded<br>• A plausible pharmacological and pathological link to study medication exists<br>• Event is definitive pharmacologically or phenomenologically (i.e. an objective and specific medical disorder or a recognized pharmacological phenomenon)
**Probable / Likely**	• An event or abnormal laboratory test results, with a plausible time relationship to drug intake<br>• Unlikely to be attributed to disease or other drugs<br>• Could also be explained by disease or other drugs<br>• Information on drug withdrawal or renewal may be lacking or unclear
**Unlikely**	• An event or abnormal laboratory test results, without a plausible time relationship to drug intake<br>• Disease or other drugs provide plausible explanations
**Conditional / Unclassified**	• More data for proper assessment needed, or additional data is under examination
**Un-assessable / Unclassifiable**	• Cannot be judged because information is insufficient or contradictory<br>• Data cannot be supplemented or verified

7.2.4.2.3 Assessment of Expectedness

Event, for which the causality assessment in relation to study medication is 'certain', "probable' or 'likely', will have to be assessed for expectedness, based on the following:

- In relation to BIO101, no events are known to be expected, however, the following event is included in the investigator's brochure (IB) or study medication label: Abuse, misuse or diversion. . See more information in section 2.4

7.2.4.2.4 Assessment of Event Status

Status of events will be assessed as one of the following:

- Initiated – between event occurrence and until the initial report was issued to the study sponsor
- Ongoing, worsening – when the clinical state of the event has become worse, compared to the initial report
- Ongoing – when the event is still occurring and the clinical state is not worse, nor is it better, compared to the initial report
- Ongoing, improved – when the event is still occurring and the clinical state is better, compared to the initial report
- Resolved – when the event is over, the health status of the participant returns to what it was prior to the event and no long-term sequel is noted

The confidential information in this document is provided to you as an investigator, potential investigator or consultant for review by you, your staff and applicable Independent Ethics Committee or Review Board. It is understood that the information will not be disclosed to others without written authorization from Biophytis S.A.

- Resolved with sequel – when the event is over or considered as not likely to change, the health status of the participant has or hasn't returned to what it was prior to the event or long-term sequel is noted

7.2.4.3 Serious Adverse Events (SAE)

An AE is considered "serious" if, it results in any of the following outcomes:

- Death
- Life-threatening
- Causing inpatient hospitalization or prolongation of existing hospitalization
- Any persistent or significant incapacity or substantial disruption of the ability to conduct normal life functions, or a congenital anomaly/birth defect
- Important medical events that may not result in death, be life-threatening, or require hospitalization may be considered serious when, based upon appropriate medical judgment, they may jeopardize the participant and may require medical or surgical intervention to prevent one of the outcomes listed in this definition. Examples of such medical events include allergic bronchospasm requiring intensive treatment in an emergency room or at home, blood dyscrasias or convulsions that do not result in inpatient hospitalization, or the development of drug dependency or drug abuse

Certain SAE are considered with a heightened level of urgency. These include:

- SAE resulting in death of, or is life-threatening to a participant, regardless of causality
- When an SAE causality, with relation to study medication, is assessed as certain, probable or likely, and the event is not considered as 'expected', with relation to study medication (SUSAR; for reporting see Section 7.2.7)

7.2.4.4 Adverse Events of Special Interest (AESI)

These adverse events will be reported as an SAE. More about reporting in section 7.2.7. In this study, the following is considered as AESI:

- Documented events of orthostatic hypotension (OTH), with supine and standing blood pressure measurements

7.2.5 Unanticipated Events Related to Study Procedures, but not to Study Medication

Unanticipated events occur, when study procedures, other than the study medication, invoke risks to participants or others to include, in general, any incident, experience, or outcome that meets all of the following criteria:

- Unanticipated in terms of nature, severity, or frequency given:
  - The research procedures that are described in the protocol-related documents, such as the IEC/IRB-approved research protocol and informed consent document
  - The characteristics of the participant population being studied
- The event is related or possibly related to participation in the research ("possibly related" means there is a reasonable possibility that the incident, experience, or outcome may have been caused by the procedures involved in the research)

The confidential information in this document is provided to you as an investigator, potential investigator or consultant for review by you, your staff and applicable Independent Ethics Committee or Review Board. It is understood that the information will not be disclosed to others without written authorization from Biophytis S.A.

- The suggestion that the research places participants or others at a greater risk of harm (including physical, psychological, economic, or social harm) than was previously known or recognized

7.2.6 Follow-up on Adverse Events

The occurrence of an AE or SAE may come to the attention of study personnel during study visits, during interviews of a study participant presenting for medical care, or upon review by a study monitor. All AEs including local and systemic reactions not meeting the criteria for SAEs will be captured on the appropriate report form. Information to be collected includes event description, time of onset, clinician's assessment of severity, relationship to study product (assessed only by the investigator or treating physician), and time of resolution/stabilization of the event. All AEs occurring while on study must be documented appropriately regardless of relationship. All AEs will be followed to adequate resolution.

An SAE follow-up includes a prompt blood sampling for PK analyses, if deemed applicable by the Investigator or by his/her staff. Any medical condition that is present at the time that the study participant is screened will be considered as baseline and not reported as an AE. However, if the study participant's condition deteriorates while performing any physical assessments at any time after signing the ICF (including the screening period), it will be recorded as an AE. Unanticipated problems (Ups) will be recorded in the data collection system throughout the study.

Changes in the severity of an AE will be documented to allow an assessment of the duration of the event at each level of severity. AEs characterized as intermittent require documentation of onset and duration of each episode.

The PI will record all reportable events with start dates occurring any time after informed consent is obtained until the last day of study participation. At each study visit, the investigator will inquire about the occurrence of AEs/since the last visit. Events will be followed for outcome information until resolution or stabilization.

7.2.7 Adverse Events Reporting

All adverse events are to be followed-up, until they reach the state of resolved (without or with sequel). Adverse events are summarized and routinely reviewed by the DMC and are reported as part of the standard IND updates and drug-safety update reports (DSUR).

In case of a death and life-threatening event the investigator needs to notify the sponsor and CRO immediately upon awareness.

SAE, AESI and unanticipated events related to study procedures, require specific reporting, as follows:

- They must be promptly reported (within 24 hours) to the study sponsor and the CRO
- The report must include, besides the standard assessment, a record of cause for seriousness or that it is an AESI
- A narrative describing the event must be included
- All physical, laboratory and other types of testing should be described and final reports of these testing must be included as soon as they are available
- The study CRO will establish a special process for these reports, which will be included in the TMF

SUSAR and other expedited reporting will be conducted according to the ICH guidelines and regulatory requirements in the relevant countries.

The confidential information in this document is provided to you as an investigator, potential investigator or consultant for review by you, your staff and applicable Independent Ethics Committee or Review Board. It is understood that the information will not be disclosed to others without written authorization from Biophytis S.A.

Refer to the DMC charter for more details.

7.2.8 Safety Stopping Rules

The following sections will describe stopping rules for individual study participants and for the whole study.

7.2.8.1 Safety Stopping Criteria for Individual Participants

Stopping a participation due to safety, is done, first and foremost, based on clinical judgement of the study investigator. However, there are cases, in which stopping is mandatory. Intake of study medication will be stopped if one or more of the following occurs:

7.2.8.1.1 Adverse Event Stopping Criteria

- Any AE of at least grade 3 (i.e., severe) intensity and any SAE with a causality assessment of ‘certain’, ‘probable’ or ‘likely’
- A suspected allergic reaction to study medication with a grade 3 or higher rash or Grade 2 rash with evidence of systemic involvement

7.2.8.1.2 Liver Function Tests Stopping Criteria

Any of the following, in the absence of evidence of severe sepsis, septic shock or multiple organ failure:

- Aspartate aminotransferase (AST) and alanine aminotransferase (ALT):
    - In individuals with normal levels of creatine kinase (CK) at the relevant measurement:
        - When at baseline are within the normal limits – an increase of ≥5×ULN
        - When at baseline are above the normal limit – an increase of ≥2 times when compared to the baseline value AND ≥8×ULN (whichever is smaller)
    - In individuals with abnormal levels of CK:
        - Will not be used as parameters for stopping rules
- GGT increase to ≥2 compared to baseline or ≥x5 ULN
- An increase in ammonia levels to above the ULN – when there is no other cause for this increase
- Any evidence of abnormalities in liver synthetic functions, in the absence of a systemic hypercoagulability state, sepsis, septic shock or multi-organ failure:
    - Fibrinogen levels: to below the LLN
    - Coagulation function tests: international normalized ratio (INR) to ≥2×ULN
- An increase of total bilirubin level by ≥2 times, compared to baseline or to ≥5×ULN (whichever is lower)

7.2.8.1.3 Vital Sign Stopping Criteria

Any of the following, in the absence of evidence to severe sepsis, septic shock or multiorgan failure:

- Symptomatic hypotension (systolic blood pressure [SBP] <90 mm Hg). If hypotension is observed during the study, then a minimum of 2 repeat of blood pressure measurements will be obtained over a brief period. The mean of the 3 SBP measurements will be used to determine stopping criteria
- Tachycardia defined as heart rate >120 beats per minute (bpm) lasting longer than 30 minutes or with impaired consciousness

The confidential information in this document is provided to you as an investigator, potential investigator or consultant for review by you, your staff and applicable Independent Ethics Committee or Review Board. It is understood that the information will not be disclosed to others without written authorization from Biophytis S.A.

  - In case there is also worsening hypoxemia, it should be ruled out that the tachycardia is a response to hypoxemia (i.e. that this is not a manifestation of worsening respiratory deterioration, due to COVID-19)

7.2.8.1.4 Electrocardiogram (ECG) Stopping Criteria:

Any of the following, in the absence of evidence to severe sepsis, septic shock or multiple organ failure:

- A change in the average QT corrected using Fridericia's correction (QTcF) interval ≥500 msec from 3 or more ECG tracings separated by at least 5 minutes (If QTcF ≥500 msec is noted on a scheduled or unscheduled ECG, then 2 additional ECGs will be obtained within 5 minutes to confirm the abnormality. The mean of the 3 ECGs will be used to determine continued eligibility), which is
  - ≥60 msec
  - Sustained (lasting ≥30 seconds)

7.2.8.1.5 Renal Failure Stopping Criteria

At the following, it is up to the investigator to decide whether to stop the study treatment:

- eGFR ≤30 mL/min/1.73 m^2, based on Cockcroft & Gault formula

OR

- Participant requires renal dialysis

In case the investigator decides it is warranted to continue treatment, the event is not considered to be a stopping criterion and does not have to be reported as such.

7.2.8.1.6 Stopping Due to a Medical Procedure

Participants may stop the study medication due to a medical procedure (e.g. fasting before general anesthesia).

7.2.8.1.7 Participant Non-compliance

Participation in the study may be stopped due to severe non-compliance with study medication and/or procedures. The definition of 'severe' is depending on the judgement of the study investigator and taking into account the benefit / risk assessment, under such non-compliance.

7.2.8.1.8 Resuming Study Medication after Stopping

Except for cases of grade 3 allergic reactions, and if according to the clinical judgement of the study investigator there is a reason to believe that the benefit of resuming treatment outweighs the risks, the study participant can resume study medication, if the following criteria are met:

- When stopping occurred due to an adverse event
  - If event is suspected as related to study medication, resume only if not life-threatening and full resolution has occurred. If event recurs, do not resume again.
  - If event is not related to study medication, resume when the event has improved to a level that is considered clinically stable
- When stopping due to liver toxicity:
  - Resume when the signs of toxicity are alleviated (i.e. the relevant LFT are back to those observed at baseline). If recurring after resuming, do not resume again

The confidential information in this document is provided to you as an investigator, potential investigator or consultant for review by you, your staff and applicable Independent Ethics Committee or Review Board. It is understood that the information will not be disclosed to others without written authorization from Biophytis S.A.

- When stopping due to cardiac toxicity:
  - If ECG changes are seen, resume after ECG normalization is observed, in at least 2 recording that are 24 hours apart. If abnormality returns, do not resume again
  - If hypotension or tachycardia is seen, consider adjusting the dosage of antihypertensives, if they are used. Resume only after 24 hours and normalization of blood pressure / pulse measurements are achieved in 3 measures.
- In case of stopping due to a medical procedure, resume as soon as it is safe to restart oral medication.
- In case of stopping due to severe non-compliance, resume only after it is determined that participant can comply with medication requirements

7.2.9 Safety Stopping Rules for Entire Study

The DMC will be forwarded any of the following events:

- Any SAE
- Any death that is, at least, possibly related to the study medication
- Any grade 4 adverse event that is, at least, possibly related to the study medication
- Any event that meets any of the individual stopping rules (see section 7.2.8), which is at least possibly related to the study medication

According to the DMC charter, the DMC has the possibility to request an ad hoc meeting based on the data they receive with these events. After review and based on clinical judgment taking into consideration participant safety, the DMC will make the recommendation to the Sponsor to

- Continue the study as is
- Adjust the study
- Terminate the study

In addition, fixed DMC meetings are foreseen, i.e.,

- For Part 1, whenever a group of 5 participants has completed the 14-day (± 2) follow-up phone call, for review of eCRF data;
- After the last participant of part 1 has reached day 14;
- For part 2, every 6 weeks..

The objective of the DMC will be to review all (unblinded) safety data (including the overall number of participants treated up to that point, rates, and participant-level details). The DMC will hereby also determine if there is an imbalance in the treatment arms with respect to these events, based on clinical judgment of the DMC and make a recommendation to the sponsor to temporarily hold, modify, continue or permanently stop the trial after every meeting.

Additional criteria for discontinuation of specific sites or of the study as a whole are described in Section 9.1.2.

Refer to the DMC charter for more details.

The confidential information in this document is provided to you as an investigator, potential investigator or consultant for review by you, your staff and applicable Independent Ethics Committee or Review Board. It is understood that the information will not be disclosed to others without written authorization from Biophytis S.A.

7.3 Additional Assessments

These assessments may inform eligibility for participation and be used as measures in pre- and post-hoc analysis of the study data.

7.3.1 Medical History

The medical history is recorded during the pre-intervention period in order to assess the eligibility for participation and to be used in pre-hoc and post-hoc analyses of the study data.

A specific note should be made to the participant record, if there is suspicion of the existence of COVID-19 related myocarditis or pericarditis, as this is a criterion for stratification.

7.3.2 Concomitant Medications

At each study assessment, the concomitant medications are recorded. During the pre-intervention period, it is done to assess eligibility and during the study period, it is done to assess compliance with the protocol. See more information on disallowed medication in section 6.5.

7.3.3 Anthropometry

These assessments include weight and height. They are measured as part of the baseline assessments and will be used for further pre-and post-hoc analyses of the study data.

7.3.4 Pregnancy Testing

Due to lack of sufficient reproductive toxicity data, pregnant women will not be able to participate in this study. Women who are less than 5 years into menopause will undergo a urine pregnancy test (β-HCG) to rule out pregnancy state, this test will take place as part of the screening assessments.

7.3.5 Genetic Assessments

If the participant has consented to this assessment, blood will be drawn at baseline to assess ACE2 genetic polymorphism. This could be used as part of the post-hoc analyses of the study data.

7.3.6 Population PK

The procedure for this assessment is described in section 3.3.4

8 Statistics

8.1 Study Cohorts

8.1.1 Intent to Treat (ITT)

All participants who are randomized into the study and who received at least one dose of study medication will be included in the ITT cohort. This analysis set will be used as:

- primary efficacy analysis set for the interim and the final analysis.

8.1.2 Safety Population (SafP)

All participants included in the ITT cohort and who have at least one post-baseline safety assessment. This cohort will be used for the primary safety analysis.

The confidential information in this document is provided to you as an investigator, potential investigator or consultant for review by you, your staff and applicable Independent Ethics Committee or Review Board. It is understood that the information will not be disclosed to others without written authorization from Biophytis S.A.

8.1.3 Per-Protocol (PP)

All the participants included in the ITT cohort, who do not have a record of major protocol deviations, will be included in this cohort. It will be used for sensitivity analysis of primary and secondary efficacy endpoints.

8.2 Protocol Deviations

Protocol deviations will be clearly recorded in the CRF. A justification, if possible, should be made, to the existence of such a deviation. The purpose of this recording is to help in assessment of the data quality, introduce measures for improvement of data quality when needed and to be used by regulatory authorities and scientific journals, to make an opinion on the validity and robustness of the study findings.

8.2.1 Major Protocol Deviations

In this study, the following will be considered as major protocol deviations:

- Inclusion into the study with a breach of an inclusion or exclusion criterion
- Non-compliance resulting in missing more than 50% of study medication
- An interruption of treatment continuity of more than 7 days

8.3 Sample Size Justification

The sample size was calculated for a trial with 80% power and two-sided alpha of 0.05.

The relative frequency of the primary endpoint 'proportion of participants with a negative event' is expected to be 40% and 25% in the control and experimental arm, respectively (an absolute difference of 15%). The design has a (second) interim analysis at 50% information fraction and uses the O'Brien-Fleming-type cumulative error spending function boundaries for efficacy (Table 11). Given a 1:1 randomization ratio, the required sample size is estimated to be 310 participants in total (155 active : 155 placebo).

The efficacy boundary at the second interim analysis was set at a difference (experimental - control) of -22%. A difference of -22% means that the proportion of participant with a negative event is 22% higher in the control arm. The futility boundary at the interim analysis was set to 0%.

The stopping probabilities were based on 100.000 simulations at information faction 50% (i.e. 78 participants per treatment group). The probability of stopping for futility was calculated as the proportion of simulations with a proportional difference greater than 0%; and the probability for stopping for overwhelming efficacy was calculated as the proportion of simulations with proportional difference less than -22%

Assuming the null hypothesis holds (no treatment effect), the trial would be stopped for futility with a probability of 46.7% and the probability of obtaining overwhelming efficacy would be low (0.2%).If the treatment is harmful (5% more negative events in treatment arm), the probability of stopping for futility would be 71.1% and the probability of obtaining overwhelming efficacy would again be low (0.03%). If the treatment is effective (assuming the alternative hypothesis) the probability of stopping for futility would be low (2%) and the probability of obtaining overwhelming efficacy would be 15.8%.

The confidential information in this document is provided to you as an investigator, potential investigator or consultant for review by you, your staff and applicable Independent Ethics Committee or Review Board. It is understood that the information will not be disclosed to others without written authorization from Biophytis S.A.

Table 11: Sample Size Considerations

Assumed Trt Effect (Placebo-Active)	Futility		Efficacy			
	Boundary	Stopping Probability	Boundary	Harmful Effect	No Stopping	Stopping for Efficacy
40-25 (Alt)	0%	1.8%	-22% - 22%	0%	84.2%	15.8%
40-40 (Null)	0%	46.7%	-22% - 22%	0.2%	99.6%	0.2%
40-45 (Harm)	0%	71.1%	-22% - 22%	1.4%	98.6%	0.03%

The efficacy boundary is implemented with sole function of allocating a small alpha (0.0015) for the second interim analysis with sample size re-assessment. The study will continue to full completion regardless of whether primary endpoint reaches significance at the interim analysis.

Note that the futility boundary is conceived as non-binding allowing the DMC (refer to Section 7.2.9 for more details) to decide independently at the timing of the interim analysis, taking all available data into account, whether the study should continue or stop. This is especially important for a COVID-19 trial as by the time the interim analyses will take place, more precise information on the natural history of the disease and on the treatment-effect achieved by other compounds will be available. It will be important to take all the available information on Covid-19 at the time of interim into account to decide on the desirability of continuing the trial.

8.4 Description of Statistical Methods

8.4.1 General Analytical Considerations

8.4.1.1 Enrollment and Sample Size Re-assessment

The study will proceed in three stages. A first interim analysis will be performed when approximately 50 participants are expected to be evaluable for the primary endpoint, proportion of participants with a negative event at day 28. The DMC will evaluate possible futility of the trial based on the safety and tolerability of BIO101. In addition to this, both the primary endpoint results and a selection of parameters and biomarkers will be analyzed to provide preliminary information on the activity of BIO101. This will allow the IDMC to not only base their recommendation on safety but also have some insight on risk/benefit balance of participants. The study will continue unless the DMC advises to stop the study for futility.

When approximately 155 participants are evaluable for the primary endpoint, a second interim analysis is performed. At this point, the study might be halted for futility. In addition, the sample size may be increased to the maximum of 465, based on the conditional power calculation which will be performed at interim analysis, to ensure 80% power to detect a difference in proportion of negative events at day 28 at the final analysis. The study continues without adaptations otherwise. The results of the interim analysis will be assessed independently of the sponsor. In this way, operational bias will be minimized, even though

The confidential information in this document is provided to you as an investigator, potential investigator or consultant for review by you, your staff and applicable Independent Ethics Committee or Review Board. It is understood that the information will not be disclosed to others without written authorization from Biophytis S.A.

the sample size increase itself is informative about the likely range of the treatment effect. For this reason, investigators will not be made aware of the sample size increase. The study will continue to collect survival and safety data, as described in section 7.2.

An interim efficacy boundary will be determined using O'Brien-Fleming-type Lan-Demets spending functions for efficacy, with the sole function of allocating a small alpha (0.0015) for the interim analysis with sample size re-assessment. The study will continue to full completion regardless of whether the primary endpoint reaches significance at the interim analysis. The futility boundary is non-binding, allowing the DMC to provide independent advice at the time of both interim analyses.

The O' Brian Fleming type cumulative error spending function was used with the purpose of spending few alpha to interim analyses. A simulation adding an interim assessment at 16% information fraction (end of part 1 safety analysis) did not yield a difference in the alpha spending.

8.4.1.2 Other Items

Unless specified otherwise, computations will not impute data for missing values.

Missing data in time-to-event will be censored at date of last visit unless specified otherwise.

8.4.2 Method of Assigning Participants to Treatment Groups

Block-permuted randomization will be used to assign eligible participants in a 1:1 ratio. In part 1, randomization will be stratified for RAS pathway modulator use and co-morbidities (none vs. 1 and above). In Part 2, randomization will be stratified for center, gender, RAS pathway modulator use, co-morbidities (none vs. 1 and above), receiving CPAP/BiPAP at study entry (Yes/No) and suspicion of COVID-19 related myocarditis or pericarditis (present or not).

8.4.3 Analysis of Demographic and Other Baseline Characteristics

Descriptive statistics with respect to participant characteristics at baseline will be displayed for the ITT and the safety analysis set, both by treatment group and overall. A summary of key demographic data and also a listing presenting demographic and baseline data per participant will be presented.

8.4.4 General Analytical Considerations for Efficacy and Safety Analyses

Details of the planned analyses will be described in an SAP. Any deviations from the SAP will be justified in the clinical study report.

Descriptive statistics will be provided for selected demographics and safety by treatment and time as appropriate. Descriptive statistics on continuous data will include means, medians, standard deviations, and ranges, while categorical data will be summarized using frequency counts and percentages. Graphical summaries of the data may also be presented.

Two-sided tests will be used at a significance level equal to 0.05. Two-sided confidence intervals (CI) will be computed for a coverage probability of 0.95.

Binary outcomes will be described by proportions by treatment arm and compared with a Cochran-Mantel-Haenszel (CMH) test stratified by RAS pathway modulator use (Yes/No), gender, co-morbidities (none vs. 1 and above), receiving CPAP/BiPAP at study entry (Yes/No) and suspicion of COVID-19 related myocarditis or pericarditis (present or not).

The confidential information in this document is provided to you as an investigator, potential investigator or consultant for review by you, your staff and applicable Independent Ethics Committee or Review Board. It is understood that the information will not be disclosed to others without written authorization from Biophytis S.A.

Time to event outcomes ("survival times") will be described by treatment arm using the Kaplan-Meier method. Participants who have not had the event of interest at the time of the analysis will be censored at the time of the last assessment/measurement. Summary statistics will be provided by treatment arm in terms of the number of events, median and 95% CI and survival probabilities at specific time points. Survival curves will be plotted by treatment arm and compared with a log-rank test stratified by RAS pathway modulator use (Yes/No), gender, co-morbidities, receiving CPAP/BiPAP at study entry (Yes/No) and suspicion of COVID-19 related myocarditis or pericarditis (present or not). A stratified Cox regression model will be used to estimate the hazard ratio and its 95% CI, as well as to adjust the comparison for baseline covariates.

Additional details on analysis of all endpoints will be provided in the SAP.

8.4.5 Analysis of Primary and Key Secondary Endpoints and Sequential Testing

Statistical testing will be performed in a fixed sequence. Only in the case that the primary endpoint is significant at the primary analysis of the final analysis, will the key secondary endpoints be tested in the following hierarchy:

- Proportion of participants with events of respiratory failure at Day 28
- Proportion of participants with events of all-cause mortality at Day 28
- Proportion of participants with 'positive' events at day 28.

8.4.5.1 Analysis of Proportion of Participants with Negative Events During the 28 days of the Study (Primary Endpoint for the Study)

The proportion of participants with 'negative events' at Day 28 is the primary study endpoint. The primary analysis of the primary endpoint will be performed using a CMH test, stratified by RAS pathway modulator use (Yes/No), gender, co-morbidities, receiving CPAP/BiPAP at study entry (Yes/No) and suspicion of COVID-19 related myocarditis or pericarditis (present or not), on the ITT analysis set. Reporting will be done in terms of the risk difference and associated CI and p-value. Sensitivity analyses will be performed on the PP analysis set. Other sensitivity and or subgroup analyses may be included depending on new information that becomes available during the trial conduct (e.g. use of Dexamethasone). Full details will be included in the SAP. Participants with a missing outcome will be considered to have experienced a negative event. Note that given the severity of disease in the study population and short follow-up time for the primary endpoint (28 days), only a small number of participants with missing primary outcomes are anticipated.

8.4.5.2 Analysis of Proportion of Participants with Positive Events at day 28 (Key Secondary Endpoint Part 2)

The proportion of participants who reached a 'positive event' during the study is a key secondary study endpoint. The analysis will be performed using a CMH test, stratified by RAS pathway modulator use (Yes/No), gender, co-morbidities, receiving CPAP/BiPAP at study entry (Yes/No) and suspicion of COVID-19 related myocarditis or pericarditis (present or not), on the ITT analysis set. Reporting will be done in terms of the risk difference and associated CI and p-value. Sensitivity analyses will be performed on the PP analysis set. Full details will be included in the SAP.

The confidential information in this document is provided to you as an investigator, potential investigator or consultant for review by you, your staff and applicable Independent Ethics Committee or Review Board. It is understood that the information will not be disclosed to others without written authorization from Biophytis S.A.

Participants with a missing outcome will be considered to have experienced a negative event, or a non-responder for the positive event. Note that given the severity of disease in the study population and short follow-up time for the key secondary endpoint (28 days), only a small number of participants with missing outcomes are anticipated.

8.4.5.3 Analysis of Proportion of Participants with events of all-cause mortality at Day 28 (Key Secondary Endpoint Part 2)

The proportion of participants with events of all-cause mortality at Day 28 is a key secondary study endpoint. The analysis will be performed using a CMH test, stratified by RAS pathway modulator use (Yes/No), gender, co-morbidities, receiving CPAP/BiPAP at study entry (Yes/No) and suspicion of COVID-19 related myocarditis or pericarditis (present or not), on the ITT analysis set. Reporting will be done in terms of the risk difference and associated CI and p-value. Sensitivity analyses will be performed on the PP analysis set. Full details will be included in the SAP.

Participants with a missing outcome will be considered to have experienced an event of all-cause mortality, or a responder for this endpoint. Note that given the severity of disease in the study population and short follow-up time for the key secondary endpoint (28 days), only a small number of participants with missing outcomes are anticipated.

8.4.5.4 Analysis of Proportion of Participants with Respiratory Failure at Day 28 (Key Secondary Endpoint Part 2)

The proportion of participants experiencing a respiratory failure at Day 28 is a key secondary study endpoint. The analysis will be performed using a CMH test, stratified by RAS pathway modulator use (Yes/No), gender, co-morbidities, receiving CPAP/BiPAP at study entry (Yes/No) and suspicion of COVID-19 related myocarditis or pericarditis (present or not), on the ITT analysis set. Reporting will be done in terms of the risk difference and associated CI and p-value. Sensitivity analyses will be performed on the PP analysis set. Full details will be included in the SAP.

Participants with a missing outcome will be considered to have experienced a respiratory failure, or a responder for this endpoint. Note that given the severity of disease in the study population and short follow-up time for the key secondary endpoint (28 days), only a small number of participants with missing outcomes are anticipated.

8.4.6 Handling of Missing Data

Given that the primary and key secondary endpoints are assessed during the period of 28 days and the severe condition of the study population, none or only very limited missing data is anticipated. For the ITT cohort, in case an outcome is missing, the analysis will be performed by imputing a conservative outcome. For the proportion of participants with a negative event or experiencing respiratory failure, missing data on reaching a negative event or not will be imputed as reaching a negative event. For the proportion of participants with a positive event, missing information about reaching or not reaching a positive event (in participants who did not reach a negative event) will be imputed as not reaching a positive event. Further missing data handling and sensitivity analyses will be described in the SAP.

8.4.7 Sample Size Re-assessment at Interim Analysis

A decision whether to enroll more participants will be taken at the time of the second interim analysis

The confidential information in this document is provided to you as an investigator, potential investigator or consultant for review by you, your staff and applicable Independent Ethics Committee or Review Board. It is understood that the information will not be disclosed to others without written authorization from Biophytis S.A.

In case "promising" results are obtained, additional participant enrollment will be committed to the study if needed. This will be done based on the 'conditional power' of the study for the primary endpoint. Conditional power is the probability that the study will show statistical significance at primary analysis given the observed difference at interim and assuming this difference continues to its planned end with a constant effect size (the 'true' underlying difference in proportion of participants with negative events). The maximal sample size is set to a 50% increase of the initial size of 310, to a total of up to 465 participants. Note that with these numbers, the study would have 80% power to detect a difference in proportion of participants with negative events of 12% (40% versus 28%).

Following Mehta and Pocock (Mehta, 2011)[59] sample sizes will only be increased in case the interim results are promising, in which case the overall type-I error is not inflated by use of the conventional Wald statistic. More specifically, this 'promising zone' is defined in terms of the conditional probability of rejecting the null-hypothesis at primary analysis, given the estimated performance difference at interim ('conditional power') and assuming that this estimated difference is the true underlying difference. The promising zone ranges from a conditional power of 0 41 to 0.80. If the conditional power is below 0.41, the probability for a successful trial even after sample size re-assessment are considered too low to warrant the sample size increase ('unfavorable zone'). If the conditional power is 0.80 or higher ('favorable zone'), the interim results are considered sufficiently advantageous to achieve significance at the final analysis without the need for a further sample size increase. When the sample size is not increased at the interim analysis, the DMC will not communicate whether this is due to 'unfavorable' or 'favorable results'.

8.4.8 Safety Data

All safety data collected on or after the date that investigational medicinal product was first received up to the date of last dose of investigational medicinal product plus 30 days will be summarized by treatment group (according to the investigational medicinal product received).

8.4.8.1 Extent of Exposure to Study Medication

Descriptive information will be provided by treatment arm regarding the number of doses of Ips administered, the duration of treatment and the cumulative dose received.

8.4.8.2 Adverse Events

All AEs will be listed. The focus of AE summary will be on treatment-emergent AEs. A treatment-emergent AE is defined as an AE that occurs or worsens in the period from the first dose of study treatment to 30 days after the last dose of IP.

AEs will be classified using MedDRA (http://www.meddramsso.com) with descriptions by System Organ Class, High-Level Group Term, High-Level Term, Preferred Term, and Lower-Level Term. The severity of AEs will be graded by the investigator according to the CTCAE, Version 5.0 (https://ctep.cancer.gov/protocoldevelopment/electronic_applications/docs/ctcae_v5_quick_reference_5x7.pdf), whenever possible. If a CTCAE criterion does not exist for a specific type of AE, the grade corresponding to the appropriate adjective will be used by the investigator to describe the maximum intensity of the AE: Grade 1 (mild), Grade 2 (moderate), Grade 3 (severe), Grade 4 (life-threatening), or Grade 5 (fatal). The relationship of the AE to the IP will be categorized as related or unrelated.

Treatment-emergent AEs will be summarized by treatment arm. Summary tables will be presented to show the number of participants reporting treatment-emergent AEs by severity grade and corresponding percentages. A participant who reports multiple treatment-emergent AEs within the same Preferred Term

The confidential information in this document is provided to you as an investigator, potential investigator or consultant for review by you, your staff and applicable Independent Ethics Committee or Review Board. It is understood that the information will not be disclosed to others without written authorization from Biophytis S.A.

(or System Organ Class) is counted only once for that Preferred Term (or System Organ Class) using the worst severity grade. AE descriptions will be presented by decreasing frequency for a given System Organ Class and Preferred Term.

Separate summaries will be prepared for the following types of treatment emergent AEs:

- Fatal events
- Study-medication-related AEs
- AEs that are Grade ≥ 3 in severity
- AEs leading to IP interruption and/or dose modification
- AEs leading to IP permanent discontinuation
- SAEs
- AESI

9 Supporting Documentation and Operational Procedures

9.1 Regulatory, Ethical, and Study Oversight Considerations

The investigator will ensure that this study is conducted according to the protocol and in full conformity with the following applicable regulations:

- Consensus ethical principles derived from international guidelines including the Declaration of Helsinki and Council for International Organizations of Medical Sciences (CIOMS) International Ethical Guidelines
- Applicable ICH Good Clinical Practice (GCP) Guidelines
- Applicable laws and regulations

The protocol, protocol amendments, ICF, Investigator's Brochure, and other relevant documents (e.g., advertisements) must be submitted to an IRB/IEC by the Investigator and reviewed and approved by the IRB/IEC before the study is initiated.

Any amendments to the protocol will require IEC/IRB approval before implementation of changes made to the study design, except for changes necessary to eliminate an immediate hazard to study participants.

The Investigator will be responsible for the following:

- Providing written summaries of the status of the study to the IRB/IEC annually or more frequently in accordance with the requirements, policies, and procedures established by the IRB/IEC
- Notification to the IRB/IEC of SAEs or other significant safety findings as required by IRB/IEC procedures
- Overall conduct of the study at the site and adherence to requirements of 21 CFR, ICH GCP guidelines, the IRB/IEC, European regulation 536/2014 for clinical studies (if applicable), and all other applicable local regulations

9.1.1 General Informed Consent Process

The Investigator or his/her qualified designee must obtain documented consent from each potential participant or each participant's legally acceptable representative prior to participating in a clinical trial and must explain the nature of the study to the participant and his legally authorized representative and answer all questions regarding the study.

The confidential information in this document is provided to you as an investigator, potential investigator or consultant for review by you, your staff and applicable Independent Ethics Committee or Review Board. It is understood that the information will not be disclosed to others without written authorization from Biophytis S.A.

Participants must be informed that their participation is voluntary. Participants and their legally authorized representative (defined as an individual authorized under applicable law to consent on behalf of a prospective participant to their participation in the procedure(s) involved in the research) will be required to sign a statement of informed consent that meets the requirements of 21 CFR 50, local regulations, ICH guidelines, Health Insurance Portability and Accountability Act (HIPAA) requirements, where applicable, and the IRB/IEC or study center.

The medical record must include a statement that written informed consent was obtained before the participant was enrolled in the study and the date the written consent was obtained. The authorized person obtaining the informed consent must also sign the informed consent form (ICF).

The informed consent obtained from the participant will include explicit consent for the collection of historic data from already received standard of care assessments prior to the start of study (obtained on the same day as screening).

A copy of the ICF(s) must be provided to the participant or the participant’s legally authorized representative before participation in the trial.

Participants who are re-screened are required to sign a new ICF.

The Investigator or authorized designee will explain to each participant the objectives of the exploratory research. Participants will be told that they are free to refuse participation and may withdraw their specimens at any time and for any reason during the storage period. A separate signature will be required to document a participant’s agreement to allow any remaining specimens to be used for exploratory research. Participants who decline to participate will not provide this separate signature.

If a protocol amendment is required, the ICF may need to be revised to reflect the changes to the protocol. If the ICF is revised, it must be reviewed and approved by the appropriate IEC/IRB in advance of use, and signed by all participants subsequently enrolled in the study as well as those currently enrolled in the study. The participant or his legally acceptable representative should be informed in a timely manner if new information becomes available that may be relevant to the participant’s willingness to continue participation in the trial.

Participants must be re-consented to the most current version of the ICF(s) during their participation in the study if a protocol amendment takes place or if new pertinent information becomes available.

The informed consent will adhere to IRB/ERC requirements, applicable laws and regulations and Sponsor requirements.

9.1.2 Study Discontinuation and Site Closure

This study may be temporarily suspended or prematurely terminated if there is sufficient reasonable cause. Written notification documenting the reason for study suspension or termination will be provided by the suspending or terminating party to study participants, investigators, funding agency, the Investigational New Drug (IND) or Investigational Device Exemption (IDE) sponsor and regulatory authorities. If the study is prematurely terminated or suspended, the PI will promptly inform study participants, EC/IRB, and sponsor and will provide the reason(s) for the termination or suspension. Study participants will be contacted, as applicable, and be informed of changes to study visit schedule.

Circumstances that may warrant termination or suspension include, but are not limited to:

The confidential information in this document is provided to you as an investigator, potential investigator or consultant for review by you, your staff and applicable Independent Ethics Committee or Review Board. It is understood that the information will not be disclosed to others without written authorization from Biophytis S.A.

- Determination of unexpected, significant, or unacceptable risk to participants
- Demonstration of efficacy that would warrant stopping
- Insufficient compliance to protocol requirements
- Data that are not sufficiently complete and/or evaluable
- Determination that the primary endpoint has been met
- Determination of futility
- Sponsor's decision based on not meeting realistic timelines and resources allocated to the clinical trial

Study may resume once concerns about safety, protocol compliance, and data quality were addressed, and satisfactory to the sponsor, IRB and/or the FDA.

The Sponsor designee also reserves the right to close the study site at any time for any reason at the sole discretion of the Sponsor. The Investigator may also initiate study site closure at any time, provided there is reasonable cause and sufficient notice is given in advance of the intended termination.

Reasons for the early closure of a study site by the Sponsor or Investigator may include, but are not limited to:

- Failure of the Investigator to comply with the protocol, the requirements of the IRB/IEC or local health authorities, the Sponsor's procedures, or GCP guidelines.
- Inadequate recruitment of participants by the Investigator.
- Discontinuation of further study medication development.

Study sites will be closed upon study completion. A study site is considered closed when all required documents and study supplies have been collected and a study site closure visit has been performed.

9.1.3 Confidentiality and Privacy

Participant confidentiality is strictly held in trust by the participating investigators, their staff, and the sponsor and their agents. This confidentiality is extended to cover testing of biological samples. Therefore, the study protocol, documentation, data, and all other information generated will be held in strict confidence. No information concerning the study or the data will be released to any unauthorized third party without prior written approval of the sponsor.

The study monitor, other authorized representatives of the sponsor, or representatives of the IEC or IRB may inspect all documents and records required to be maintained by the investigator, including but not limited to, medical records (office, clinic, or hospital) and pharmacy records for the participants in this study. The clinical study site will permit access to such records.

The study participant's contact information will be securely stored at each clinical site for internal use during the study. At the end of the study, all records will continue to be kept in a secure location for as long a period as dictated by the local IEC/IRB and institutional regulations.

Study participant's research data, which are used for purposes of statistical analysis and scientific reporting, will not include the participant's contact or identifying information. Rather, individual participants and their research data will be identified by a unique study identification number. At the end of the study, all study databases will be de-identified and archived at Biophytis.

The confidential information in this document is provided to you as an investigator, potential investigator or consultant for review by you, your staff and applicable Independent Ethics Committee or Review Board. It is understood that the information will not be disclosed to others without written authorization from Biophytis S.A.

9.1.4 Future Use of Stored Specimens and Data

Data collected for this study will be analyzed and stored by the Sponsor. After the study is completed, the de-identified, archived data could be made available on demand based on previous agreement for use by other researchers or Consortia including those outside of the study. Permission to use and transmit de-identified data for secondary research will be included in the main ICF.

9.1.5 Key Roles and Study Governance

The overall organizational structure of this study will be supervised and managed through the following bodies:

- Managing Board (on behalf of the Sponsor)
- Steering Committee (independent)
- Data Safety Monitoring Board (independent)
- Independent Scientific Advisory Board

This governance structure is meant to guarantee a high level of the scientific quality for this study.

9.1.5.1 The Managing Board

The Managing Board is composed of Sponsor Senior Management and the Principal Investigator.

Main responsibilities:

- Making and implementing strategic decisions as advised by the independent Committees
- Coordinating and optimizing the study resources
- Monitoring the progress of the study activities with respect to its objectives
- Identifying possible issues and proactively proposing solutions
- Coordinating communication activities

Any member of the Managing Board may participate in meetings of the Managing Board by teleconference, video-conference or any other technology that enables everyone participating in the meeting to communicate interactively and effectively with each other and as a group.

For major changes, the Managing Board will consult the SAB, the DMC and the other leaders as applicable.

9.1.5.2 The Steering Committee

The Steering Committee is composed of representative members of the participating countries.

The Steering Committee will be able to invite other experts from Biophytis and Biophytis subcontractors, e.g. clinical operations, clinical development, etc. as deemed necessary for the purpose of the meetings.

9.1.5.3 The Data Monitoring Committee (DMC)

The DMC will periodically review all safety data and raise alerts in case of negative or dangerous findings. Members of the DMC will be selected based on their expertise by the managing Board.

The DMC is also responsible for the conduct of the study interim analyses.

If, while this study is ongoing, a medication should be proven to have a beneficial influence on the prevention of COVID-19 respiratory distress, a specific DMC meeting will be convened, in order to discuss

The confidential information in this document is provided to you as an investigator, potential investigator or consultant for review by you, your staff and applicable Independent Ethics Committee or Review Board. It is understood that the information will not be disclosed to others without written authorization from Biophytis S.A.

either the trial termination, or allocation of the efficacious medication (e.g. antiviral, antimalarial, losartan, other) to all included participants.

More information on the DMC responsibilities is laid out in the DMC charter.

9.1.5.4 The Scientific Advisory Board (SAB)

This is an independent advisory committee, able to advise and discuss the overall study conduct and scientific basis in view of ongoing progress of knowledge within the scientific community. The SAB will may ask DMC questions and recommend specific actions to the other governance committees.

9.1.6 Safety oversight

Safety oversight will be under the direction of a DMC composed of individuals with the appropriate expertise. Members of the DMC should be independent from the study conduct and free of conflict of interest, or measures should be in place to minimize perceived conflict of interest. The DMC will meet based on a pre-determined schedule to assess safety data of all study participants enrolled in the study. The DMSB will operate under the rules of an approved charter that will be written and reviewed at the organizational meeting of the DMC. At this time, each data element that the DMC needs to assess will be clearly defined. The DMC will provide its input to the Study Managing Board and will answer questions from the Steering Committee and the Scientific Advisory Board.

9.1.7 Clinical Monitoring

Clinical site monitoring is conducted to ensure that the rights and well-being of trial participants are protected, that the reported trial data are accurate, complete, and verifiable, and that the conduct of the trial is in compliance with the currently approved protocol/amendment(s), with ICH GCP, and with applicable regulatory requirement(s).

During the study, a monitor will make site visits to review protocol compliance, compare eCRF entries and individual participant’s medical records, assess drug accountability, and ensure that the study is being conducted according to pertinent regulatory requirements. eCRF entries will be verified with source documentation. The review of medical records will be performed in a manner to ensure that participant confidentiality is maintained.

Checking of the eCRF entries for completeness and clarity, and cross-checking with source documents, will be required to monitor the progress of the study. Moreover, regulatory authorities of certain countries, IRBs, IECs, and/or the Sponsor’s Clinical Quality Assurance Group may wish to carry out such source data checks and/or on-site audit inspections. Direct access to source data will be required for these inspections and audits; they will be carried out giving due consideration to data protection and medical confidentiality. The Investigator assures SGS and the Sponsor of the necessary support at all times.

9.1.8 Data Quality Assurance and Record Keeping

Biophytis shall implement and maintain quality control and quality assurance procedures with written standard operating procedures (SOPs) to ensure that the study is conducted and that the data are generated, documented and reported in compliance with the protocol, ICH GCP and applicable regulatory requirements.

The confidential information in this document is provided to you as an investigator, potential investigator or consultant for review by you, your staff and applicable Independent Ethics Committee or Review Board. It is understood that the information will not be disclosed to others without written authorization from Biophytis S.A.

All participant data related to the study will be recorded on printed or eCRF unless transmitted to the Sponsor or designee electronically (e.g., laboratory data). The Investigator is responsible for verifying that data entries are accurate and correct by physically or electronically signing the CRF.

The Investigator must maintain accurate documentation (source data) that supports the information entered in the eCRF.

The Investigator must permit study-related monitoring, audits, IRB/IEC review, and regulatory agency inspections and provide direct access to source data documents.

The Sponsor or designee is responsible for the data management of this study including quality checking of the data.

Study monitors will perform ongoing source data verification to confirm that data entered into the eCRF by authorized site personnel are accurate, complete, and verifiable from source documents; that the safety and rights of participants are being protected; and that the study is being conducted in accordance with the currently approved protocol and any other study agreements, ICH GCP, and all applicable regulatory requirements.

Records and documents, including signed ICF, pertaining to the conduct of this study must be retained by the Investigator for 2 years after last approval of a marketing application unless local regulations or institutional policies require a longer retention period. No records may be destroyed during the retention period without the written approval of the Sponsor. No records may be transferred to another location or party without written notification to the Sponsor.

All data generated by the site personnel will be captured electronically at each study center using eCRFs. Data from external sources (such as laboratory data) will be imported into the database. Once the eCRF clinical data have been submitted to the central server at the independent data center, corrections to the data fields will be captured in an audit trail. The reason for change, the name of the person who performed the change, together with the time and date will be logged to provide an audit trail.

If additional corrections are needed, the responsible monitor or data manager will raise a query in the electronic data capture (EDC) application. The appropriate staff at the study site will answer queries sent to the Investigator. The name of the staff member responding to the query, and time and date stamp will be captured to provide an audit trail. Once all source data verification is complete and all queries are closed, the monitor will freeze the eCRF page.

The specific procedures to be used for data entry and query resolution using the eCRF will be provided to study sites in a training manual. In addition, site personnel will receive training on the EDC/eCRF.

9.1.9 Source Documents

Data collection is the responsibility of the clinical trial staff at the site under the supervision of the site investigator. The investigator is responsible for ensuring the accuracy, completeness, legibility, and timeliness of the data reported.

All source documents should be completed in a neat and legible manner to ensure accurate interpretation of data. Source data are all informational and original records of clinical findings, observations, or other activities in a clinical trial necessary for the reconstruction and evaluation of the trial. Electronic source data are data initially recorded in electronic form. Examples of source data include, but are not limited to: hospital records, clinical and office charts, laboratory notes, memoranda, participants' memory aids or

The confidential information in this document is provided to you as an investigator, potential investigator or consultant for review by you, your staff and applicable Independent Ethics Committee or Review Board. It is understood that the information will not be disclosed to others without written authorization from Biophytis S.A.

evaluation checklists, pharmacy dispensing records, audio recordings of counseling sessions, recorded data from automated instruments, copies or transcriptions certified after verification as being accurate and complete, microfiches, photographic negatives, microfilm or magnetic media, x-rays, and participant files and records kept at the pharmacy, at the laboratories, and medico-technical departments involved in the clinical trial. It is acceptable to use CRFs as source documents. If this is the case, it should be stated in this section what data will be collected on CRFs and what data will be collected from other sources.

It is not acceptable for the CRF to be the only record of a participant's inclusion in the study. Study participation should be captured in a participant's medical record. This is to ensure that anyone who would access the participant medical record has adequate knowledge that the participant is participating in a clinical trial.

Data recorded in the (eCRF) derived from source documents should be consistent with the data recorded on the source documents.

9.1.10 Study Records Retention

The investigator/institution should maintain the study documents as specified in the ICH guidelines of GCP and as required by the applicable regulatory requirements. The investigator/institution should take measures to prevent accidental or premature destruction of these documents.

Essential documents should be retained until at least 2 years after the last approval of a marketing application in an ICH region and until there are no pending or contemplated marketing applications in an ICH region or until at least 2 years have elapsed since the formal discontinuation of clinical development of the investigational product. These documents should be retained for a longer period, however, if required by the applicable regulatory requirements or by an agreement with the sponsor. It is the responsibility of the sponsor to inform the investigator/institution as to when these documents no longer need to be retained.

9.1.11 Protocol Deviations

A protocol deviation is any noncompliance with the clinical trial protocol or the GCP. Further details about the handling of protocol deviations will be included in the MOP.

The noncompliance may be either on the part of the participant, the investigator, the study-site staff or the Sponsor. As a result of deviations, corrective actions are to be developed by the site and implemented promptly.

These practices are consistent with ICH GCP:

- 4.5 Compliance with Protocol, sections 4.5.1, 4.5.2, and 4.5.3
- 5.1 Quality Assurance and Quality Control, section 5.1.1
- 5.20 Noncompliance, sections 5.20.1, and 5.20.2.

No changes from the final approved and signed protocol will be initiated without the prior written approval or favorable opinion of a written amendment by the IEC/IRB and applicable local competent authorities, except when necessary to address immediate safety concerns to the participants or when the change involves only non-substantial logistics or administration. Each investigator and the sponsor will sign the protocol amendment.

The confidential information in this document is provided to you as an investigator, potential investigator or consultant for review by you, your staff and applicable Independent Ethics Committee or Review Board. It is understood that the information will not be disclosed to others without written authorization from Biophytis S.A.

It is the responsibility of the Investigator to use continuous vigilance to identify and report all deviations to Biophytis and the local IRC/IRB according to their guidelines. The site PI/study staff is responsible for knowing and adhering to their IEC/IRB requirements.

9.1.12 Publication and Data Sharing Policy

The sponsor shall retain the ownership of all data. When the study is complete, the sponsor shall arrange the analysis and tabulation of data. A clinical study report shall then be prepared, which may be used for publication, presentation at scientific meetings or submission to regulatory authorities. All proposed publications based on this study must be subject to the sponsor's approval requirements.

9.1.13 Conflict of Interest Policy

The independence of this study from any actual or perceived influence, such as by the pharmaceutical industry, is critical. Therefore, any actual conflict of interest of persons who have a role in the design, conduct, analysis, publication, or any aspect of this trial will be disclosed and managed. Furthermore, persons who have a perceived conflict of interest will be required to have such conflicts managed in a way that is appropriate for their participation in the trial. The study leadership, in conjunction with Biophytis, will establish policies and procedures for all study group members to disclose all conflicts of interest and will establish a mechanism for the management of all reported dualities of interest.

The confidential information in this document is provided to you as an investigator, potential investigator or consultant for review by you, your staff and applicable Independent Ethics Committee or Review Board. It is understood that the information will not be disclosed to others without written authorization from Biophytis S.A.

10 List of Tables

Table 1: Summary of medications that are being tested for COVID-19 34

Table 2: Pharmacokinetic (PK) measurements 46

Table 3: The Operating Principals of the COVA Study 50

Table 4: COVA Study Decisions and Implementation 51

Table 5: COVA Study Interim Analysis Information Summary 51

Table 6: Pop-PK study - schedule of blood draws 55

Table 7: Parameters that are analyzed at the local lab 66

Table 8: Parameters that are analyzed at the central lab 66

Table 9: Severity assessment, according to CTCAE 68

Table 10: Causality assessment, according to WHO 68

Table 11: Sample Size Considerations 77

11 List of Figures

Figure 1: COVA Study Scheme 30

Figure 2: The Mechanism, by Which SARS-CoV-2 Causes Respiratory Deterioration in COVID-19 and the Potential Role of BIO101 41

The confidential information in this document is provided to you as an investigator, potential investigator or consultant for review by you, your staff and applicable Independent Ethics Committee or Review Board. It is understood that the information will not be disclosed to others without written authorization from Biophytis S.A.

12 Abbreviations and Definitions

**20E**	20-Hydroxyecdysone
**ABG**	Arterial Blood Gasses
**ACE2**	Angiotensin-Converting Enzyme 2
**ACEi**	Angiotensin-converting-enzyme inhibitor
**ADL**	Activities of daily living
**AE**	Adverse Event
**AESI**	Adverse Events of Special Interest
**ALI**	Acute Lung Injury
**ALT**	Alanine Aminotransferase
**Ang**	Angiotensin
**Anti-IL6-Mabs**	Anti-Interleukin 6-monoclonal antibody
**APACHE II**	Acute Physiology and Chronic Health Evaluation II
**ARB**	Angiotensin Receptor Blocker
**ARDS**	Acute Respiratory Distress Syndrome
**AST**	Aspartate Aminotransferase
**AT1**	Angiotensin II receptor type 1
**AUC**	Area Under the Curve
**b.i.d.**	Twice a day
**CD24**	Cluster of differentiation (24) that identify cell type and stage of differentiation
**CFR**	Case Report Form
**CI**	Confidence Intervals
**CIOMS**	Council for International Organizations of Medical Sciences
**CK**	Creatine kinase
**CK-MB**	Myoglobin and creatine kinase
**C_{max}**	Maximum serum concentration that a drug achieves
**CMC IMPD**	Chemistry, Manufacturing and Controls Investigational Medicinal Product Dossier
**CMH**	Cochran-Mantel-Haenszel test
**CoA**	Certificate of Analysis
**COVID-19**	Coronavirus Disease of 2019
**CPAP**	Continuous Positive Airway Pressure
**CRO**	Clinical Research Organization
**CT**	Computer Tomography
**CTCAE**	Common Terminology Criteria for Adverse Events
**D**	Day

The confidential information in this document is provided to you as an investigator, potential investigator or consultant for review by you, your staff and applicable Independent Ethics Committee or Review Board. It is understood that the information will not be disclosed to others without written authorization from Biophytis S.A.

**DMC**	Data Monitoring Committee
**DMD**	Duchenne muscular dystrophy
**DSMB**	Data Safety Monitoring Board
**ECG**	Electrocardiogram
**ECMO**	Extracorporeal Membrane Oxygenation
**eCRF**	electronic Case Report Form
**EDC**	Electronic data capture
**eGFR**	Estimated Glomerular Filtration Rate
**FA**	Final analysis
**Fc**	Fragment Crystallizable region
**FDA**	Food and Drug Administration
**FiO_2**	Fraction of inspired oxygen
**FSH**	Follicle-stimulating hormone
**GCP**	Good Clinical Practice
**GFR**	Glomerular Filtration Rate
**GGT**	Gamma-Glutamyl Transferase
**GLDH**	Glutamate dehydrogenase
**GMP**	Good Manufacturing Practice
**H0**	Null hypothesis
**Ha**	Alternative hypothesis

**HCP**	Healthcare Provider
**High-flow oxygen**	High-flow oxygen is defined as delivery of oxygen at a flow of ≥16 L/min.
**HIPAA**	Health Insurance Portability and Accountability Act
**HIV**	Human Immunodeficiency Virus
**IA**	Interim analysis
**IB**	Investigator's brochure
**IC50**	Half maximal inhibitory concentration
**ICH**	International Council for Harmonization
**ICU**	Intensive Care Unit
**IDE**	Investigational Device Exemption
**IEC**	International Electrotechnical Commission
**IGF1**	Insulin-like Growth Factor 1
**IND**	Investigational New Drug
**INR**	International normalized ratio
**IP**	Investigational product
**IPC**	Infection Prevention and Control
**IRB**	Institutional Review Board
**ITT**	Intention-to-treat analysis

The confidential information in this document is provided to you as an investigator, potential investigator or consultant for review by you, your staff and applicable Independent Ethics Committee or Review Board. It is understood that the information will not be disclosed to others without written authorization from Biophytis S.A.

**IWRS**	Interactive Web Response System
**LFT**	Liver Function Test
**LH**	Luteinizing hormone
**LLN**	Lower limit of the normal range
**MAD**	Multiple Administration Dose
**MOP**	Manual of Procedures
**MSC**	Mesenchymal stem-cell
**NewS2**	National Early Warning Score 2
**NOAEL**	No Observed Adverse Effect Level
**OTH**	Orthostatic hypotension
**P0**	Null proportion
**Pa**	Alternative proportion
**PCR**	Polymerase Chain Reaction
**Penh**	Enhanced pause
**PI**	Principal Investigator
**PoC**	Proof of Concept
**PORT score**	Pneumonia Patient Outcomes Research Team (PORT) Severity Index
**PP**	Per-protocol analysis
**q.d.**	Once a day

**QT**	Time from the start of the Q wave to the end of the T wave on ECG
**QTcF**	Corrected QT interval by Fredericia
**RAC**	Recombinant DNA Advisory Committee
**RAS**	Renin-Angiotensin System
**RNA**	Ribonucleic Acid
**SAB**	Scientific Advisory Board
**SAD**	Single Administration Dose
**SAE**	Serious Adverse Event
**SafP**	Safety Analysis Set (population)
**SpO_2**	Oxygen saturation in arterial blood
**SAP**	Study Analysis Protocol
**SARA-INT**	SARA-Interventional (study)
**SARA-PK**	SARA-Pharmacokinetic (study)
**SARS-CoV-2**	Severe Acute Respiratory Syndrome Coronavirus 2
**SBP**	Systolic Blood Pressure
**SUSAR**	Suspected Unexpected Serious Adverse Reaction
**T**	Time
**t.i.d.**	3 times a day

The confidential information in this document is provided to you as an investigator, potential investigator or consultant for review by you, your staff and applicable Independent Ethics Committee or Review Board. It is understood that the information will not be disclosed to others without written authorization from Biophytis S.A.

**TLR**	Toll-like Receptor
**TMF**	Trial Master File
**TNF**	Tumor Necrose Factor
**TU**	Therapeutic Unit
**ULN**	Upper Limits of Normal
**Ups**	Unanticipated Problems

**US**	Ultrasound
**VIDD**	Ventilator-Induced Diaphragmatic Dysfunction
**VILI**	Ventilator-Induced Lung Injury
**VIP**	Vasoactive Intestinal Peptide
**WHO**	World Health Organization

The confidential information in this document is provided to you as an investigator, potential investigator or consultant for review by you, your staff and applicable Independent Ethics Committee or Review Board. It is understood that the information will not be disclosed to others without written authorization from Biophytis S.A.

13 References

---

[1] Liu Y, Gayle AA, Wilder-Smith A, Rocklöv J. The reproductive number of COVID-19 is higher compared to SARS coronavirus. J Travel Med. 2020;27(2):taaa021. doi:10.1093/jtm/taaa021

[2] Wu Z, McGoogan JM. Characteristics of and Important Lessons From the Coronavirus Disease 2019 (COVID-19) Outbreak in China: Summary of a Report of 72 314 Cases From the Chinese Center for Disease Control and Prevention [published online ahead of print, 2020 Feb 24]. JAMA. 2020;10.1001/jama.2020.2648. doi:10.1001/jama.2020.2648

[3] Verity R, Okell LC, Dorigatti I, Winskill P, Whittaker C, Imai N, Cuomo-Dannenburg G, Thompson H, Walker P, Fu H, Dighe A, Griffin J, Cori A, Baguelin M, Bhatia S, Boonyasiri A, Cucunuba ZM, Fitzjohn R, Gaythorpe KAM, Green W, Hamlet A, Hinsley W, Laydon D, Nedjati-Gilani G, Riley S, van-Elsand S, Volz E, Wang H, Wang Y, Xi X, Donnelly C, Ghani A, Ferguson N. Estimates of the severity of COVID-19 disease med; Rxiv 2020.03.09.20033357; doi: https://doi.org/10.1101/2020.03.09.20033357

[4] Zhou F, Yu T, Du R, et al. Clinical course and risk factors for mortality of adult inpatients with COVID-19 in Wuhan, China: a retrospective cohort study [published correction appears in Lancet. 2020 Mar 28;395(10229):1038] [published correction appears in Lancet. 2020 Mar 28;395(10229):1038]. Lancet. 2020;395(10229):1054–1062. doi:10.1016/S0140-6736(20)30566-3

[5] Zhou P, Yang XL, Wang XG, et al. A pneumonia outbreak associated with a new coronavirus of probable bat origin. Nature. 2020; 579: 270-273

[6] Hoffmann M, Kleine-Weber H, Schroeder S, Krüger N, Herrler T, Erichsen S, Schiergens TS, Herrler G, Wu NH, Nitsche A, Müller MA, Drosten C, Pöhlmann S. SARS-CoV-2 Cell Entry Depends on ACE2 and TMPRSS2 and Is Blocked by a Clinically Proven Protease Inhibitor. Cell. 2020, pii: S0092-8674(20)30229-4. doi: 10.1016/j.cell.2020.02.052

Wan Y, Shang J, Graham R, Baric RS, Li F. Receptor recognition by novel coronavirus from Wuhan: An analysis based on decade-long structural studies of SARS. Journal of Virology 2020

Xu X., Chen P., Wang J., Feng J., Zhou H., Li X., Zhong W., Hao P.. Evolution of the novel coronavirus from the ongoing Wuhan outbreak and modeling of its spike protein for risk of human transmission. *Science China Life Sciences* volume 63, pages 457 – 460 (2020).

[7] Magalhaes GS, Barroso LC, Reis AC, Rodrigues-Machado MG, Gregorio JF, Motta-Santos D, et al. Angiotensin-(1–7) Promotes Resolution of Eosinophilic Inflammation in an Experimental Model of Asthma. Front Immunol. 2018; 9:58

Jiang T1, Gao L, Shi J, Lu J, Wang Y, Zhang Y. Angiotensin-(1-7) modulates renin-angiotensin system associated with reducing oxidative stress and attenuating neuronal apoptosis in the brain of hypertensive rats. Pharmacol Res. 2013 Jan;67(1):84-93

van Twist DJ, Houben AJ, de Haan MW, Mostard GJ, Kroon AA, de Leeuw PW. Angiotensin-(1-7)-induced renal vasodilation in hypertensive humans is attenuated by low sodium intake and angiotensin II co-infusion. Hypertension. 2013 Oct;62(4):789-93

[8] Santos RA., Ferreira A.J., Verano-Braga T., Bader M. Angiotensin-converting enzyme 2, angiotensin-(1-7) and Mas: new players of the renin-angiotensin system. J Endocrinol. 2013 Jan 18;216(2): R1-R17

[9] Kuba K, Imai Y, Rao S, Gao H, Guo F, Guan B, Huan Y, Yang P, Zhang Y, Deng W, Bao L, Zhang B, Liu G, Wang Z, Chappell M, Liu Y, Zheng D, Leibbrandt A, Wada T, Slutsky AS, Liu D, Qin C, Jiang C, Penninger JM. A crucial role of angiotensin converting enzyme 2 (ACE2) in SARS coronavirus-induced lung injury. Nat.Med. 2005; 11:875–879.

Imai Y, Kuba K, Rao S, Huan Y, Guo F, Guan B, Yang P, Sarao R, Wada T, Leong-Poi H, Crackower MA, Fukamizu A, Hui CC, Hein L, Uhlig S, Slutsky AS, Jiang C, Penninger JM. Angiotensin-converting enzyme 2 protects from severe acute lung failure. Nature. 2005 Jul 7;436(7047):112-6

[10] Zhang H, Baker A. Recombinant human ACE2: acing out angiotensin II in ARDS therapy. Crit Care. 2017;21(1):305.

The confidential information in this document is provided to you as an investigator, potential investigator or consultant for review by you, your staff and applicable Independent Ethics Committee or Review Board. It is understood that the information will not be disclosed to others without written authorization from Biophytis S.A.

[11] Wu Z, Hu R, Zhang C, Ren W, Yu A, Zhou X Elevation of plasma anfiotensin II level is a potential pathogenesis for the critically ill COVID-19 patients. (2020) *Critical Care* 24:290.

[12] Liu Y, Yang Y, Zhang C, et al. Clinical and biochemical indexes from 2019-nCoV infected patients linked to viral loads and lung injury. Sci China Life Sci. 2020;63(3):364-374.

[13] Bastos A.C., Magalhães G.S., Gregório J.F., Matos N.A., Motta-Santos D., Bezerra F.S., Santos R.A.S., Campagnole Santos M.J., Rodrigues-Machado M.G. Oral formulation angiotensin-(1-7) therapy attenuates pulmonary and systemic damage in mice with emphysema induced by elastase. Immunobiology. 2019 Dec 4:151893.

[14] Shao M., Wen Z.B., Yang H.H., Zhang C.Y., Xiong J.B., Guan X.X., Zhong W.J., Jiang H.L., Sun C.C., Luo X.Q., He X.F., Zhou Y., Guan C.X. Exogenous angiotensin (1-7) directly inhibits epithelial-mesenchymal transformation induced by transforming growth factor-β1 in alveolar epithelial cells. Biomed Pharmacother. 2019 Sep, 117: 109193.

[15] Daniell H., Mangu V., Yakubov B., Park J., Habibi P., Shi Y., Gonnella P.A., Fisher A., Cook T., Zeng L., Kawut S.M., Lahm T. Investigational new drug enabling angiotensin oral-delivery studies to attenuate pulmonary hypertension. Biomaterials. 2020 Mar, 233: 119750.

[16] Ye R., Liu Z. ACE2 exhibits protective effects against LPS-induced acute lung injury in mice by inhibiting the LPS-TLR4 pathway. Exp Mol Pathol. 2020 Apr, 113: 104350.

[17] Zhang Y., Li Y., Shi C., Fu X., Zhao L., Song Y. Angiotensin-(1-7)-mediated Mas1 receptor/NF-κB-p65 signaling is involved in a cigarette smoke-induced chronic obstructive pulmonary disease mouse model. Environ Toxicol. 2018 Jan;33(1):5-15.

[18] Kostis JB, Kim HJ, Rusnak J, Casale T, Kaplan A, Corren J, et al. Incidence and characteristics of angioedema associated with enalapril. Arch Intern Med. 2005 Jul;165((14)):1637–42.

[19] Wang X., Ye Y., Gong H., Wu J., Yuan J., Wang S., Yin P., Ding Z., Kang L., Jiang Q., Zhang W., Li Y., Ge J., Zou Y. The effects of different angiotensin II type 1 receptor blockers on the regulation of the ACE-AngII-AT1 and ACE2-Ang(1-7)-Mas axes in pressure overload-induced cardiac remodeling in male mice. J Mol Cell Cardiol. 2016, 97:180-90

Klimas J., Olvedy M., Ochodnicka-Mackovicova K., Kruzliak P., Cacanyiova S., Kristek F., Krenek P., Ochodnicky P. Perinatally administered losartan augments renal ACE2 expression but not cardiac or renal Mas receptor in spontaneously hypertensive rats. J Cell Mol Med. 2015 Aug, 19(8):1965-74.

[20] Dioh W, Tourette C, Margalef C, Chen A, Lafont, Dilda P, Veillet S, Agus S. SARA program: preliminary findings & implications from SARA-OBS study and its impact on SARA-INT study, oral presentation at the International Conference on Frailty and Sarcopenia Research (ICFSR, 2020), March 11-13, 2020, Toulouse, France

[21] Chabane M, Dioh W, Dilda P, Lafont R, Veillet S, Voit T, Agus S, The MYODA Operational Seamless clinical trial design phase I to III: A new approach for Rare Diseases to Evaluate the Safety, Efficacy, Pharmacokinetics, and Pharmacodynamics of BIO101 (MAS Activator) in Paediatric Patients with a Genetically Confirmed Diagnosis of Duchenne Muscular Dystrophy (DMD) & MYODA clinical program: Composite score for assessing the efficacy of BIO101 (MAS activator) in ambulatory and non-ambulatory Duchenne boys. Poster presentations at theWorld Muscle Society Conference (WMS 2019), Copenhagen, Denmark

[22] Dinan L., Lafont R. Effects and applications of arthropod steroid hormones (ecdysteroids) in mammals. J Endocrinol. 2006 Oct;191(1):1-8.

[23] Song G, Xia XC, Zhang K, Yu R, Li B, Li M, Yu X, Zhang J, Xue S. 2019. Protective effect of 20-hydroxyecdysterone against lipopolysaccharides-induced acute lung injury in mice. Journal of Pharmaceutics and Drug Research 2 (3): 109-114.

[24] Song G, Xia XC, Zhang K, Yu R, Li B, Li M, Yu X, Zhang J, Xue S. 2019. Protective effect of 20-hydroxyecdysterone against lipopolysaccharides-induced acute lung injury in mice. Journal of Pharmaceutics and Drug Research 2 (3): 109-114.

The confidential information in this document is provided to you as an investigator, potential investigator or consultant for review by you, your staff and applicable Independent Ethics Committee or Review Board. It is understood that the information will not be disclosed to others without written authorization from Biophytis S.A.

[25] Wu X, Wu X, Feng C, Li J, Gu C, Shen T, Gao D 2012. Effects of ecdysterone on the ultrastructure and IL-10 mRNA expression of lung tissue in rats with acute lung injury. *Infection, Inflammation, Repair* 13 (04): 207-211 + 257

[26] Li J, Wu X, Zhang J, Wu X, Gao D, Shen T, Gu C. Effect of ecdysterone on the expression of toll-like receptor 4 and surfactant protein A in lung tissue of rats with acute lung injury. *Infection Inflammation Repair*, 2013 (1), 22-26.

[27] Henderson W.R., Chen L., Amato M.B.P., Brochard L.J. Fifty Years of Research in ARDS. Respiratory Mechanics in Acute Respiratory Distress Syndrome. Am J Respir Crit Care Med. 2017 Oct 1;196(7):822-833.

[28] Dilda P, Latil M, Didry-Barca B, On S, Serova M, Mamchaoui K, Veillet S, Lafont R (2019b). BIO101 demonstrates combined beneficial effects on skeletal muscle and respiratory functions in a mouse model of Duchenne muscular dystrophy. 24th WMS Congress, 1-5th Oct, Copenhagen, Denmark, *Neuromuscular Disorders*, 29, S1, S158, abstract P311.

[29] Dreyfuss D, Saumon G. Ventilator-induced lung injury: lessons from experimental studies. Am J Respir Crit Care Med, 1998. 157:294–323

[30] Powers SK, Wiggs MP, Sollanek KJ, Smuder A. Ventilator-induced diaphragm dysfunction: cause and effect. Am J Physiol Regul Integr Comp Physiol, 2013. 305: R464–R477.

[31] Sigurta A, Zambelli V, Bellani G. Medical Hypotheses, 2016. Renin-angiotensin system in ventilator-induced diaphragmatic dysfunction: Potential protective role of Angiotensin (1–7). 94; 132–137.

[32] Rezk BM, Yoshida T, Semprun-Prieto L, Higashi Y, Sukhanov S, Delafontaine P. Angiotensin II infusion induces marked diaphragmatic skeletal muscle atrophy. 2012, PLoS One 7:e30276.

[33] Zambelli V, Sigurtà A, Rizzi L, Zucca L, Delvecchio P, Bresciani E, Torsello A and Bellani G. Angiotensin-(1–7) exerts a protective action in a rat model of ventilator-induced diaphragmatic dysfunction Intensive Care Medicine Experimental (2019) 7:8.

[34] Zhou F, Yu T, Du R, et al. Clinical course and risk factors for mortality of adult inpatients with COVID-19 in Wuhan, China: a retrospective cohort study [published correction appears in Lancet. 2020 Mar 28;395(10229):1038] [published correction appears in Lancet. 2020 Mar 28;395(10229):1038]. Lancet. 2020;395(10229):1054–1062. doi:10.1016/S0140-6736(20)30566-3 Copy

[35] Stewart J, Turner KJ. Inhibin B as a potential biomarker of testicular toxicity. Cancer Biomark. 2005;1(1):75-91. doi:10.3233/cbm-2005-1109

[36] Esposito S, Cofini M, Rigante D, et al. Inhibin B in healthy and cryptorchid boys. Ital J Pediatr. 2018;44(1):81. Published 2018 Jul 16. doi:10.1186/s13052-018-0523-8

[37] Demyashkin GA. Inhibin B in seminiferous tubules of human testes in normal spermatogenesis and in idiopathic infertility. Syst Biol Reprod Med. 2019;65(1):20-28. doi:10.1080/19396368.2018.1478470

[38] Liu Y, Gayle AA, Wilder-Smith A, Rocklöv J. The reproductive number of COVID-19 is higher compared to SARS coronavirus. J Travel Med. 2020;27(2):taaa021. doi:10.1093/jtm/taaa021

[39] Smith GB, Prytherch DR, Meredith P, Schmidt PE, Featherstone PI. The ability of the National Early Warning Score (NEWS) to discriminate patients at risk of early cardiac arrest, unanticipated intensive care unit admission, and death. Resuscitation. 2013;84(4):465–470. doi:10.1016/j.resuscitation.2012.12.016

[40] Knaus WA, Draper EA, Wagner DP, Zimmerman JE. APACHE II: a severity of disease classification system. *Crit Care Med*. 1985;13(10):818–829.

[41] Donahoe L, McDonald E, Kho ME, Maclennan M, Stratford PW, Cook DJ. Increasing reliability of APACHE II scores in a medical-surgical intensive care unit: a quality improvement study. Am J Crit Care. 2009;18(1):58–64. doi:10.4037/ajcc2009757

The confidential information in this document is provided to you as an investigator, potential investigator or consultant for review by you, your staff and applicable Independent Ethics Committee or Review Board. It is understood that the information will not be disclosed to others without written authorization from Biophytis S.A.

[42] CDC COVID-19 Response Team. Severe Outcomes Among Patients with Coronavirus Disease 2019 (COVID-19) - United States, February 12-March 16, 2020. MMWR Morb Mortal Wkly Rep. 2020;69(12):343-346. Published 2020 Mar 27. doi:10.15585/mmwr.mm6912e2

[43] Verity R, Okell LC, Dorigatti I, et al. Estimates of the severity of coronavirus disease 2019: a model-based analysis [published correction appears in Lancet Infect Dis. 2020 Apr 15;:] [published correction appears in Lancet Infect Dis. 2020 May 4;:]. Lancet Infect Dis. 2020;20(6):669-677. doi:10.1016/S1473-3099(20)30243-7

[44] Zhang J, Wang X, Jia X, et al. Risk factors for disease severity, unimprovement, and mortality in COVID-19 patients in Wuhan, China. Clin Microbiol Infect. 2020;26(6):767-772.doi:10.1016/j.cmi.2020.04.012

[45] https://www.cdc.gov/covid-data-tracker/index.html#demographics

[46] Grasselli G, Zangrillo A, Zanella A, et al. Baseline Characteristics and Outcomes of 1591 Patients Infected With SARS-CoV-2 Admitted to ICUs of the Lombardy Region, Italy [published online ahead of print, 2020 Apr 6]. JAMA. 2020;323(16):1574-1581. doi:10.1001/jama.2020.5394

[47] Livingston E, Bucher K. Coronavirus Disease 2019 (COVID-19) in Italy [published online ahead of print, 2020 Mar 17]. JAMA. 2020;10.1001/jama.2020.4344. doi:10.1001/jama.2020.4344

[48] Nikolich-Zugich J, Knox KS, Rios CT, Natt B, Bhattacharya D, Fain MJ. SARS-CoV-2 and COVID-19 in older adults: what we may expect regarding pathogenesis, immune responses, and outcomes [published correction appears in Geroscience. 2020 May 3;:]. Geroscience. 2020;42(2):505-514. doi:10.1007/s11357-020-00186-0

[49] Zheng Z, Peng F, Xu B, et al. Risk factors of critical & mortal COVID-19 cases: A systematic literature review and meta-analysis [published online ahead of print, 2020 Apr 23]. J Infect. 2020;S0163-4453(20)30234-6. doi:10.1016/j.jinf.2020.04.021

[50] Zhou F, Yu T, Du R, et al. Clinical course and risk factors for mortality of adult inpatients with COVID-19 in Wuhan, China: a retrospective cohort study [published correction appears in Lancet. 2020 Mar 28;395(10229):1038] [published correction appears in Lancet. 2020 Mar 28;395(10229):1038]. Lancet. 2020;395(10229):1054-1062. doi:10.1016/S0140-6736(20)30566-3 Copy

[51] Meftahi GH, Jangravi Z, Sahraei H, Bahari Z. The possible pathophysiology mechanism of cytokine storm in elderly adults with COVID-19 infection: the contribution of "inflame-aging" [published online ahead of print, 2020 Jun 11]. Inflamm Res. 2020;1-15. doi:10.1007/s00011-020-01372-8

[52] Bourgonje AR, Abdulle AE, Timens W, et al. Angiotensin-converting enzyme 2 (ACE2), SARS-CoV-2 and the pathophysiology of coronavirus disease 2019 (COVID-19) [published online ahead of print, 2020 May 17]. J Pathol. 2020;10.1002/path.5471. doi:10.1002/path.5471

[53] Cheng H, Wang Y, Wang GQ. Organ-protective effect of angiotensin-converting enzyme 2 and its effect on the prognosis of COVID-19. J Med Virol. 2020;92(7):726-730. doi:10.1002/jmv.25785

[54] Mueller AL, McNamara MS, Sinclair DA. Why does COVID-19 disproportionately affect older people? Aging (Albany NY). 2020;12(10):9959-9981. doi:10.18632/aging.103344

[55] Nikolich-Zugich J, Knox KS, Rios CT, Natt B, Bhattacharya D, Fain MJ. SARS-CoV-2 and COVID-19 in older adults: what we may expect regarding pathogenesis, immune responses, and outcomes [published correction appears in Geroscience. 2020 May 3;:]. Geroscience. 2020;42(2):505-514. doi:10.1007/s11357-020-00186-0

[56] Oranger M, Gonzalez-Bermejo J, Dacosta-Noble P, et al. Continuous positive airway pressure to avoid intubation in SARS-CoV-2 pneumonia: a two-period retrospective case-control study [published online ahead of print, 2020 May 19]. Eur Respir J. 2020;2001692. doi:10.1183/13993003.01692-2020

The confidential information in this document is provided to you as an investigator, potential investigator or consultant for review by you, your staff and applicable Independent Ethics Committee or Review Board. It is understood that the information will not be disclosed to others without written authorization from Biophytis S.A.

[57] Wang Y, Fan G, Horby P, et al. Comparative Outcomes of Adults Hospitalized With Seasonal Influenza A or B Virus Infection: Application of the 7-Category Ordinal Scale. Open Forum Infect Dis. 2019;6(3):ofz053. Published 2019 Feb 15. doi:10.1093/ofid/ofz053

[58] Meyboom RHB, Royer RJ. Causality Classification in Pharmacovigilance Centres in the European Community. Pharmacoepidemiology and Drug Safety 1992; 1:87-97

[59] Mehta CR, Pocock SJ. Adaptive increase in sample size when interim results are promising: a practical guide with examples. Stat Med. 2011;30(28):3267-3284. doi:10.1002/sim.4102

The confidential information in this document is provided to you as an investigator, potential investigator or consultant for review by you, your staff and applicable Independent Ethics Committee or Review Board. It is understood that the information will not be disclosed to others without written authorization from Biophytis S.A.

Signature Page for TMF-186334 v10.0

Reason for signing: Approved	Name: Sam Agus<br>Role: Sponsor<br>Date of signature: 24-Sep-2020 13:16:03 GMT+0000

Reason for signing: Approved	Name: Waly Dioh<br>Role: Sponsor<br>Date of signature: 24-Sep-2020 19:54:46 GMT+0000

Signature Page for TMF-186334 v10.0
